# MYPT1/PP1‐Mediated EZH2 Dephosphorylation at S21 Promotes Epithelial–Mesenchymal Transition in Fibrosis through Control of Multiple Families of Genes

**DOI:** 10.1002/advs.202105539

**Published:** 2022-03-16

**Authors:** Lan Zhang, Ling Wang, Xue‐Bin Hu, Min Hou, Yuan Xiao, Jia‐Wen Xiang, Jie Xie, Zhi‐Gang Chen, Tian‐Heng Yang, Qian Nie, Jia‐Ling Fu, Yan Wang, Shu‐Yu Zheng, Yun‐Fei Liu, Yu‐Wen Gan, Qian Gao, Yue‐Yue Bai, Jing‐Miao Wang, Rui‐Li Qi, Ming Zou, Qin Ke, Xing‐Fei Zhu, Lili Gong, Yizhi Liu, David Wan‐Cheng Li

**Affiliations:** ^1^ The State Key Laboratory of Ophthalmology Zhongshan Ophthalmic Center Sun Yat‐sen University #54 Xianlie South Road Guangzhou Guangdong 510060 China

**Keywords:** anterior subcapsular cataract (ASC), cataract, dephosphorylation, epithelial‐mesenchymal transition (EMT), enhancer of zeste homolog 2 (EZH2), lens, MYPT1, posterior subcapsular cataract (PCO), POSTN, protein phosphotase‐1

## Abstract

The methyltransferase EZH2 plays an important role in regulating chromatin conformation and gene transcription. Phosphorylation of EZH2 at S21 by AKT kinase suppresses its function. However, protein phosphatases responsible for the dephosphorylation of EZH2‐S21 remain elusive. Here, it is demonstrated that EZH2 is highly expressed in the ocular lens, and AKT‐EZH2 axis is important in TGF*β*‐induced epithelial‐mesenchymal transition (EMT). More importantly, it is identified that MYPT1/PP1 dephosphorylates EZH2‐S21 and thus modulates its functions. MYPT1 knockout accelerates EMT, but expression of the EZH2‐S21A mutant suppresses EMT through control of multiple families of genes. Furthermore, the phosphorylation status and gene expression modulation of EZH2 are implicated in control of anterior subcapsular cataracts (ASC) in human and mouse eyes. Together, the results identify the specific phosphatase for EZH2‐S21 and reveal EZH2 dephosphorylation control of several families of genes implicated in lens EMT and ASC pathogenesis. These results provide important novel information in EZH2 function and regulation.

## Graphical abstract

MYPT1/PP1 is identified as the specific protein phosphatase for dephosphorylating histone methyltransferase enhancer of zeste homolog 2 (EZH2) at S21 residue, demonstrating that EZH2 dephosphorylation/EZH2‐S21A expression causes global changes in multiple families of genes implicated in epithelial‐mesenchymal transition (EMT), contributing to fibrotic diseases, and revealing that AKT‐EZH2‐H3K27Me3 signal axis plays an important role in lens EMT induced by TGF*β* and mediates pathogenesis of anterior subcapsular cataract (ASC).

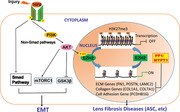

## 1 Introduction

The histone methyltransferase enhancer of Zeste Homolog 2 (EZH2) functions as the enzymatic core component (also including Suzl2 and Eed) of Polycomb Repressive Complex 2 (PRC2) to catalyze histone 3 lysine 27 trimethylation (H3K27Me3) and thus inhibit downstream gene transcription at the epigenetic level.^[^ ^1^ ^]^ Through the control of different target genes, EZH2 has been found to control cellular physiological processes, such as cell proliferation,^[^ ^2^, ^3^, ^4^ ^]^ differentiation,^[^ ^5^, ^6^ ^]^ and senescence,^[^ ^7^, ^8^ ^]^ as well as disease pathogenesis, including tumorigenesis,^[^ ^2^, ^4^ ^]^ cardiovascular diseases,^[^ ^9^, ^10^ ^]^ and inflammation.^[^ ^11^, ^12^, ^13^, ^14^ ^]^ On the other hand, EZH2 can also act as a nonhistone methyltransferase independent of the PRC2 complex to methylate transcription factors and other targets^[^ ^15^, ^16^ ^]^ to mediate various activities. Mechanistically, protein phosphorylation plays an important role in the EZH2 functional switch.

Protein phosphorylation has emerged as one of the most important posttranslational mechanisms^[^ ^17^, ^18^ ^]^ as it regulates the functions of more than 33% of all eukaryote proteins, which consist of transcription factors, cell cycle regulators, chromatin modifiers, and many others implicated in virtually all biological processes.^[^ ^19^ ^]^ It is well established that AKT kinase‐mediated phosphorylation of EZH2 at S21 can suppress the trimethylation of H3K27 by EZH2 in different types of cells.^[^ ^20^, ^21^, ^22^ ^]^ However, the protein phosphatase responsible for reversing the above process remains unknown.

The vertebrate lens has a distinct polarity and elegant structure, responsible for proper light refraction and focus.^[^ ^23^ ^]^ Any change in the normal structure derived from genetic mutation or stress insult causes lens opacification, a condition called cataract.^[^ ^24^ ^]^ Anterior subcapsular cataract (ASC) is caused by abnormal accumulation of lens epithelial cells followed by epithelial–mesenchymal transition (EMT).^[^ ^25^, ^26^ ^]^ In the present study, we demonstrate that EZH2 is highly expressed in ocular lenses and that the AKT‐EZH2 axis is important in TGF*β*‐induced EMT. More importantly, using mass spectrometry, we identified that MYPT1‐ PP1 (protein phosphatase 1) can dephosphorylate EZH2 at S21 and thus modulates its functions. MYPT1 knockout accelerates EMT, and expression of the EZH2‐S21A mutant suppresses EMT by controlling multiple families of genes. Furthermore, the phosphorylation status and gene expression modulation of EZH2 are implicated in the control of ASC in human and mouse eyes. Together, our results identified the specific phosphatase for EZH2 S21 and revealed its novel function in ASC. Mechanistically, EZH2 regulates several families of genes implicated in the control of EMT. These results provide important novel information on EZH2 functions and regulation.

## 2 Results

### 2.1 The Methyltransferase EZH2 Is Highly Expressed in Lens Epithelial Cells, and Its Expression and S21 Phosphorylation Are Enhanced with EMT in Human and Mouse ASC

**Figure 1:**
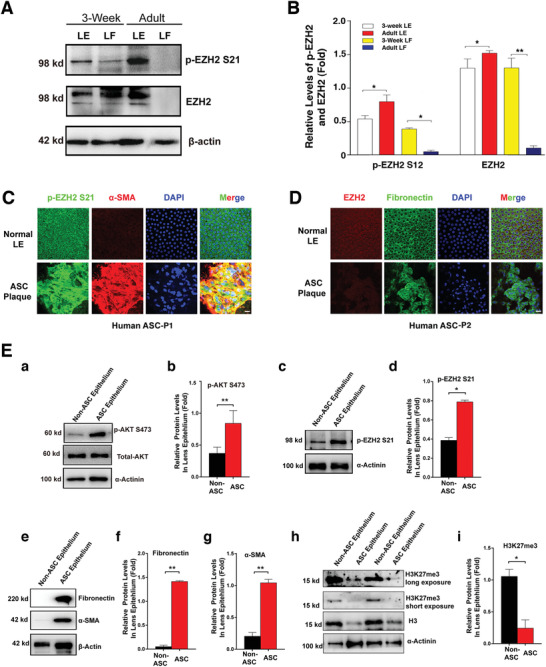
EZH2 phosphorylation at S21 plays a role in lens development and lens fibrotic pathology. A) Western blot analysis of phosphorylated EZH2 at S21 (p‐EZH2 S21) and total EZH2 in lens epithelial and lens fiber cells from 3‐week old and adult mice. B) Quantification of EZH2 and p‐EZH2 S21 expression levels in (A). LE, lens epithelial cells; LF, lens fiber cells. Error bars represent the standard deviation of the mean (*n* = 3). Statistical analysis: one‐way ANOVA, ** *p* < 0.01, * *p* < 0.05. C,D) Immunofluorescence images of lens capsular epithelium from non‐ASC human lenses and ASC patients. C) Immunofluorescence staining for p‐EZH2 S21 (green), *α*‐SMA (red) and lens epithelial cell nuclei (blue). D) Immunofluorescence staining of EZH2 (red), fibronectin (green), and lens epithelial cell nuclei (blue). Note that in (C) through (F),ASC plaques with distorted nuclei were easily detected. Scale bar, 20 µm. E) Western blot analysis of the protein expression levels in lens capsular epithelium from non‐ASC human lenses and ASC patients. E‐a) Western blot analysis of the protein levels of p‐AKTS473. E‐b) Quantification of the protein expression levels of p‐AKTS473 in (E‐a). E‐c) Western blot analysis of the protein levels of p‐EZH2 S21. E‐d) Quantification of the protein expression levels of p‐EZH2 S21 in (E‐c). E‐e) Western blot analysis of the protein levels of Fibronectin and *α*‐SMA. E‐f,g) Quantification of the protein expression levels of fibronectin and *α*‐SMA in (E‐e). E‐h) Western blot analysis of the protein levels of H3K27me3. E‐i) Quantification of the protein expression levels of H3K27me3 in (E‐h). Error bars represent the standard deviation of the mean (*n* = 3). Statistical analysis: Two‐tailed Student's t test; ** *p* < 0.01, * *p* < 0.05.

To understand the physiological role of EZH2 in the ocular lens, we first examined the expression and S21 phosphorylation status of EZH2 in mouse lens epithelial and fiber cells during postnatal developmental stages. Western blot (WB) analysis showed that EZH2 was highly expressed in the epithelial cells of both 3‐week old and adult mouse lenses (**Figure** **1**A,B). In 3‐week old mouse lenses, the EZH2 level in the fiber cells was similar to that in the epithelial cells. In the fiber cells of adult mouse lenses, however, EZH2 was barely detectable (Figure 1A,B). In contrast to the EZH2 expression pattern, in adult mouse lens, the p‐EZH2 level at S21 was higher in LECs compared to 3‐week old mice (Figure 1A,B). In the fiber cells, however, p‐EZH2 S21 was only detected in the 3 week mouse lenses (Figure 1A,B).

Whether EZH2 is implicated in EMT regulation during lens pathogenesis remains to be investigated. The presence of high levels of EZH2 expression in normal mouse lenses (Figure 1A,B) suggests the possibility that EZH2 may be important in lens EMT during ASC development. To test this possibility, we conducted immunocytochemistry and WB analysis on ASC plaques from human patients with normal lens epithelia as control. As shown in Figure 1C–E, AKT activity (S473 phosphorylation) and EZH2 phosphorylation at S21 (p‐EZH2 S21) are much enhanced in ASC plaques than in normal control lens epithelia. The upregulated AKT activity and EZH2 S21 phosphorylation are associated with the downregulated level of H3K27me3. The lower EZH2 activity (as indicated by higher EZH2‐S21 phosphorylation and lower level of H3K27me3) is consistent with increased expression of the EMT markers, fibronectin, and *α*‐smooth muscle actin (*α*‐SMA) (Figure 1E). Similar results have been obtained in all the ASC plaques of five human patients examined (Figure S1 and Table S1, Supporting Information) and also in mouse ASC models (Figure S2, Supporting Information).

### 2.2 TGF*β* Activates AKT‐EZH2‐H3K27Me3 Signaling Axis to Mediate EMT in HLE Cells

To confirm TGF*β*‐induced EMT in HLE cells, we first established cellular EMT models with the human lens epithelial cell line HLE under treatment with the potent EMT inducer TGF*β* for 48 h. Analysis of the expression of EMT marker genes with quantitative real‐time polymerase chain reaction (qRT‐PCR), western blot, and immunofluorescence revealed that TGF*β* induced a 17‐fold increase in FN1 mRNA levels (Figure S3A‐a, Supporting Information). At the protein level, TGF*β* treatment significantly enhanced the expression levels of FN and, to a lesser degree, *α*‐SMA (Figure S3A‐b,c, Supporting Information). Immunofluorescence (IF) staining confirmed the increased expression of both fibronectin and *α*‐SMA in TGF*β*‐treated HLE cells (Figure S3A‐d,e, Supporting Information). Together, these results confirmed that TGF*β* induces EMT in HLE cells as previously reported.^[^ ^27^, ^28^, ^29^, ^30^, ^31^, ^32^, ^33^, ^34^, ^35^, ^36^, ^37^, ^38^, ^39^, ^40^, ^41^, ^42^, ^43^, ^44^, ^45^, ^46^, ^47^, ^48^, ^49^, ^50^, ^51^, ^52^ ^]^ Moreover, our results demonstrated that TGF*β* also suppressed a panel of epithelial cell markers (Figure S3B, Supporting Information), and upregulates numerous other EMT markers including EMT transcription factors such as SNAI1 and SNAI2 and different members of the collagen and extracellular matrix (ECM) family proteins (Figure S3C–E, Supporting Information).

To determine whether AKT can also phosphorylate EZH2 at S21 to modulate EMT in lens cells, we treated HLE cells with a PI3K inhibitor to block AKT activation. As shown in Figure S4A–D (Supporting Information), with PI3K inhibitor (LY294002) treatment to block AKT activation, we observed hardly detectable AKT activity (S473 phosphorylation), attenuated EZH2 phosphorylation at S21 and enhanced global H3K27Me3 levels (lane 3 & 4 of Figure S4A, Supporting Information). AKT phosphorylation of EZH2 was further confirmed by IP analysis (Figure S5A, Supporting Information). As a result, TGF*β*‐induced expression of FN1, a marker of EMT, at both the mRNA and protein levels was completely abolished in HLE cells (Figures S4E–H and S5B, Supporting Information). IF staining further confirmed the above results (Figure S4I, Supporting Information). Together, these results suggest that TGF*β* activates AKT‐EZH2‐H3K27Me3 signaling axis to mediate epithelial–mesenchymal transition (EMT) in HLE cells

To further confirm that AKT kinase phosphorylates EZH2 to modulate TGF*β*‐induced EMT in HLE cells, we treated HLE cells with A‐674563^[^ ^53^ ^]^ and CCT128930,^[^ ^54^ ^]^ selective ATP‐competitive inhibitors for the kinases AKT1 and AKT2, respectively. Compared with mock treatment, AKT1 inhibitor (A‐674563) treatment almost abolished the TGF*β*‐induced changes in AKT1 activity (as reflected by GSK3*β* S9 phosphorylation), EZH2 S21 phosphorylation, and H3K27Me3 levels in HLE cells (Figure S6A–E, Supporting Information). As a result of A‐674563 action, the TGF*β*‐induced expression of the EMT marker genes encoding both fibronectin and *α*‐SMA was also significantly inhibited (Figure S6F–J, Supporting Information). IF staining also revealed that A‐674563 inhibited the cytoplasmic and extracellular localization of fibronectin induced by TGF*β* stimuli, leading to the distribution of fibronectin close to nuclei (Figure S6K, Supporting Information). Moreover, the AKT1 inhibitor, A‐674563 impaired injury‐induced formation of ASC in mouse lens (Figure S7, Supporting Information). The administration of an AKT2 inhibitor (CCT128930) yielded very similar results (data not shown), implying that both AKT1 and AKT2 suppress EZH2 activity to modulate TGF*β*‐induced EMT in HLE cells.

**Figure 2:**
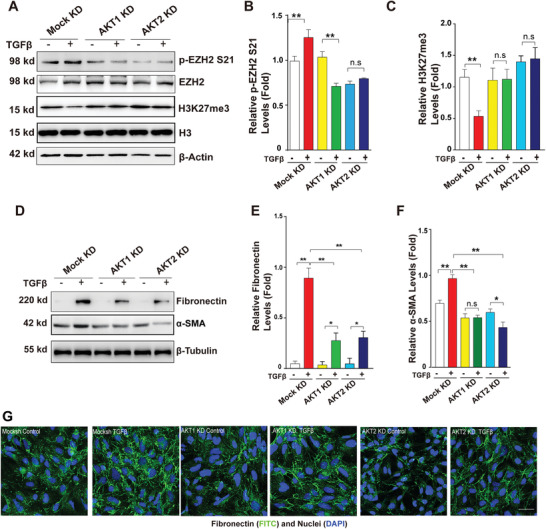
AKT knockdown leads to decreased phosphorylation of EZH2 at S21 and activation of mesenchymal genes. Cultured Mock KD, AKT1 KD, and AKT2 KD‐HLE cells were treated with 20 ng mL^–1^ TGF*β* for 48 h. A. Western blot analysis of the protein levels of p‐EZH2 S21 and H3K27me3. B,C) Quantification of p‐EZH2 S21 and H3K27me3 protein levels. D) Western blot analysis of the protein levels of fibronectin and *α*‐SMA. E,F) Quantification of Fibronectin and *α*‐SMA protein expression levels. G) Immunofluorescence staining of the proteins of Fibronectin in Mock KD, AKT1 KD and AKT2 KD‐HLE cells with indicated treatment. Scale bar, 50 µm. In (B,C,E,F), error bars represent the standard deviation of the mean (*n* = 3). Statistical analysis: Two‐way ANOVA followed by Tukey's correction; ** *p* < 0.01, * *p* < 0.05. n.s, not significant.

To further confirm the importance of AKT1 and AKT2 in regulating EZH2 activity to modulate TGF*β*‐induced EMT in HLE cells, we compared EZH2 phosphorylation at S21, H3K27Me3 levels, and EMT marker gene expression in cell lines with AKT1 and AKT2 KD. The knockdown of AKT1 or AKT2 was confirmed with qRT‐PCR and WB (Figure S8A–D, Supporting Information). Knockdown of either AKT1 or AKT2 failed to upregulate EZH2 phosphorylation at S21 and downregulate H3K27me3 as the Mock KD cells did under TGF*β* treatment (**Figure** **2**A–C). Moreover, the activation of EMT marker genes induced by TGF*β* was almost abolished (Figure 2D–F, and Figure S8E, Supporting Information). IF staining of fibronectin further confirmed the western blot results (Figure 2G). Together, these results demonstrated that both AKT1 and AKT2 regulate EZH2 S21 phosphorylation to modulate TGF*β*‐induced EMT in lens epithelial cells. However, TGF*β*‐induced EZH2 phosphorylation at other sites such as T487 had much less effect on epithelial‐mesenchymal transition in HLE cells. As shown in Figure S9 (Supporting Information), inhibition of T487 phosphorylation by CDK1 knockdown did not affect TGF*β*‐induced expression of fibronectin.

As a comparison, we also examined if epidermal growth factor (EGF) would have similar effects as TGF*β*. As shown in Figure S10 (Supporting Information), although EGF can also activate AKT to increase EZH2 phosphorylation at S21 and suppress expression of epithelial marker genes, it has no effects on the expression of major mesenchymal genes examined.

### 2.3 MYPT1‐PP1 Dephosphorylates EZH2 at S21 to Activate Its Methyltransferase Function

**Figure 3:**
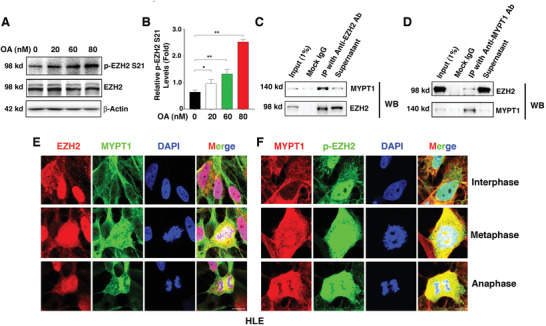
MYPT1 interacts with EZH2 in human lens epithelial cells. A) Western blot analysis of total EZH2 and p‐EZH2 S21 in HLE cells treated with different concentrations of okadaic acid (OA). B) Quantification of p‐EZH2 S21 protein expression levels in the cells shown in (A). Error bars represent the standard deviation of the mean (*n* = 3). Statistical analysis: One‐way ANOVA; ** *p* < 0.01, * *p* < 0.05. C,D) Coimmunoprecipitation (Co‐IP)‐linked western blot analysis of EZH2 and MYPT1 in HLE cells. IgG represents a control antibody used for IP experiments. E) Immunofluorescence staining of EZH2 and MYPT1 in HLE cells at different cell cycle phases. F) Immunofluorescence staining of p‐EZH2 S21 and MYPT1 in HLE cells at different cell cycle phases. Scale bar, 20 µm.

To identify the protein phosphatase that dephosphorylates EZH2 at S21, we first treated HLE cells with okadaic acid (OA), a potent inhibitor of both PP1 and PP2A, which account for 95% of the serine/threonine phosphatase activity in eukaryotes.^[^ ^17^, ^55^ ^]^ As shown in **Figure** **3**A, 20 × 10^‐9^ m OA moderately enhanced the phosphorylation of EZH2 at S21, while 60 × 10^‐9^ and 80 × 10^‐9^ m OA induced a dose‐dependent increase in the p‐EZH2 S21 level. These results suggested that PP1 and PP2A may be responsible for EZH2 dephosphorylation at the S21 residue.

To further clarify the major protein phosphatase responsible for de‐phosphorylating EZH2 at S21, we conducted coimmunoprecipitation‐linked mass spectrometry (co‐IP MS) analysis (Figure S11, Supporting Information). Endogenous EZH2 and bound proteins from HLE cells were immunoprecipitated, gel‐extracted, trypsin‐digested, and then identified by liquid chromatography tandem mass spectrometry (LC‐MS/MS) analysis. Protein identification was performed with MASCOT software by searching the UniProt *Homo sapiens* database. At a significance threshold of 0.05, 340 unique proteins were identified in the anti‐EZH2 immunoprecipitates. Then, screening with peptide‐spectrum matches no less than 2 was applied to identify 285 high‐confidence candidates (Table S2, Supporting Information). Among these proteins were the immunoprecipitation bait protein EZH2 and the canonical PRC2 components SUZ12, EED, and RBBP4, indicating the reliability and accuracy of the mass spectrometry analysis. Of particular interest, the PP1 regulatory subunit PPP1R12A (also termed as MYPT1), was also present. This result demonstrated that PP1 is the major phosphatase dephosphorylating EZH2 at S21. We next confirmed the interaction between EZH2 and MYPT1 using reciprocal coimmunoprecipitation (co‐IP) followed by WB analysis in HLE cells (Figure 3C, D) and in an immortalized rabbit lens epithelial cell line, N/N1003A (Figure S12A,B, Supporting Information). Using IF, we confirmed that EZH2 and MYPT1 interact at different phases of the cell cycle in HLE cells (Figure 3E,F), and similar results were obtained with N/N1003A cells (Figure S12C,D, Supporting Information). Together, these results demonstrated that EZH2‐S21 can be dephosphorylated by PP1.

### 2.4 Knockout of MYPT1 Leads to Accelerated EMT in HLE Cells

**Figure 4:**
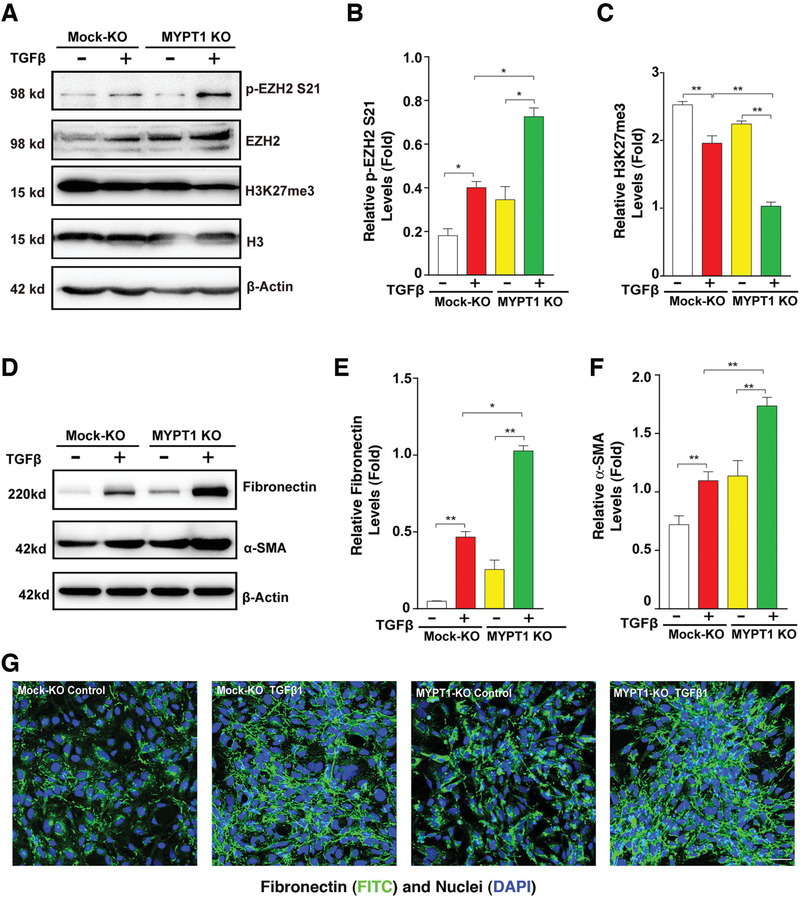
MYPT1 knockout led to accelerated EMT in human lens epithelial cells. Mock KO and MYPT1‐KO HLE cells were treated with 20 ng mL^–1^ TGF*β* for 48 h. A. Western blot analysis of the protein levels of p‐EZH2 S21 and H3K27me3. B,C) Quantification of p‐EZH2 S21 and H3K27me3 protein levels in the cells shown in (A). D) Western blot analysis of the protein levels of Fibronectin and *α*‐SMA. E,F) Quantification of the protein expression levels of fibronectin and *α*‐SMA in the cells shown in (D). G) Immunofluorescence staining of fibronectin. Scale bar, 50 µm. In (B,C,E,F), Error bars represent the standard deviation of the mean (*n* = 3). Statistical analysis: Two‐way ANOVA followed by Tukey's correction; ** *p* < 0.01, * *p* < 0.05.

To confirm that MYPT1‐PP1 is indeed the phosphatase that dephosphorylates EZH2, we next used CRISPR/Cas9 technology to knockout MYPT1 in HLE cells with a guide RNA (gRNA) targeting exon 2, and the results were confirmed with WB analysis and DNA sequencing (Figure S13A,B, Supporting Information). MYPT1 KO enhanced the phosphorylation of EZH2 at S21 and decreased the level of H3K27Me3 in TGF*β*‐treated HLE cells (**Figure** **4**A–C). As a result of the change in EZH2 S21 phosphorylation and H3K27Me3 levels, TGF*β*‐induced EMT was notably enhanced, as reflected by the upregulated expression of fibronectin and *α*‐SMA proteins (Figure 4D–F). This result was further confirmed with IF staining (Figure 4G). To further confirm the EMT control by EZH2 and MYPT1 in lens epithelial cells, we conducted *mypt1* and *ezh2* gene silencing with morpholino oligos in zebrafish (Figure S14A,B, Supporting Information). 99.1% *mypt1* morphant larvae demonstrated severe cardiac edema, curved body axis, and small eyes (Figure S14C,D, Supporting Information), while 56% *ezh2* morphant showed severe cardiac edema, and small eyes, and 35% showed mild cardiac edema and swim‐bladder deficiency (Figure S14C,D, Supporting Information). Analysis of temporal mRNA expression for EMT marker genes in the ocular eye tissues showed that, compared to the wild type fish injected with the control NC Morpholino, *FN1a, LAMC2* and *MMP9* showed significant upregulation in the *mypt1* and *ezh2* morphant larvae on 5 dpf and 6 dpf (Figure S14E,G,H, Supporting Information), *FN1b* displayed consistent upregulation in *mypt1* and *ezh2* morphants from 2 to 6 dpf (Figure S14F, Supporting Information). On the other hand, at 6 dpf, *postnb* showed upregulation in *mypt1* and *ezh2* morphant in the *mypt1* and *ezh2* morphant larvae (Figure S14I, Supporting Information). Taken together, our results identified that MYPT1‐PP1 can dephosphorylate EZH2 to activate its methyltransferase activity and modulate EMT in vitro and in vivo.

### 2.5 Expression of the Exogenous EZH2 S21A Mutant Mimicking Constant Dephosphorylation Suppresses TGF*β*‐Induced EMT in HLE Cells

**Figure 5:**
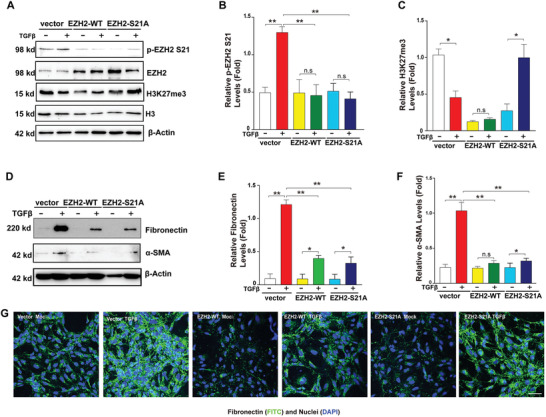
Overexpression of wild‐type EZH2 and the EZH2 S21A mutant in HLE cells attenuated EMT in human lens epithelial cells. HLE cells overexpressing the empty vector, WT‐EZH2 or EZH2 S21A were mock or 20 ng mL^–1^‐TGF*β*‐treated for 48 h. A) Western blot analysis of the protein levels of p‐EZH2 S21 and H3K27me3 in the cells indicated. B,C) Quantification of the p‐EZH2 S21 and H3K27me3 protein levels in (A). D) Western blot analysis of the protein expression levels of fibronectin and *α*‐SMA. E,F) Quantification of Fibronectin and *α*‐SMA protein levels in (D). G) Immunofluorescence staining for fibronectin in the cells indicated. Scale bar, 50 µm. In (B,C,E,F), error bars represent the standard deviation of the mean (*n* = 3). Statistical analysis: Two‐way ANOVA followed by Tukey's correction; ** *p* < 0.01, * *p* < 0.05. n.s, not significant.

To further validate that EZH2 S21 phosphorylation functionally controls EMT in lens epithelial cells, we generated a lentivirus vector expressing either wild‐type EZH2 (EZH2‐WT) or EZH2 with a mutation at S21 (EZH2‐S21A) and subsequently generated stable HLE cell lines expressing EZH2‐WT or EZH2‐S21A. The expression of these proteins was confirmed by WB analysis (Figure S15, Supporting Information). As shown in **Figure** **5**, the expression of exogenous EZH2, either wild type or the S21A mutant, significantly suppressed the TGF*β*‐induced upregulation of fibronectin and *α*‐SMA (Figure 5D–F). IF also confirmed that the expression of WT‐EZH2 or EZH2‐S21A suppressed the TGF*β*‐induced upregulation of fibronectin (Figure 5G). Together, our results further confirm that EZH2 regulates EMT in HLE cells and that EZH2 dephosphorylation by MYPT1‐PP1 modulates its function in regulating EMT in HLE cells.

### 2.6 EZH2 Dephosphorylation Regulates Multiple EMT Genes and Members of Other Gene Families in HLE Cells

To understand the global changes in gene expression regulated by EZH2 dephosphorylation during EMT in lens epithelial cells, we conducted mRNA‐seq analysis in stable cell lines expressing either an empty vector or EZH2 S21A. As shown in Figure S16A (Supporting Information), after TGF*β* treatment, a total of 1697 genes were differentially expressed between the two types of cells, with 688 genes downregulated and 1009 genes upregulated. Among the differentially expressed genes, 23 upregulated genes were included in one of the most enriched Gene Ontology (GO) terms, cell proliferation, and 41 downregulated genes were associated with the GO term extracellular matrix (ECM) proteins (Figure S16B, Supporting Information). Next, we compared the differential gene expression between WT‐EZH2‐ and EZH2‐S21A‐transfected HLE cells. As shown in Figure S16C (Supporting Information), a total of 962 genes were differentially expressed between the two types of cells, with 329 genes downregulated and 633 genes upregulated in TGF*β*‐treated HLE‐EZH2 S21A cells. A total of 31 upregulated genes were included in one of the most enriched GO terms, morphogenesis, and 12 downregulated genes were assigned to the GO term cell adhesion molecules (Figure S16D, Supporting Information). Together, our data demonstrate that changes in EZH2‐S21 phosphorylation status cause global changes in the expression patterns of downstream genes, including both ECM and cell adhesion genes that participate lens EMT control.

**Figure 6:**
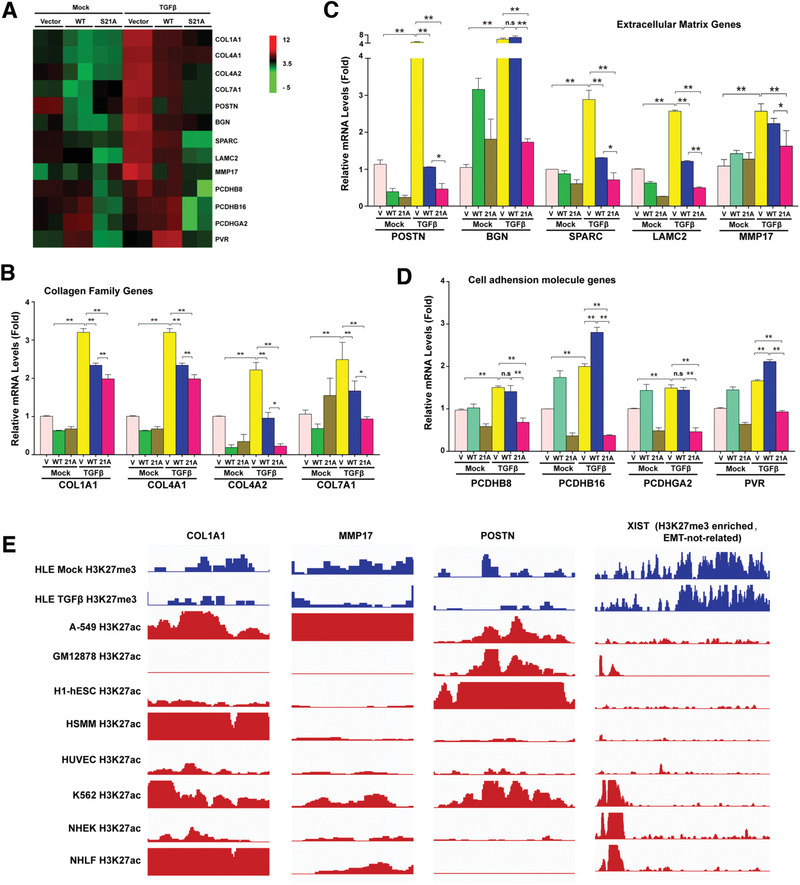
EZH2 S21 phosphorylation controls the expression of multiple families of genes. A) Heatmap of the differential gene expression patterns in the mock or TGF*β*‐treated cells overexpressing the empty vector, WT‐EZH2 or EZH2 S21A. B) qRT‐PCR was used to examine the mRNA levels of collagen family genes COL1A1, COL4A1, COL4A2, and COL7A1. C) qRT‐PCR was used to examine the mRNA levels of extracellular matrix genes POSTN, SPARC, BGN, LAMC2, and MMP17. D) qRT‐PCR was used to examine the mRNA levels of cell adhesion family genes PCDHB8, PCDHB16, PCDHGA2, and PVR. E) ChIP‐seq assay showed H3K27me3 signals on the transcriptional regulatory regions of COL1A1, MMP17, POSTN, and EMT‐unrelated gene XIST in mock or TGF*β* treated‐HLE cells. The transcriptional regulatory regions are annotated by the layered H3K27 acetylation (H3K27ac) tracks of A‐549, GM12878, H1‐hESC, HSMM, HUVEC, K562, NHEK and NHLF cell lines from ENCODE Project. In (B–D), error bars represent the standard deviation of the mean (*n* = 3). Statistical analysis: Two‐way ANOVA followed by Tukey's correction. ** *p* < 0.01, * *p* < 0.05. n.s, not significant.

To further confirm the EMT control by EZH2 dephosphorylation in HLE cells, we established stable cell lines expressing vector, EZH2‐WT or EZH2‐S21A with endogenous EZH2 knocked down (Figure S17, Supporting Information). Using these cell lines, we confirmed 3 families of genes implicated in the control of EMT derived from the above RNAseq analysis and these genes displayed clear differential expression patterns in responding to TGF*β* treatment between EZH2‐WT and EZH2‐S21A expressing cell lines (**Figure** **6**A–D). These include the collagen gene family, extracellular matrix gene family, and the cell adhesion gene family. Among the 3 families of genes, collagen proteins are important positive EMT regulators.^[^ ^26^, ^56^, ^57^ ^]^ We compared four members of the collagen gene family. As shown in Figure 6A,B, Col1A1, Col1A2, Col4A1, and Col7A1 were significantly upregulated under TGF*β* induction in vector‐transfected HLE cells. However, this upregulation was largely suppressed in the WT‐EZH2‐ and EZH2‐S21A‐expressing cells. We further noticed that suppression of the TGF*β*‐induced expression of Col1A1, Col1A2, Col4A1, and Col7A1 was more severe in EZH2‐S21A‐expressing cells than in WT‐EZH2‐expressing cells, indicating the regulatory effect of dephosphorylation on these EMT genes.

Next, we compared the expression levels of members of the extracellular matrix gene family: POSTN, BGN, SPARC, LAMC2, and MMP17.^[^ ^58^ ^]^ As shown in Figure 6C, the mRNA levels of these genes were significantly enhanced in vector‐transfected HLE cells upon TGF*β* treatment. However, their upregulation was abrogated or significantly attenuated in cells transfected with EZH2‐S21A. Overexpression of WT‐EZH2 also significantly suppressed these genes except for BGN, for which TGF*β*‐induced expression was not affected by overexpression of WT‐EZH2. Together, our results indicate that extracellular matrix (ECM) genes are important EZH2 targets and that EZH2 dephosphorylation significantly regulates their expression.

Finally, we examined the mRNA expression levels of four cell adhesion family genes, namely, PCDHB8, PCDHB16, PCDHGA2 and PVR, and found that all four genes were significantly upregulated by TGF*β* treatment in vector‐transfected cells but substantially suppressed in EZH2‐S21A transfected cells (Figure 6D). Interestingly, we noticed that in WT‐EZH2‐transfected cells, the TGF*β*‐induced upregulation of PCDHB16 and PVR was further enhanced (Figure 6D).

To further confirm that EZH2 dephosphorylation mediates the control of the 3 families of genes, we have analyzed the chromatin mark changes on 3 major target genes: Col1A1, MMP17, and POSTN in HLE cells. As shown in Figure 6E, in the TGF*β*‐treated HLE, the repressive chromatin marker H3K27me3 signals is much less than that in the transcriptional regions as reflected by the differential acetylation enrichment (annotated by layered H3K27ac signals) in mock‐treated HLE.

Together, our results demonstrated that EZH2 dephosphorylation causes global changes in different families of genes that mediate the EMT process as well as other cellular processes (Figure S16, Supporting Information).

### 2.7 The EZH2 S21 Phosphorylation‐Regulated POSTN Gene Promotes TGFbeta‐Induced EMT, and its Knockout Significantly Suppresses ASC Development

**Figure 7:**
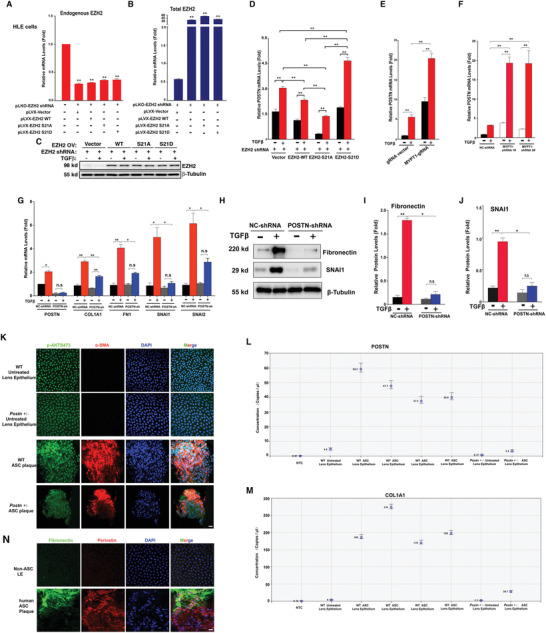
EZH2 S21 phosphorylation activated POSTN gene expression to control mesenchymal gene expression. A) The endogenous EZH2 knockdown is conducted using pLKO lentivirus expressing shRNA targeting EZH2 3′ UTR, and the endogenous EZH2 transcripts are exclusively amplified with 3′ UTR primers. qRT‐PCR was used to examine the endogenous EZH2 mRNA levels of pLKO‐EZH2 shRNA lentivirus infected HLE cells overexpressed with pLVX vector, EZH2 WT, EZH2 S21A, EZH2 S21D. B) qRT‐PCR was used to examine the total EZH2 mRNA levels of endogenous‐EZH2‐knockdown HLE cells overexpressing pLVX vector, EZH2 WT, EZH2 S21A, or EZH2 S21D. C) Western blot analysis of EZH2 proteins in endogenous‐EZH2‐knockdown HLE cells overexpressing pLVX vector, EZH2 WT, EZH2 S21A, and EZH2 S21D subjected to mock or TGF*β* treatment. D) qRT‐PCR was used to examine the mRNA levels of POSTN in endogenous‐EZH2‐knockdown HLE cells overexpressing pLVX vector, EZH2 WT, EZH2 S21A, and EZH2 S21D subjected to mock or TGF*β* treatment. E) qRT‐PCR was used to examine the mRNA levels of POSTN in mock or TGF*β*‐treated HLE cells transfected with gRNA vector or MYPT1 gRNA. F) qRT‐PCR was used to examine the mRNA levels of POSTN in mock or TGF*β*‐treated HLE cells overexpressing control NC shRNA, MYPT1‐shRNA 1# or MYPT1‐shRNA 2#. G) qRT‐PCR was used to examine the mRNA levels of POSTN, COL1A1, FN1, SNAI1, and SNAI2 in the mock or TGF*β*‐treated HLE cells overexpressing control NC shRNA or POSTN‐shRNA. H) Western blot analysis of the protein levels of Fibronectin and SNAI1 in the mock or TGF*β*‐treated HLE cells overexpressing control NC shRNA or POSTN‐shRNA. I,J) Quantification of the fibronectin and SNAI1 protein levels in (H). K) Immunofluorescence staining of p‐AKT S473 (green), *α*‐SMA (red), and lens epithelial cell nuclei (blue) of untreated normal lens epithelium and ASC plaques from WT and Postn ± mouse. Scale bar, 20 µm. L,M) Digital droplet PCR (ddPCR) determined the copy number of POSTN and COL1A1 in the untreated and ASC‐injury treated lens epithelium from WT and Postn ± mouse. Error bars represent the standard error of the mean. N) Immunofluorescence staining for fibronectin (green), periostin (red) and lens epithelial cell nuclei (blue) of lens capsular epithelium from non‐ASC human lenses and ASC patients. Scale bar, 20 µm.

To further demonstrate that the above‐identified EMT genes are indeed subjected to control by EZH2 dephosphorylation and are implicated in regulation of lens EMT and ASC pathogenesis, we analyzed the function of the POSTN gene in HLE cells with the endogenous EZH2 silenced and expression of the exogenous pLVX‐vector plasmid, pLVX‐EZH2, pLVX‐EZH2‐S21A or pLVX‐EZH2‐S21D (**Figure** **7**A–C). As shown in Figure 7D, in EZH2‐S21D expression cells, POSTN is highly expressed. Moreover, MYPT1 knockout with either CRISPR/Cas9 technology (Figure 7E) or shRNA (Figure 7F) greatly induced expression of POSTN mRNA. The enhanced expression of *postn* was also observed in eyes of *mypt1* morphant zebrafish (Figure S14I, Supporting Information). In POSTN knockout human lens epithelial cells, TGF*β*‐induced expression of EMT markers including FN, COL1A1, SNAI1, and SNAI2 mRNA were substantially downregulated (Figure 7G). At the protein level, TGF*β*‐induced fibronectin and SNAI1 were almost blocked in the absence of POSTN gene (Figure 7H–J). To explore the in vivo function, we have knocked out the POSTN gene using CRISPR/Cas9 technology in mouse (Figure S18, Supporting Information). As shown in Figure 7K, induction of ASC in *POSTN* (+/‐) mice was significantly restricted as reflected by the much‐reduced size of ASC plaque and significantly decreased expression COL1A1 and POSTN (Figure 7L,M). Finally, in human ASC plaque, POSTN was highly expressed (Figure 7N).

The periostin protein encoded by *POSTN* gene interacts with transmembrane integrins and thereby activates the AKT kinase and others to regulate EMT among a wide range of cellular processes. POSTN KO causes distinct effect on the AKT‐EZH2‐EMT axis. As shown in Figure S19 (Supporting Information), POSTN knockout abolished AKT activation (Figure S19A,B, Supporting Information), and significantly attenuated expression of the EMT genes, fibronectin, and SNAI1 (Figure S19A, Supporting Information) upon TGF*β* treatment. In addition, transient overexpression of AKT1 in the POSTN knockout human lens epithelial cells can partially rescue the TGF*β*‐induced fibronectin and SNAI1 expression (Figure S19C–E, Supporting Information) Together, POSTN, the EMT marker tightly regulated by MYPT1 and EZH2 phosphorylation plays an important role in lens EMT and ASC development.

### 2.8 Expression Levels of Major EMT Genes Are Significantly Altered in MYPT1 Knockout Cells and during ASC Pathogenesis

**Figure 8:**
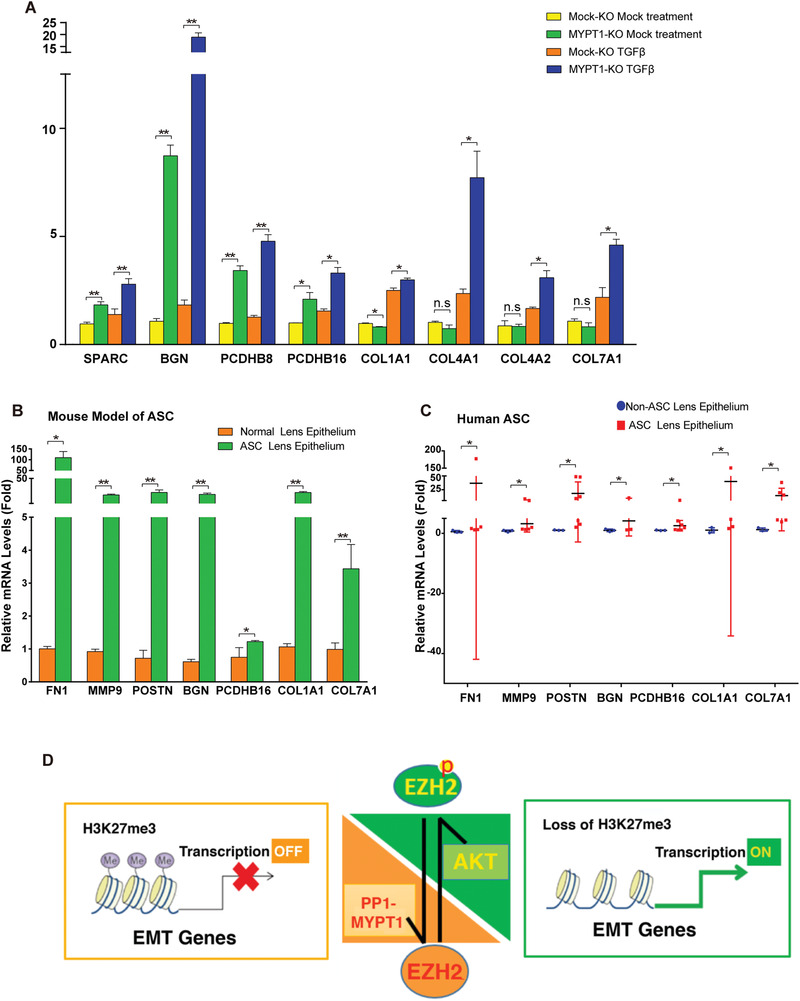
EZH2 S21 phosphorylation‐controlled EMT genes are upregulated during fibrotic disease progression in vivo. A) qRT‐PCR was used to examine the mRNA levels of SPARC, BGN, PCDHB8, PCDHB16, COL1A1, COL4A1, COL4A2, and COL7A1 in the control or TGF*β*‐treated Mock‐KO and MYPT1‐KO HLE cells. Error bars represent the standard deviation of the mean (*n* = 3). Statistical analysis: Two‐way ANOVA followed by Tukey's correction. ** *p* < 0.01, * *p* < 0.05. n.s, not significant. B) qRT‐PCR was used to examine the differential expression levels of FN1, MMP9, POSTN, BGN, PCDHB16, COL1A1 and COL7A1 in the anterior lens capsules of injured‐induced ASC mice at day 7 and the control normal lens capsules. Error bars represent the standard deviation of the mean (*n* = 3). Statistical analysis: Two‐tailed Student's t test; ** *p* < 0.01, * *p* < 0.05. C) qRT‐PCR was used to examine the differential expression levels of FN1, MMP9, POSTN, BGN, PCDHB16, COL1A1, and COL7A1 in the lens epithelium of human ASC patients and non‐ASC patients. Statistical analysis: Kruskal‐Wallis test of independent samples, *p < 0.05. D) A working model showing how the AKT kinase and MYPT1/PP1 holo‐phosphatase modulate EZH2 phosphorylation status to regulate TGF*β*‐induced EMT and ASC in lenses. Upon TGF*β* induction, AKT kinase activation induces the phosphorylation of EZH2 S21, which attenuates the H3K27me3 methyltransferase activity of the PRC2 complex, leading to decreased H3K27me3 levels in the regulatory regions of multiple families of genes controlling EMT to allow their transcriptional activation, thus promoting lens EMT. In contrast, MYPT1/PP1‐mediated dephosphorylation of EZH2 increases EZH2 enzyme activity, enhancing H3K27me3 levels in the regulatory regions of multiple families of genes controlling EMT to shut off their transcription and therefore preventing EMT.

To further identify the EMT‐associated genes that are subjected to direct control by EZH2 dephosphorylation at S21, we examined the mRNA expression levels of SPARC, BGN, PCDHB8, PCDHB16, COL1A1, COL4A1, COL4A2, and COL7A1 in Mock‐KO HLE and MYPT1‐KO HLE cells without or with TGF*β* treatment (**Figure** **8**A). Our results revealed that the expression of SPARC, BGN, PCDHB8, PCDHB16, COL1A1, COL4A1, COL4A2, and COL7A1 was further enhanced more by TGF*β* treatment in the absence of MYPT1 (Figure 8A). More importantly, the expression of 5 genes (POSTN, BGN, PCDHB16, COL1A1, COL7A1) and two other EMT markers (FN1 and MMP9) were significantly enhanced in both the mouse model of ASC (Figure 8B) and in human ASC patients (Figure 8C), suggesting that these EMT genes controlled by EZH2 S21 dephosphorylation significantly contribute to fibrosis during ASC cataractogenesis.

## 3 Discussion

In the present study, we have demonstrated that 1) the EZH2 protein is strongly expressed and phosphorylated in the epithelial cells of developing and adult mouse lenses, but not in the fiber cells of adult lenses (Figure 1A,B); 2) EZH2 phosphorylation is significantly enhanced in ASC plaques, which parallels the enhanced expression of both fibronectin and *α*‐SMA, markers of EMT, as well as newly identified lens EMT genes (POSTN, BGN and PCDHB16) (Figures 1 and 8; Figures S1, S2, and S14I, Supporting Information); 3) the enhanced EZH2 phosphorylation at S21 in ASC is derived from enhanced AKT activity (Figure 1, and Figures S1 and S2, Supporting Information), and inhibition or knockout of AKT1 or AKT2 attenuate EZH2 phosphorylation at S21, enhances H3K27Me3 levels, leading to decreased expression of both fibronectin and *α*‐SMA and prevention of ASC development (Figure 2, and Figures S4, S6, and S7, Supporting Information); 4) mass spectrometry, Co‐IP and coimmunofluorescence analyses demonstrated that MYPT1/PP1 acts as a specific phosphatase that dephosphorylates EZH2 at S21; 5) RNA‐seq analysis demonstrated that EZH2 dephosphorylation causes global changes in the expression patterns of more than 960 genes, among which nearly two dozen are implicated in the control of EMT; 6) changes in EZH2 activity through EZH2‐S21A overexpression/silence or MYPT1 silence greatly altered the expression levels of nearly two dozen EMT genes, and finally, changes in EZH2 S21 dephosphorylation‐controlled EMT genes especially, POSTN gene, contributed to the pathogenesis of ASC. Thus, our results not only identified the specific phosphatase responsible for EZH2 dephosphorylation at S21 but also revealed that EZH2 S21 dephosphorylation plays an important role in ocular pathogenesis (Figure 8D).

### 3.1 MYPT1/PP1 Is a Major Phosphatase That Dephosphorylates EZH2 at S21

EZH2 is an multifucntional epigenetical factor and regulates various physiological processes including cell proliferation, differentiation, apoptosis, and inflammatory response.^[^ ^2^, ^3^, ^6^, ^11^ ^]^ In Kras‐driven nonsmall‐cell lung cancer cells, EZH2 inhibition by GSK126 seems to restrict cell proliferation and promote an inflammatory response mediated by NF‐*κ*B.^[^ ^2^ ^]^ In colorectal cancer‐initiating cells, EZH2 helps to maintain the stem cell‐like property of these cells, and inhibition of EZH2 results in the de‐repression of the hedgehog pathway, decreased self‐renewal, and increased chemotherapy sensitivity in vivo.^[^ ^3^ ^]^ In neuroblastoma with X‐linked alpha‐thalassemia/mental retardation (*ATRX*), inhibition of EZH2 promotes derepression of neuronal genes, causing cell death.^[^ ^6^ ^]^

In addition to its role as the catalytic subunit in the PRC2 complex, EZH2 is also capable of acting as a nonhistone methyltransferase independent of the PRC2 complex to methylate transcription factors and other targets.^[^ ^15^, ^16^ ^]^ This functional switch of EZH2 has been attributed to phosphorylation at the S21 residue. Cha et al.^[^ ^20^ ^]^ demonstrated that AKT is the specific kinase that phosphorylates EZH2 at S21 to dissociate its substrates from the PRC2 complex and thus suppress its methyltransferase activity. The maintenance of EZH2 methyltransferase activity is further enhanced through T350 and T487 phosphorylation by cyclin‐dependent kinase 1 and 2 (CDK1/2).^[^ ^21^ ^]^ Our results reveal that inhibition of T487 phosphorylation by CDK1 silence does not interfere its function in mediating EMT control induced by TGF*β* (Figure S9F–H, Supporting Information). Furthermore, although other growth factors such as EGF can activate EZH2 phosphorylation at S21, it does not induce expression of major lens EMT genes (Figure S10, Supporting Information). In addition, EZH2 can be phosphorylated at multiple serine/threonine sites between residues 200 and 500 by a panel of kinases, including CDK1/2,^[^ ^59^, ^60^ ^]^ MELK,^[^ ^61^ ^]^ PKA,^[^ ^62^ ^]^ AMPK,^[^ ^63^ ^]^ p38,^[^ ^64^ ^]^ and JAK3.^[^ ^65^ ^]^ Phosphorylation at these residues largely controls the stability of EZH2 by blocking the access to ubiquitination ligases. In contrast to our understanding of EZH2 phosphorylation, EZH2 dephosphorylation remains largely unknown. In the present study, we investigated the phosphatase responsible for EZH2 dephosphorylation at the S21 site. First, treatment of human lens epithelial cells with okadaic acid revealed that inhibition of PP‐1 and PP‐2A leads to EZH2 S21 hyperphosphorylation (Figure 3A,B). Subsequently, mass spectrometry analysis (Figure S11, Supporting Information) of the immunoprecipitated protein complex by anti‐EZH2 antibody showed that MYPT1, a PP1 regulatory subunit, exists in the complex with relatively high affinity. The interaction between EZH2 and MYPT1 was further confirmed by coimmunoprecipitation‐linked western blot analysis in both human and rabbit lens epithelial cells (Figure 3C,D, and Figure S12A,B, Supporting Information). Immunofluorescence staining further demonstrated that EZH2 interacts with MYPT1 at both interphase and the dividing cycles in the above cell lines (Figure 3E,F and Figure S12C,D, Supporting Information). Finally, knockout of MYPT1 led to enhanced EZH2 S21 phosphorylation (Figure 4A). Together, our data demonstrate for the first time that MYPT/PP‐1 is the specific phosphatase that dephosphorylates EZH2 at S21.

### 3.2 AKT‐EZH2‐H3K27Me3 Signaling Axis Acts as a Novel Regulatory Mechanism of EMT in LENS

EMT plays an important role during embryonic development, and moreover, it is one of the most prominent biological activities during the progression of various diseases, including fibrosis and tumor metastasis.^[^ ^25^, ^26^, ^27^, ^28^, ^29^, ^30^, ^31^, ^32^, ^33^, ^34^, ^35^, ^36^, ^37^, ^38^, ^39^, ^40^, ^41^, ^42^, ^43^, ^44^, ^45^, ^46^, ^47^, ^48^, ^49^, ^50^, ^51^, ^52^, ^58^, ^59^, ^60^, ^61^, ^62^, ^63^, ^64^, ^65^, ^66^, ^67^, ^68^ ^]^ Mechanistically, EMT is characteristic of reprogramming the gene expression profile, which involves prominent chromatin modifications.^[^ ^1^ ^]^ It has been well established that different signaling pathways mediate EMT in the ocular lens.^[^ ^27^ ^–‐^ ^52^ ^]^ The TGF*β*‐regulated Smad pathway plays a fundamental role.^[^ ^25^, ^26^, ^28^, ^29^, ^30^, ^33^, ^35^, ^42^, ^43^, ^44^ ^]^ During the TGF*β*‐induced EMT process, activation of the ERK1/2 signaling pathway is necessary to initiate EMT activity.^[^ ^69^ ^]^ Modulators of the ERK1/2 pathway, including sprouty,^[^ ^38^, ^43^ ^]^ Scrib,^[^ ^39^ ^]^ and BMP‐7,^[^ ^44^ ^]^ negatively regulate this process. Other signaling pathways mediated by Wnt/*β*‐catenin,^[^ ^46^ ^]^ integrins,^[^ ^31^, ^40^ ^]^ Jagg‐1/Notch,^[^ ^45^ ^]^ and Src kinase^[^ ^32^ ^]^ can also mediate TGF*β* signaling to modulate the EMT process during secondary cataract formation. In addition to these signaling pathways, our previous studies have shown that AKT kinase is abundant and highly activated during mouse lens development.^[^ ^70^, ^71^ ^]^ Earlier studies from others have demonstrated that PI3K/AKT activation participates in TGF*β*‐induced EMT of cultured lens epithelial cells.^[^ ^34^ ^]^ However, the detailed mechanism by which AKT kinase regulates EMT and controls its related gene expression remains elusive. Increasing evidence suggests that AKT kinase converges with cellular signal transduction and chromatin modification dynamics.,^[^ ^72^ ^]^ In this study, we showed that EZH2 is highly expressed and phosphorylated at S21 in postnatal mouse lens tissue (Figure 1A,B), implying its functions during lens differentiation and possible involvement during lens fibrotic pathogenesis. Indeed, several lines of evidence indicate that the AKT‐EZH2‐H3K27Me3 signaling axis activated by TGF*β* plays important roles in lens EMT and cataractogenesis of ASC. First, inhibition of AKT by selective inhibitors (Figures S4–S7, Supporting Information) or knockdown of AKT kinases (Figure 2) not only attenuates EZH2 S21 phosphorylation but also decreases the expression level of EMT genes, including *α*‐SMA and fibronectin; Second, in injury‐induced ASC mouse model, AKT S473 and EZH2 S21 phosphorylation are greatly upregulated (Figure S2, Supporting Information). As a result of upregulated phosphorylation of EZH2 S21, the level of H2K27me3 was downregulated and the expression levels of fibronectin and *α*‐SMA were significantly upregulated in lens epithelium (Figure S2, Supporting Information). Consistent with these results in the injury‐induced ASC mouse model, we also observed that in the lens epithelium of human ASC patients, AKT S473 and EZH2 S21 phosphorylation are significantly upregulated (Figure 1C–E and Figure S1, Supporting Information), and the expression level of H2K27me3 was downregulated but the expression levels of fibronectin and *α*‐SMA was greatly upregulated (Figure 1C–E, and Figure S1, Supporting Information). Finally, knockout or silence of the protein phosphatase 1 regulatory subunit MYPT1 in both cell line and zebrafish, in contrast led to enhanced EZH2 S21 phosphorylation and EMT gene activation (Figures 4 and 8A, and Figure S14, Supporting Information), and also promoted POSTN‐mediated ASC development in mouse (Figure 7). Together, these results demonstrate that the AKT kinase and PP1/MYPT1 phosphatase control the activation of EMT genes in the TGF*β*‐induced response of HLE cells and zebrafish through the modulation of the epigenetic methyltransferase activity of EZH2. In summary, the AKT‐EZH2‐H3K27Me3 signaling axis plays an essential role during TGF*β*‐induced EMT in ASC (Figure 8D).

In addition to histone H3 methyltransferase activity, EZH2 can act as a transcription coactivator via interaction with transcription factors including androgen receptor^[^ ^73^ ^]^ and TCF1,^[^ ^74^ ^]^ and mediate methylation of nonhistone substrates including STAT3.^[^ ^15^ ^]^ Currently, we are trying to identify its substrates that might regulate both EMT and other physiological processes in lens.

### 3.3 Dephosphorylation of EZH2 and Other Targets Plays a Fundamental Role in Regulating EMT in Both Lens and Non‐Lens Systems

Protein dephosphorylation at the serine and threonine residues of various proteins, similar to their cognate phosphorylation, has important functions in regulating normal cellular physiology and pathology.^[^ ^17^, ^56^ ^]^ We previously showed that PP‐1 plays an important role in mediating the complete inactivation of AKT kinase by dephosphorylating the T450 residue and thus modulates the control of lens differentiation and apoptosis by AKT.^[^ ^72^ ^][^ ^70^ ^]^ In the present study, we present the first evidence that MYPT1/PP1 can also regulate EZH2 activity by dephosphorylating S21. A recent study by Ferreira et al.^[^ ^75^ ^]^ reported that NIPP1/PP1 can dephosphorylate EZH2 at Thr‐345/347 to modulate its function. Thus, the protein dephosphorylation mediated by PP1 can control EMT by regulating both AKT and EZH2.

As mentioned above, the TGF*β*/Smad signaling pathway plays a fundamental role in lens EMT.^[^ ^25^, ^26^, ^28^, ^29^, ^30^, ^33^, ^35^, ^42^, ^43^, ^44^ ^]^ It has been shown that the dephosphorylation of Smads by PPM1A/PP‐2C*α* can terminate the TGF*β*/Smad signaling pathway,^[^ ^76^ ^]^ thus blocking the EMT process. Furthermore, the tumor suppressor p53 can regulate EMT through the direct control of different microRNAs to regulate downstream target genes.^[^ ^77^ ^]^ We have previously revealed that both PP‐1 and PP‐2A can dephosphorylate p53 at Ser‐15 and Ser‐37 to modulate the transcriptional activity of p53.^[^ ^78^ ^]^ Thus, by regulating major signaling pathways such as the AKT‐EZH2 signaling axis and TGF*β*/Smad pathways, as well as key transcription factors including p53 and others, protein dephosphorylation mediated by PP1, PP2A and other serine/threonine phosphatases plays a fundamental role in controlling EMT in both lens and non‐lens systems.

### 3.4 EZH2 S21 Phosphorylation/Dephosphorylation Regulates Multiple Families of Genes, Contributing to Fibrotic Diseases in the Ocular Lens

Fibrosis occurs due to abnormal damage repair and wound healing.^[^ ^79^, ^80^ ^]^ It is linked to many human diseases, including liver cirrhosis, renal fibrosis, pulmonary fibrosis, epithelial tumors, and fibrotic eye diseases.^[^ ^81^, ^82^, ^83^, ^84^, ^85^ ^]^ The most important characteristic of fibrotic diseases is the epithelial–mesenchymal transition (EMT), in which activated transcription factors including snail 1/2, Twist, ZEB1/2 and others repress expression of E‐cadherin, ZO‐1 and other epithelial marker genes. At the same time, mesenchymal genes, including *α*‐SMA, fibronectin, and others are activated.^[^ ^25^, ^26^, ^81^, ^82^, ^83^, ^84^, ^85^ ^]^

Fibrotic eye diseases occur in major ocular tissues, including the cornea, conjunctiva, lens, retina, optic nerve, and orbit.^[^ ^25^, ^26^, ^84^, ^85^ ^]^ In the ocular lens, fibrosis causes both anterior subcapsular cataract (ASC)^[^ ^28^, ^29^, ^30^ ^]^ and posterior subcapsular cataract (PCO).^[^ ^86^ ^]^ ASC is associated with several conditions, including trauma,^[^ ^87^ ^]^ ocular inflammation,^[^ ^41^, ^88^ ^]^ and iritis.^[^ ^89^ ^]^ At the molecular level, elevated expression of *α*‐SMA was detected in human ASC.^[^ ^41^ ^]^ In addition, collagen proteins I–IV were detected in human ASC using immunohistochemistry.^[^ ^90^ ^]^ In mouse models of ASC, similar results have been reported.^[^ ^28^, ^29^, ^30^ ^]^ In the present study, our results confirmed these early studies (Figures 1 and 6, 7, 8; Figures S1, S2, and S4–S7, Supporting Information). Moreover, we identified a panel of additional genes that are implicated in ASC. Using RNA‐seq, we found 37 upregulated ECM genes in human lens epithelial cells undergoing TGF*β*‐induced EMT (Figure 6 and Figure S16, Supporting Information). Of particular interest, overexpression of EZH2‐S21A abrogated the TGF*β*‐induced global decrease in the transcription repression marker H3K27Me3 and accordingly significantly suppressed TGF*β*‐induced activation of important EMT markers, including collagens, extracellular matrix genes, and cell adhesion family genes (Figures 6 and 8, and Figure S16, Supporting Information). These results implied that EZH2‐H3K27Me3 is an important epigenetic pathway that controls EMT in lens. Its control on the expression of collagens and extracellular matrix genes is closely related to the transition from epithelium to fibroblasts, and the ultimate formation of subcapsular plaques beneath the lens capsule.^[^ ^28^ ^]^ Activation of the cell adhesion genes may also help cell migration and the establishment of the subcapsular plaques. Of course, it will take substantial efforts of different laboratories to elucidate the exact functions of these genes during ASC development. Nevertheless, our effort in the present study also revealed that expression of the well‐defined ECM gene, POSTN is directly controlled by EZH2 S21 dephosphorylation (Figure 7). As both a marker and promoter of EMT, POSTN have been shown to promote progression of several types of cancers.^[^ ^91^ ^]^ In both human and mouse models of ASC, here we observed that POSTN is significantly upregulated (Figures 7 and 8B,C). Moreover, POSTN actively promotes ASC development as demonstrated in POSTN KO mice (Figure 7). The exact functional mechanism of POSTN, like other identified genes will have to wait further investigation. Together, our results demonstrated that EZH2 dephosphorylation mediated by MYPT1/PP1 regulates TGF*β*‐induced expression of a panel of novel genes to regulate ASC pathogenesis.

## 4 Experimental Section

### Chemicals

Various molecular biology reagents were purchased from Invitrogen Life Technologies (Gaithersburg, MD); Stratagene(La Jolla), CA; and Promega Biotech (Madison, WI). All DNA and protein size markers were purchased from Invitrogen Life Technologies (Gaithersburg, MD). The mammalian expression vector was purchased from Clontech (Palo Alto, CA). Various antibodies were obtained from Cell Signaling Technology (Boston, MA); Roche Molecular Biochemicals (Indianapolis, IN); Santa Cruz Biotechnology(Dallas, TX); Abcam Inc(Cambridge, MA) and Abclonal Inc (Wuhan, China). The culture medium and most other chemicals and antibiotics were purchased from Sigma (St. Louis, MO) and Invitrogen Life Technologies(Gaithersburg, MD).

### Animals

The mice used in this study were handled in compliance with the Guide for the Care and Use of Laboratory Animals (National Academy Press). 3 week old and adult mice were obtained from the animal facility of Sun Yat‐sen University. Various ocular tissues including cornea, retina, and lens epithelium, and lens fiber were collected from these mice and used to extract total RNA and proteins.

### Mouse ASC Injury Model

All animal studies conformed to the Association for Research in Vision and Ophthalmology Statement for the Use of Animals in Ophthalmic and Vision Research. Animal care and experimental procedures were carried out in accordance with the approved guidelines of the Ethics Committee in Animal and Human Experimentation of Sun Yat‐sen University (2017‐082A). The injury‐induced ASC mouse model was established as previously described.^[^ ^49^ ^]^ Briefly, 4 to 6 week old C57BL/6J mice underwent general anesthesia with an intraperitoneal injection of pentobarbital sodium (70 mg kg^–1^) and topical anesthesia with dicaine eyedrops. After the topical application of mydriatic eyedrops, a small incision was made in the central anterior capsule of one eye through the cornea with the blade part of a 26‐gauge hypodermic needle. The other eye of the mouse was used as a normal control without any treatment. The depth of injury was approximately 300 µm or one fourth of the length of the blade part of the needle, which has been reported previously to lead to the formation of fibrotic tissue around the capsular wound.^[^ ^49^ ^]^ For AKT1 inhibitor treatment, the inhibitor was injected into the anterior chambers of the injured eyes immediately after injury with a microsyringe (30‐gauge, Hamilton). The mice were randomly divided into two groups to receive 1 µL of 20 × 10^‐6^ m of A‐674563 and an equivalent amount of DMSO as a control. The mice were allowed to heal for 7 d. Then, the mice were sacrificed, and the lenses were harvested for qRT‐PCR, ddPCR, western blot analyses, and whole‐mount staining.

### Human ASC Lens Capsular Samples

Human ASC lens capsular samples were collected from patients who were admitted to our hospital and diagnosed with anterior subcapsular cataract (ASC), and the non‐ASC group refers to nuclear cataract patients with clear lens capsules and without other ocular disease symptoms. Lens capsular samples were collected during intraocular lens implantation surgery. Patient consent and the approval of the Ethics Committee of Zhongshan Ophthalmic Center, Sun Yat‐sen University (Guangzhou, China) (2017KYPJ041) were obtained before the human tissue samples were harvested.

### Immunofluorescent Staining for Lens Anterior Capsule Whole Mount

For whole‐mount lens anterior capsules, injured mice were sacrificed, and eyes were enucleated and immediately transferred to a culture plate filled with PBS. To isolate the lens anterior capsule, the lens was dissected carefully under a dissecting microscope. Immediately the anterior capsules of the lenses were isolated under a dissecting microscope and transferred to 4% paraformaldehyde and allowed to fix for 15 min on ice. Human ASC capsular samples were fixed in 4% paraformaldehyde for 15 min. After fixation, the capsular samples were washed three times with PBS and permeabilized with ice‐cold methanol/acetone (1:1). The permeabilized samples were blocked in normal goat serum for 1 h followed by overnight incubation at 4 °C with primary antibodies against p‐EZH2‐S21 (1:100, Bethyl Laboratories), EZH2 (1: 1:100, Cell Signaling Technology), Fibronectin (1:500, Abcam), p‐AKT S473(1:2 00, Cell Signaling Technology) and *α*‐SMA (1: 1:100, Cell Signaling Technology), Periostin (1:100; Santa Cruz Biotechnology). On the following day, the capsules were washed with PBS three times for 30 min and thereafter incubated with appropriate secondary antibodies for 1 h. After washing with PBS for 30 min, the capsules were incubated with a sufficient volume of DAPI for 10 min to stain the nuclei. Finally, the whole anterior capsule was mounted flat on a microscope slide and covered with a coverslip after adding a drop of anti‐fade mounting medium. The slides were stored at 4 °C in the dark for later examination using a confocal microscope. Images were acquired with a Zeiss LSM confocal laser scanning microscope (CLSM, Carl Zeiss) and analyzed with ZEN2.1 software.

### Cell Culture and Treatment

The human lens epithelial cell line HLE and FHL124, rabbit lens epithelial cell line N/N1003A and human embryonic kidney (HEK)‐293FT cell line were cultured in monolayer in a 37 °C incubator with 5% CO_2_ in Dulbecco's modified Eagle's medium (DMEM) (Sigma) supplemented with 10% fetal bovine serum (FBS) and 50 units mL^–1^ penicillin and streptomycin; 10% rabbit serum was used instead of fetal bovine serum in the culture medium of N/N1003A cells. The HLE‐AKT1 KD, HLE‐AKT2 KD and Mock KD cell lines were established in our previous study.^[^ ^71^ ^]^ Cells were passaged with 0.25% trypsin, seeded in six‐well plates at a density of 1 × 10^5^ cells per well, and treated with 20 ng mL^–1^ TGF*β* (Syd Labs, Sigma), with or without 0.5 × 10^‐6^ m A‐674563, or 20 × 10^‐6^ m LY294002 (Sellek) in DMEM without FBS for 48 h. For okadaic acid treatment, the cells were FBS‐starved for 12 h before the 3 h treatment of okadaic acid.

### Western Blot (WB) Analysis

For total protein extraction, the cells or zebrafish larvae were lysed in RIPA buffer (150 × 10^‐3^ m NaCl, 50 × 10^‐3^ m Tris‐Cl pH 8.0, 1% NP‐40, 1% sodium deoxycholate, 0.1% SDS) with protease inhibitor cocktail and protein phosphatase inhibitor cocktail(Roche). The denatured protein samples were separated by 8–15% sodium dodecyl sulfate‐polyacrylamide gel electrophoresis (SDS‐PAGE) and transferred to nitrocellulose membranes (Bio‐Rad). The membrane was blocked in 5% nonfat milk, incubated with primary antibody at 4 °C overnight, and washed with TBST (10 × 10^‐3^ m Tris pH 8.0, 150 × 10^‐3^ m NaCl, 0.1% Tween‐20) three times. Then, the membrane was incubated with horseradish peroxidase (HRP)‐conjugated secondary antibodies for 1 h. Protein bands were detected with chemiluminescence detection reagents. *β*‐Actin was used as a loading control. Quantification analysis was conducted using ImageJ 1.41 (National Institutes of Health). Statistical analysis was performed using Prism 7 software (GraphPad, La Jolla, CA). The sources and dilutions of antibodies were as follows: mouse anti‐fibronectin (1:100, Developmental Studies Hybridoma Bank, University of Iowa), rabbit anti‐*α*‐SMA (1:2000, Abclonal), rabbit/mouse anti‐EZH2 (1:1000, Abclonal #A11085 and Cell Signaling Technology #5246 & #3147), rabbit anti‐p‐EZH2 Ser21 (1:500, Bethyl Laboratories), rabbit anti‐p‐EZH2 T487 (1:1000, # PA5‐105660, Invitrogen), rat anti‐SNAI1 (1:1000, Cell Signaling Technology#4719), rabbit anti‐SNAI2 (1:1000, Cell Signaling Technology#9585), rabbit anti‐H3K27me3 (1:1000, Cell Signaling Technology #9733, Abclonal A2363), rabbit anti‐H3 (1:1000, Abclonal A2348), rabbit anti‐alpha smooth muscle actin (1:1000, Abclonal A7248), mouse anti‐Flag (1:1000, F1804, Sigma), rabbit anti‐MYPT1 (1:1000, Thermo Fisher Scientific, PA5‐17164), mouse anti‐MYPT1 (1:1000, Santa Cruz, sc‐514261), mouse anti‐AKT1(1:1000, Santa Cruz, sc‐5298), rabbit anti‐AKT2 (1:1000, Cell Signaling Technology#2964), rabbit anti‐pan AKT(1:1000, Cell Signaling Technology#4691), rabbit anti‐pGSK3*β* S9(1:1000, Cell Signaling Technology#9336), rabbit anti‐GSK3*β*(1:1000, Cell Signaling Technology#9315), rabbit anti‐Claudin‐1 (1:1000, Cell Signaling Technology#13255), rabbit anti‐ZO‐2 (1:1000, Cell Signaling Technology#2847), rabbit anti‐N‐cadherin (1:1000, Cell Signaling Technology#4061), rabbit anti‐pAKT S473 (1:1000, Cell Signaling Technology #4060), mouse anti‐CDK1 (1:1000; sc‐54, Santa Cruz Biotechnology), mouse anti‐*β*‐actin (1:5000, proteintech, 66009‐1‐Ig), mouse anti‐*β*‐Tubulin (1:5000, proteintech, 66240‐1‐Ig), rabbit anti‐*α*‐Actinin(1:5000, proteintech, 11313‐2‐AP), horseradish peroxidase (HRP)‐conjugated horse anti‐ rabbit/mouse/rat IgG (1:5000, Cell Signaling Technology).

### Coimmunoprecipitation

Cells were collected on ice and lysed in 0.2% NP‐40 co‐IP buffer (10 × 10^‐3^ m Tris‐Cl, pH 7.4, 150 × 10^‐3^ m NaCl, 0.2% NP‐40, and 10% glycerol) supplemented with protease inhibitors and protein phosphatase inhibitor cocktail, incubated on ice for 15 min, and cleared by centrifugation at 13 200 rpm at 4 °C for 15 min. Total protein lysate (2–5 mg) was precleared with normal rabbit IgG for 2 h at 4 °C, and 1 mL precleared lysate was subjected to immunoprecipitation with EZH2 antibody (Cell Signaling Technology#5246) or MYPT1 antibody (Cell Signaling Technology #8574) as well as protein A/G agarose beads overnight at 4 °C. The immunoprecipitates were washed six times with co‐IP buffer, and then the washing buffer was completely removed from the beads. The beads were boiled in SDS sample buffer, and then the proteins were separated by SDS‐PAGE and transferred to nitrocellulose membranes. The blots were developed with anti‐MYPT1 (1:1000, Santa Cruz, sc‐514261) or with anti‐EZH2 (Cell Signaling Technology #5246 & #3147) antibodies.

### LC‐MS/MS Analysis

LC‐MS/MS (Sciex TripleTOF 5600, Applied Biosystems) was performed by Fitgene Biological Technology Co. Ltd (Fitgene, Guangzhou, China). Protein identification was performed with MASCOT software by searching UniProt *homo sapiens*. Proteins with more than 2 peptide‐spectrum matches were considered as highly‐confident EZH2‐interacting protein candidates in HLE cells.

### RNA Isolation, Real‐Time PCR, and RNA‐Seq Analysis

Total RNA was isolated using TRIzol reagent (Invitrogen) and then processed with an RQ1 RNase‐Free DNase kit (Promega) to eliminate genomic DNA contamination. First‐strand cDNA was synthesized with a First Strand cDNA Synthesis Kit (Fermentas). Real‐time qPCR was performed in triplicate using SYBR Green I Master mix (Vazyme) with a Roche LightCycler 480II real‐time qPCR system. The qRT‐PCR primers are listed in Table S3 (Supporting Information). GAPDH and *β*‐actin were used as internal controls for human and mouse samples, respectively. All primers were synthesized by Sangon Biotech (Shanghai, China) and TsingKe Biotech (Beijing, China). Statistical analysis was performed using Prism 7 software (GraphPad, La Jolla, CA).

RNA‐seq analysis was conducted by Haplox Genomics Center (ShenZhen, China), and sequencing was performed with Illumina NovaSeq6000. For the RNA‐seq analyses, total RNA was extracted from mock or TGF*β*‐treated HLE‐vector cells, HLE EZH2‐WT cells and HLE EZH2‐S21A cells using TRIzol reagent according to the manufacturer's instructions. RNA‐seq library preparation and subsequent sequencing were conducted by the Haplox Genomics Center (ShenZhen, China). Pooled samples of two biological repeats were sequenced on an Illumina NovaSeq6000. The obtained sequence reads were cleaned and mapped to GRCm38/mm10 using Tophat. Gene expression and changes were analyzed using Bowtie2 and RSEM. The relative abundance of mRNAs was normalized and presented as fragments per kilobase of transcript per million mapped reads (FPKM). Hierarchical cluster and scatter plot analyses of gene expression levels were performed using R software (http://www.r‐project.org/). KEGG analysis was carried out by Kobas. Samples harvested from two independent experiments were pooled and used for each RNA‐seq analysis sample.

### Digital Droplet PCR (ddPCR)

Total RNA was isolated using TRIzol reagent (Invitrogen) and then processed with ‐ RQ1 RNase‐Free DNase kit (Promega) to eliminate genomic DNA contamination. First‐strand cDNA was synthesized with a First Strand cDNA Synthesis Kit (Fermentas) using equal amount of RNA. Prior to digital droplet PCR, qRT‐PCR was used to determine the Cp value of mouse *β*‐Actin. Then qRT‐PCR normalized cDNA was amplified using 2× EvaGreen ddPCR Mastermix and thermal cycled at 95 °C for 5 min followed by 40 cycles of 95 °C for 30 s and 58 °C for 1 min. Signal was then stabilized at 4 °C for 5 min followed by inactivation at 90 °C for 5 min. Droplets were then read by a QX200 Droplet Digital PCR System and analyzed with QuantaSoft V1.7 (BioRad).

### ChIP Assay

HLE cells were mock‐treated or treated with 20 ng mL^–1^ TGF*β* for 48 h. Chromatin immunoprecipitation (ChIP) was performed using Simple ChIP Enzymatic Chromatin IP Kit (Cell Signaling Technology#5246). Briefly, 5 ×10^6^ mock or TGF*β*‐treated cells were cross‐linked with 1% formaldehyde at room temperature for 10 min. After micrococcal nuclease digestion, chromatin was sonicated into 150‐900 bp DNA fragments. 10 µg H3K27me3 antibody(Cell Signaling Technology, #9733) were incubated with magnet Protein A/G beads for at least 3 h before incubating with 10 µg chromatin overnight. H3K27me3 ChIP DNA was subjected to ChIP‐seq analysis by Haplox Genomics Center (ShenZhen, China). The identification of ChIP‐seq peaks was performed using MACS 2.0.

### Expression Constructs

cDNA obtained from HLE cells was used as PCR templates to amplify the coding sequences of the human EZH2 gene. AKT1 was cloned into 3× FLAG pCMV‐10 vector at the EcoRI (5′) and BamHI (3′) sites. EZH2 was cloned into the pLVX‐IRES‐Puro vector at the NheI (5′) and BamHI (3′) sites, and the FLAG tag sequence was integrated into the forward primer to generate the FLAG‐EZH2 fusion protein. The pLVX‐IRES‐Puro‐EZH2 S21A mutation construct was obtained using the QuikChange Site‐Directed Mutagenesis Kit according to the manual (Agilent Technologies). The primers used to clone AKT1, EZH2 WT and conduct the mutagenesis of EZH2‐S21A are listed in Table S4 (Supporting Information). All constructs were confirmed by sequencing.

### RNA Interference

For shRNA‐mediated gene knockdown a set of single‐stranded oligonucleotides encoding the EZH2, POSTN, CDK1, MYPT1 target shRNA and its complement were synthesized. EZH2: sense, 5′‐CCGGTATTGCCTTCTCACCAGCTGCCTCGAGGCAGCTGGTGAGAAGGCAATATTTTTTG‐3′; POSTN: sense, 5′‐ CCGGCGAGCCTTGTATGTATGTTATCTCGAGATAACATACATACAAGGCTCGTTTTTG‐3′, CDK1: sense, 5′‐CCGGTGGCTTGGATTTGCTCTCGAACTCGAGTTCGAGAGCAAATCCAAGCCATTTTTG‐3′, MYPT1 #1: sense, 5′‐CCGGTTGCGAACAAGTAGTTCATATCTCGAGATATGAACTACTTGTTCGCAATTTTTG‐3′, MYPT1 #2: sense, 5′‐CCGGGCACTACTACAAAGATTACAACTCGAGTTGTAATCTTTGTAGTAGTGCTTTTTG‐3′.The oligonucleotide sense and antisense pair were annealed and inserted into pLKO lentiviral expression system.

### Lentivirus Infection

The psPAX2 packaging plasmid, pMD2.G envelope plasmid and the pLVX‐IRES‐Puro vector, pLVX‐IRES‐Puro‐EZH2 WT, pLVX‐IRES‐Puro‐EZH2 S21A, pLKO‐NC shRNA, pLKO‐ EZH2 shRNA, pLKO‐POSTN shRNA, pLKO‐CDK1 shRNA, pLKO‐MYPT1 1# shRNA, pLKO‐MYPT1 2# shRNA constructs were used to co‐transfect HEK‐293FT cells. Virus supernatant was collected 48 h posttransfection, filtered through a 0.45 µm polyethersulfone filter. The virus suspension was mixed with 8 µg mL^–1^ polybrene to infect HLE cells. Puromycin screening was conducted 48 h postinfection. Western blot and qRT‐PCR analyses were used to identify gene knockdown and EZH2 WT /S21A overexpression.

### Immunofluorescence Staining

Cells were seeded on Millicell EZ 24‐well glass slides (Millipore). After treatment for the indicated times, the cells were fixed with 4% paraformaldehyde, permeabilized with methanol/acetone (1:1), and blocked with normal goat serum. Then, the sections were incubated with anti‐fibronectin (1:200, Abcam) and anti‐*α*‐SMA (1:200, Abclonal) prepared with goat serum. After washing in PBS, the sections were incubated with fluorescein goat anti‐rabbit IgG or fluorescein goat anti‐mouse IgG (1:200, Cell Signaling Technology). Cell nuclei were stained with 50 ng mL^–1^ 4’,6‐diamidino‐2‐phenylindole (DAPI) for 5 min. Slides were mounted with anti‐fade fluorescent mounting medium (Applygen). Images were acquired by a Zeiss LSM confocal laser scanning microscope (CLSM, Carl Zeiss) and analyzed with ZEN2.1 software.

### CRISPR‐Cas9 Mediated MYPT1 Gene Editing in HLE

The sequence of the MYPT1 gRNA targeting exon 2 was 5′‐ ACCAAGGTGAAGTTCGACGA‐3′. One oligo of this sequence plus caccG (G was used to grant gRNA transcription) at the 5′ end and the other of the complementary sequence plus aaac at 5′ were annealed and ligated into the BbsI‐digested M2 px459 gRNA vector. The MYPT1‐gRNA plasmid was confirmed by DNA sequencing. A 5 µg plasmid was transfected into HLE cells using Lipofectamine 3000. Puromycin screening was used to eliminate the nontransfected cells. The genomic DNA of the MYPT1‐gRNA plasmid‐transfected single‐cell clones was extracted using a genomic DNA extraction kit (TIANGEN Biotech, Beijing, China). The DNA fragments containing the MYPT1‐gRNA editing sites were amplified with specific primers (Table S4, Supporting Information) and subjected to sequencing. Western blot analysis was also were applied to identify the MYPT1‐KO cells.

### CRISPR‐Cas9 Mediated POSTN Gene Editing in Mouse

The POSTN heterogeneous knockout mouse model was generated by coinjection of Cas9 mRNA and short guide RNA (sgRNA) (Figure S17, Supporting Information). In short, superovulated female C57BL/6 mice were mated to male C57BL/6 mice, and embryos were collected from oviducts. Cas9 mRNA (100 ng µL^–1^), two sgRNAs (50 ng µL^–1^) targeting the *Postn* gene locus were coinjected into the pronuclei of one‐cell embryos. The injected embryos were cultured in vitro for two hours, then the survived embryos were transplanted into pseudopregnant mice. At 1 week after birth, genomic DNA from the toes of the newborn F0 mice was extracted for PCR analysis and DNA sequencing.

### MYPT1 and EZH2 Silence with Morpholino Oligoes

Zebrafish larvae and adults were maintained at 26–28.5 °C under a 14/10 h light/dark cycle. Fertilized eggs were collected and maintained in E3 medium in an incubator (at ≈28.5 °C) for 72 h until the larvae hatched. All procedures involving zebrafish were approved by the Ethics Committee of Sun Yat‐sen University and were in accordance with Animal Research: Reporting of in Vivo Experiments (ARRIVE) guidelines.

Morpholino oligoes (MO) were obtained from Gene Tools (Philomath, USA). The mypt1 splicing blocking Morpholino (mypt1‐MO, 5′‐CGTAACGCAACGCTCTTCTTACCTG‐3′), ezh2 translation blocking Morpholinos (ezh2‐MO: 5′‐CCGATTTCCTCCCGGTCAATCCCAT‐3′) and a standard negative control MO (NC MO: 5′‐CCTCTTACCTCAGTTACAATTTATA‐3′) were resuspended in water as a 8 × 10^‐3^ m stock solution and diluted to the appropriate concentrations for microinjections. 1 ng of mypt1‐MO, 8 ng of ezh2‐MO and control MO were injected into zebrafish embryos at one‐cell stage as previously reported^[^ ^92^, ^93^ ^]^ to effectively inhibits mypt1 and ezh2 expression, respectively.

### Statistical Analysis

Quantitative data are presented as the mean±SD and analyzed by one‐way ANOVA or two‐way ANOVA, and multiple comparisons between the groups were corrected using Tukey's post hoc test. Two‐tailed Student's t test was performed when two groups were compared. The Kruskal‐Wallis test of independent samples was used in the case of human patient samples. Statistical significance was set at a level of *P* < 0.05. In the figures, * and ** refer to *P* < 0.05 and *P* < 0.01, respectively. The data shown are the averages and standard deviations of at least three biological replicates. Statistical analysis was performed with GraphPad Prism 7.0 or SPSS software.

## Conflict of Interest

The authors declare no conflict of interest.

## Supporting information

Supporting Information

## Data Availability

The data that support the findings of this study are available from the corresponding author upon reasonable request.

## References

[1] E. Vire , C. Brenner , R. Deplus , L. Blanchon , M. Fraga , C. Didelot , L. Morey , A. Van Eynde , D. Bernard , J. M. Vanderwinden , M. Bollen , M. Esteller , L. Di Croce , Y. de Launoit , F. Fuks , Nature 2006, 439, 871. 16357870 10.1038/nature04431

[2] M. Serresi , B. Siteur , D. Hulsman , C. Company , M. J. Schmitt , C. Lieftink , B. Morris , M. Cesaroni , N. Proost , R. L. Beijersbergen , M. van Lohuizen , G. Gargiulo , J. Exp. Med. 2018, 215, 3115. 30487290 10.1084/jem.20180801 PMC6279402

[3] E. Lima‐Fernandes , A. Murison , S. M. T. Da , Y. Wang , A. Ma , C. Leung , G. M. Luciani , J. Haynes , A. Pollett , C. Zeller , S. Duan , A. Kreso , D. Barsyte‐Lovejoy , B. G. Wouters , J. Jin , D. D. Carvalho , M. Lupien , C. H. Arrowsmith , C. A. O'Brien , Nat. Commun. 2019, 10, 1436. 30926792 10.1038/s41467-019-09309-4 PMC6441108

[4] M. Wassef , A. Luscan , S. Aflaki , D. Zielinski , P. Jansen , H. I. Baymaz , A. Battistella , C. Kersouani , N. Servant , M. R. Wallace , P. Romero , O. Kosmider , P. A. Just , M. Hivelin , S. Jacques , A. Vincent‐Salomon , M. Vermeulen , M. Vidaud , E. Pasmant , R. Margueron , Proc. Natl. Acad. Sci. USA 2019, 116, 6075. 30867289 10.1073/pnas.1814634116 PMC6442582

[5] S. Chen , Q. Wang , H. Yu , M. L. Capitano , S. Vemula , S. C. Nabinger , R. Gao , C. Yao , M. Kobayashi , Z. Geng , A. Fahey , D. Henley , S. Z. Liu , S. Barajas , W. Cai , E. R. Wolf , B. Ramdas , Z. Cai , H. Gao , N. Luo , Y. Sun , T. N. Wong , D. C. Link , Y. Liu , H. S. Boswell , L. D. Mayo , G. Huang , R. Kapur , M. C. Yoder , H. E. Broxmeyer , et al., Nat. Commun. 2019, 10, 5649. 31827082 10.1038/s41467-019-13542-2 PMC6906427

[6] Z. A. Qadeer , D. Valle‐Garcia , D. Hasson , Z. Sun , A. Cook , C. Nguyen , A. Soriano , A. Ma , L. M. Griffiths , M. Zeineldin , D. Filipescu , L. Jubierre , A. Chowdhury , O. Deevy , X. Chen , D. B. Finkelstein , A. Bahrami , E. Stewart , S. Federico , S. Gallego , F. Dekio , M. Fowkes , D. Meni , J. M. Maris , W. A. Weiss , S. S. Roberts , N. V. Cheung , J. Jin , M. F. Segura , M. A. Dyer , et al., Cancer Cell 2019, 36, 512. 31631027 10.1016/j.ccell.2019.09.002 PMC6851493

[7] I. Singh , A. Contreras , J. Cordero , K. Rubio , S. Dobersch , S. Gunther , S. Jeratsch , A. Mehta , M. Kruger , J. Graumann , W. Seeger , G. Dobreva , T. Braun , G. Barreto , Nat. Genet. 2018, 50, 990. 29867223 10.1038/s41588-018-0139-3

[8] B. Rondinelli , E. Gogola , H. Yucel , A. A. Duarte , M. van de Ven , R. van der Sluijs , P. A. Konstantinopoulos , J. Jonkers , R. Ceccaldi , S. Rottenberg , A. D. D'Andrea , Nat. Cell Biol. 2017, 19, 1371. 29035360 10.1038/ncb3626

[9] P. Delgado‐Olguin , Y. Huang , X. Li , D. Christodoulou , C. E. Seidman , J. G. Seidman , A. Tarakhovsky , B. G. Bruneau , Nat. Genet. 2012, 44, 343. 22267199 10.1038/ng.1068 PMC3288669

[10] A. Karoutas , W. Szymanski , T. Rausch , S. Guhathakurta , E. A. Rog‐Zielinska , R. Peyronnet , J. Seyfferth , H. R. Chen , R. de Leeuw , B. Herquel , H. Kimura , G. Mittler , P. Kohl , O. Medalia , J. O. Korbel , A. Akhtar , Nat. Cell Biol. 2019, 21, 1248. 31576060 10.1038/s41556-019-0397-z

[11] D. Tumes , K. Hirahara , M. Papadopoulos , K. Shinoda , A. Onodera , J. Kumagai , K. H. Yip , H. Pant , K. Kokubo , M. Kiuchi , A. Aoki , K. Obata‐Ninomiya , K. Tokoyoda , Y. Endo , M. Y. Kimura , T. Nakayama , J. Allergy Clin. Immunol. 2019, 144, 549. 30851295 10.1016/j.jaci.2019.02.024

[12] J. C. Hellmuth , A. J. Louissaint , M. Szczepanowski , S. Haebe , A. Pastore , S. Alig , A. M. Staiger , S. Hartmann , R. Kridel , M. D. Ducar , P. Koch , M. Dreyling , M. L. Hansmann , G. Ott , A. Rosenwald , R. D. Gascoyne , D. M. Weinstock , W. Hiddemann , W. Klapper , O. Weigert , Blood 2018, 132, 1695. 30126979 10.1182/blood-2018-03-837252

[13] J. Zhou , S. Huang , Z. Wang , J. Huang , L. Xu , X. Tang , Y. Y. Wan , Q. J. Li , A. Symonds , H. Long , B. Zhu , Nat. Commun. 2019, 10, 2427. 31160593 10.1038/s41467-019-10176-2 PMC6547712

[14] C. Booth , N. Barkas , W. H. Neo , H. Boukarabila , E. J. Soilleux , G. Giotopoulos , N. Farnoud , A. Giustacchini , N. Ashley , J. Carrelha , L. Jamieson , D. Atkinson , T. Bouriez‐Jones , R. K. Prinjha , T. A. Milne , D. T. Teachey , E. Papaemmanuil , B. Huntly , S. Jacobsen , A. J. Mead , Cancer Cell 2018, 33, 274. 29438697 10.1016/j.ccell.2018.01.006

[15] E. Kim , M. Kim , D. Woo , Y. Shin , J. Shin , N. Chang , Y. T. Oh , H. Kim , J. Rheey , I. Nakano , C. Lee , K. M. Joo , J. N. Rich , D. Nam , J. Lee , Cancer Cell 2013, 23, 839. 23684459 10.1016/j.ccr.2013.04.008 PMC4109796

[16] K. H. Kim , C. W. Roberts , Nat. Med. 2016, 22, 128. 26845405 10.1038/nm.4036 PMC4918227

[17] P. Cohen , Annu. Rev. Biochem. 1989, 58, 453. 2549856 10.1146/annurev.bi.58.070189.002321

[18] T. Hunter , M. Karin , Cell 1992, 70, 375. 1643656 10.1016/0092-8674(92)90162-6

[19] T. Pawson , Nature 1995, 373, 573. 7531822 10.1038/373573a0

[20] T. L. Cha , B. P. Zhou , W. Xia , Y. Wu , C. C. Yang , C. T. Chen , B. Ping , A. P. Otte , M. C. Hung , Science 2005, 310, 306. 16224021 10.1126/science.1118947

[21] S. Chen , L. R. Bohrer , A. N. Rai , Y. Pan , L. Gan , X. Zhou , A. Bagchi , J. A. Simon , H. Huang , Nat. Cell Biol. 2010, 12, 1108. 20935635 10.1038/ncb2116 PMC3292434

[22] D. J. Mulholland , L. M. Tran , Y. Li , H. Cai , A. Morim , S. Wang , S. Plaisier , I. P. Garraway , J. Huang , T. G. Graeber , H. Wu , Cancer Cell 2011, 19, 792. 21620777 10.1016/j.ccr.2011.05.006 PMC3157296

[23] M. W. Schulz , C. G. Chamberlain , R. U. D. Iongh , J. W. McAvoy , Development 1993, 118, 117. 7690700 10.1242/dev.118.1.117

[24] D. G. Cogan , N. Engl. J. Med. 1973, 288, 1239. 4700558 10.1056/NEJM197306072882313

[25] D. Y. Shu , F. J. Lovicu , Prog. Retinal Eye Res. 2017, 60, 44. 10.1016/j.preteyeres.2017.08.001 PMC5600870 28807717

[26] E. D. Wederell , R. U. de Iongh , Semin. Cell Dev. Biol. 2006, 17, 759. 17134921 10.1016/j.semcdb.2006.10.006

[27] S. Saika , T. Miyamoto , S. Tanaka , T. Tanaka , I. Ishida , Y. Ohnishi , A. Ooshima , T. Ishiwata , G. Asano , T. Chikama , A. Shiraishi , C. Y. Liu , C. W. Kao , W. W. Kao , Invest. Ophthalmol. Visual Sci. 2003, 44, 2094. 12714648 10.1167/iovs.02-1059

[28] R. U. de Iongh , E. Wederell , F. J. Lovicu , J. W. McAvoy , Cells Tissues Organs 2005, 179, 43. 15942192 10.1159/000084508

[29] A. Banh , P. A. Deschamps , J. Gauldie , P. A. Overbeek , J. G. Sivak , J. A. West‐Mays , Invest. Ophthalmol. Visual Sci. 2006, 47, 3450. 16877415 10.1167/iovs.05-1208 PMC2811063

[30] J. V. Robertson , Z. Nathu , A. Najjar , D. Dwivedi , J. Gauldie , J. A. West‐Mays , Mol. Vision 2007, 13, 457. PMC2647562 17417606

[31] V. N. Simirskii , Y. Wang , M. K. Duncan , Dev. Biol. 2007, 306, 658. 17493607 10.1016/j.ydbio.2007.04.004 PMC1950782

[32] J. L. Walker , I. M. Wolff , L. Zhang , A. S. Menko , Invest. Ophthalmol. Visual Sci. 2007, 48, 2214. 17460282 10.1167/iovs.06-1059

[33] N. Gotoh , N. R. Perdue , H. Matsushima , E. H. Sage , Q. Yan , J. I. Clark , Invest. Ophthalmol. Visual Sci. 2007, 48, 4679. 17898292 10.1167/iovs.07-0091

[34] K. Yao , P. P. Ye , J. Tan , X. J. Tang , T. X. Shen , Ophthalmic Res. 2008, 40, 69. 18223299 10.1159/000113884

[35] C. C. Chong , R. J. Stump , F. J. Lovicu , J. W. McAvoy , Exp. Eye Res. 2009, 88, 307. 18789926 10.1016/j.exer.2008.07.018 PMC2683259

[36] Z. Nathu , D. J. Dwivedi , J. R. Reddan , H. Sheardown , P. J. Margetts , J. A. West‐Mays , Exp. Eye Res. 2009, 88, 323. 18809398 10.1016/j.exer.2008.08.014 PMC3408229

[37] X. Wang , B. Wang , N. Zhao , C. Wang , M. Huang , B. Chen , J. Chen , Y. Sun , L. Xiong , S. Huang , Y. Liu , Invest. Ophthalmol. Visual Sci. 2019, 60, 4748. 31731295 10.1167/iovs.19-27596

[38] E. H. Shin , M. A. Basson , M. L. Robinson , J. W. McAvoy , F. J. Lovicu , Mol. Med. 2012, 18, 861. 22517312 10.2119/molmed.2012.00111 PMC3409273

[39] I. F. Yamben , R. A. Rachel , S. Shatadal , N. G. Copeland , N. A. Jenkins , S. Warming , A. E. Griep , Dev. Biol. 2013, 384, 41. 24095903 10.1016/j.ydbio.2013.09.027 PMC3853123

[40] F. A. Mamuya , Y. Wang , V. H. Roop , D. A. Scheiblin , J. C. Zajac , M. K. Duncan , J. Cell. Mol. Med. 2014, 18, 656. 24495224 10.1111/jcmm.12213 PMC4000117

[41] D. Y. Shu , K. Ong , F. J. Lovicu , Optom. Vision Sci. 2017, 94, 270. 10.1097/OPX.0000000000001011 27801692

[42] L. Xie , P. Santhoshkumar , L. W. Reneker , K. K. Sharma , Invest. Ophthalmol. Vision Sci. 2014, 55, 4731. 10.1167/iovs.14-14109 24994865

[43] F. J. Lovicu , E. H. Shin , J. W. McAvoy , Exp. Eye Res. 2016, 142, 92. 26003864 10.1016/j.exer.2015.02.004 PMC4654713

[44] D. Y. Shu , M. C. Wojciechowski , F. J. Lovicu , Invest. Ophthalmol. Vision Sci. 2017, 58, 781. 10.1167/iovs.16-20611 PMC5295783 28152139

[45] X. Chen , W. Xiao , W. Chen , X. Liu , M. Wu , Q. Bo , Y. Luo , S. Ye , Y. Cao , Y. Liu , Cell Death Differ. 2017, 24, 1431. 28622289 10.1038/cdd.2016.152 PMC5520447

[46] Z. Wei , J. Caty , J. Whitson , A. D. Zhang , R. Srinivasagan , T. J. Kavanagh , H. Yan , X. Fan , Am. J. Pathol. 2017, 187, 2399. 28827139 10.1016/j.ajpath.2017.07.018 PMC5809338

[47] J. A. Whitson , P. A. Wilmarth , J. Klimek , V. M. Monnier , L. David , X. Fan , Free Radicals Biol. Med. 2017, 113, 84. 10.1016/j.freeradbiomed.2017.09.019 PMC5699945 28951044

[48] J. A. Whitson , X. Zhang , M. Medvedovic , J. Chen , Z. Wei , V. M. Monnier , X. Fan , Invest. Ophthalmol. Vision Sci. 2017, 58, 2666. 10.1167/iovs.16-21398 PMC5444549 28525556

[49] R. B. Nahomi , R. H. Nagaraj , J. Cell. Biochem. 2018, 119, 6814. 29693273 10.1002/jcb.26877 PMC6605039

[50] S. Das , P. Wikstrom , E. Walum , F. J. Lovicu , Exp. Eye Res. 2019, 185, 107692. 31189078 10.1016/j.exer.2019.107692

[51] D. Y. Shu , F. J. Lovicu , Exp. Eye Res. 2019, 185, 107693. 31201806 10.1016/j.exer.2019.107693

[52] M. H. Nam , A. Smith , M. B. Pantcheva , K. U. Park , J. A. Brzezinski , J. J. Galligan , K. Fritz , I. M. Wormstone , R. H. Nagaraj , Biochem. J. 2020, 477, 75. 31815277 10.1042/BCJ20190540 PMC8259308

[53] Y. Luo , A. R. Shoemaker , X. Liu , K. W. Woods , S. A. Thomas , R. D. Jong , E. K. Han , T. Li , V. S. Stoll , J. A. Powlas , A. Oleksijew , M. J. Mitten , Y. Shi , R. Guan , T. P. McGonigal , V. Klinghofer , E. F. Johnson , J. D. Leverson , J. J. Bouska , M. Mamo , R. A. Smith , E. E. Gramling‐Evans , B. A. Zinker , A. K. Mika , P. T. Nguyen , T. Oltersdorf , S. H. Rosenberg , Q. Li , V. L. Giranda , Mol. Cancer Ther. 2005, 4, 977. 15956255 10.1158/1535-7163.MCT-05-0005

[54] J. J. Caldwell , T. G. Davies , A. Donald , T. McHardy , M. G. Rowlands , G. W. Aherne , L. K. Hunter , K. Taylor , R. Ruddle , F. I. Raynaud , M. Verdonk , P. Workman , M. D. Garrett , I. Collins , J. Med. Chem. 2008, 51, 2147. 18345609 10.1021/jm701437d

[55] G. B. Moorhead , L. Trinkle‐Mulcahy , A. Ulke‐Lemee , Nat. Rev. Mol. Cell Biol. 2007, 8, 234. 17318227 10.1038/nrm2126

[56] D. J. Prockop , K. I. Kivirikko , Annu. Rev. Biochem. 1995, 64, 403. 7574488 10.1146/annurev.bi.64.070195.002155

[57] M. D. Shoulders , R. T. Raines , Annu. Rev. Biochem. 2009, 78, 929. 19344236 10.1146/annurev.biochem.77.032207.120833 PMC2846778

[58] R. V. Iozzo , Annu. Rev. Biochem. 1998, 67, 609. 9759499 10.1146/annurev.biochem.67.1.609

[59] Y. Wei , Y. Chen , L. Li , J. Lang , S. Yeh , B. Shi , C. Yang , J. Yang , C. Lin , C. Lai , M. Hung , Nat. Cell Biol. 2011, 13, 87. 21131960 10.1038/ncb2139 PMC3076036

[60] L. Nie , Y. Wei , F. Zhang , Y. Hsu , L. Chan , W. Xia , B. Ke , C. Zhu , R. Deng , J. Tang , J. Yao , Y. Chu , X. Zhao , Y. Han , J. Hou , L. Huo , H. Ko , W. Lin , H. Yamaguchi , J. Hsu , Y. Yang , D. N. Pan , J. L. Hsu , C. G. Kleer , N. E. Davidson , G. N. Hortobagyi , M. Hung , Nat. Commun. 2019, 10, 5114. 31704972 10.1038/s41467-019-13105-5 PMC6841924

[61] B. Li , J. Yan , T. Phyu , S. Fan , T. H. Chung , N. Mustafa , B. Lin , L. Wang , P. Eichhorn , B. C. Goh , S. B. Ng , D. Kappei , W. J. Chng , Blood 2019, 134, 2046. 31434700 10.1182/blood.2019000381

[62] A. R. Özeş , N. Pulliam , M. G. Ertosun , Ö. Yılmaz , J. Tang , E. Çopuroğlu , D. Matei , O. N. Özeş , K. P. Nephew , Oncogene 2018, 37, 3589. 29576612 10.1038/s41388-018-0218-z PMC6023775

[63] L. Wan , K. Xu , Y. Wei , J. Zhang , T. Han , C. Fry , Z. Zhang , Y. V. Wang , L. Huang , M. Yuan , W. Xia , W. Chang , W. Huang , C. Liu , Y. Chang , J. Liu , Y. Wu , V. X. Jin , X. Dai , J. Guo , J. Liu , S. Jiang , J. Li , J. M. Asara , M. Brown , M. Hung , W. Wei , Mol. Cell 2018, 69, 279. 29351847 10.1016/j.molcel.2017.12.024 PMC5777296

[64] T. Anwar , C. Arellano‐Garcia , J. Ropa , Y. Chen , H. S. Kim , E. Yoon , S. Grigsby , V. Basrur , A. I. Nesvizhskii , A. Muntean , M. E. Gonzalez , K. M. Kidwell , Z. Nikolovska‐Coleska , C. G. Kleer , Nat. Commun. 2018, 9, 2801. 30022044 10.1038/s41467-018-05078-8 PMC6051995

[65] J. Yan , B. Li , B. Lin , P. T. Lee , T. H. Chung , J. Tan , C. Bi , X. T. Lee , V. Selvarajan , S. B. Ng , H. Yang , Q. Yu , W. J. Chng , Blood 2016, 128, 948. 27297789 10.1182/blood-2016-01-690701

[66] H. Sun , X. Yang , L. Liang , M. Zhang , Y. Li , J. Chen , F. Wang , T. Yang , F. Meng , X. Lai , C. Li , J. He , M. He , Q. Xu , Q. Li , L. Lin , D. Pei , H. Zheng , EMBO J. 2020, 39, 102961. 10.15252/embj.2019102961 PMC7156961 32090361

[67] J. Ma , J. Zhang , Y. C. Weng , J. C. Wang , Mol. Cells 2018, 41, 868. 30304920 10.14348/molcells.2018.0109 PMC6182224

[68] J. Yang , B. Tian , H. Sun , R. P. Garofalo , A. R. Brasier , Nat. Microbiol. 2017, 2, 17086. 28581456 10.1038/nmicrobiol.2017.86 PMC5501188

[69] D. Y. Shu , M. Wojciechowski , F. J. Lovicu , Exp. Eye Res. 2019, 178, 108. 30290164 10.1016/j.exer.2018.09.021

[70] L. Xiao , L. L. Gong , D. Yuan , M. Deng , X. M. Zeng , L. L. Chen , L. Zhang , Q. Yan , J. P. Liu , X. H. Hu , S. M. Sun , J. Liu , H. L. Ma , C. B. Zheng , H. Fu , P. C. Chen , J. Q. Zhao , S. S. Xie , L. J. Zou , Y. M. Xiao , W. B. Liu , J. Zhang , Y. Liu , D. W. Li , Cell Death Differ. 2010, 17, 1448. 20186153 10.1038/cdd.2010.16

[71] L. Zhang , S. Sun , J. Zhou , J. Liu , J. H. Lv , X. Q. Yu , C. Li , L. Gong , Q. Yan , M. Deng , L. Xiao , H. Ma , J. P. Liu , Y. L. Peng , D. Wang , G. P. Liao , L. J. Zou , W. B. Liu , Y. M. Xiao , D. W. Li , Antioxid. Redox Signaling 2011, 15, 1. 10.1089/ars.2010.3560 PMC3110099 21303257

[72] Q. Yang , W. Jiang , P. Hou , Semin. Cancer Biol. 2019, 59, 112. 30951826 10.1016/j.semcancer.2019.04.001

[73] K. Xu , Z. J. Wu , A. C. Groner , H. H. He , C. Cai , R. T. Lis , X. Wu , E. C. Stack , M. Loda , T. Liu , H. Xu , L. Cato , J. E. Thornton , R. I. Gregory , C. Morrissey , R. L. Vessella , R. Montironi , C. Magi‐Galluzzi , P. W. Kantoff , S. P. Balk , X. S. Liu , M. Brown , Science 2012, 338, 1465. 23239736 10.1126/science.1227604 PMC3625962

[74] F. Li , Z. Zeng , S. Xing , J. A. Gullicksrud , Q. Shan , J. Choi , V. P. Badovinac , S. Crotty , W. Peng , H. H. Xue , Nat. Commun. 2018, 9, 5452. 30575739 10.1038/s41467-018-07853-z PMC6303346

[75] M. Ferreira , I. Verbinnen , M. Fardilha , A. Van Eynde , M. Bollen , J. Biol. Chem. 2018, 293, 18031. 30305391 10.1074/jbc.AC118.005577 PMC6254331

[76] X. Lin , X. Duan , Y. Y. Liang , Y. Su , K. H. Wrighton , J. Long , M. Hu , C. M. Davis , J. Wang , F. C. Brunicardi , Y. Shi , Y. G. Chen , A. Meng , X. H. Feng , Cell 2016, 166, 1597. 27610577 10.1016/j.cell.2016.08.062

[77] C. J. Chang , C. H. Chao , W. Xia , J. Y. Yang , Y. Xiong , C. W. Li , W. H. Yu , S. K. Rehman , J. L. Hsu , H. H. Lee , M. Liu , C. T. Chen , D. Yu , M. C. Hung , Nat. Cell Biol. 2011, 13, 317. 21336307 10.1038/ncb2173 PMC3075845

[78] D. W. Li , J. P. Liu , P. C. Schmid , R. Schlosser , H. Feng , W. B. Liu , Q. Yan , L. Gong , S. M. Sun , M. Deng , Y. Liu , Oncogene 2006, 25, 3006. 16501611 10.1038/sj.onc.1209334

[79] R. F. Diegelmann , J. Urol. 1997, 157, 298. 8976284

[80] A. Leask , D. J. Abraham , FASEB J. 2004, 18, 816. 15117886 10.1096/fj.03-1273rev

[81] G. Gabbiani , J. Pathol. 2003, 200, 500. 12845617 10.1002/path.1427

[82] K. Zhang , M. D. Rekhter , D. Gordon , S. H. Phan , Am. J. Pathol. 1994, 145, 114. 7518191 PMC1887314

[83] D. C. Radisky , P. A. Kenny , M. J. Bissell , J. Cell. Biochem. 2007, 101, 830. 17211838 10.1002/jcb.21186 PMC2838476

[84] S. Saika , O. Yamanaka , Y. Okada , S. Tanaka , T. Miyamoto , T. Sumioka , A. Kitano , K. Shirai , K. Ikeda , Front. Biosci. 2009, 1, 376. 10.2741/S32 19482708

[85] O. Yamanaka , C. Y. Liu , W. W. Kao , Endocr., Metab. Immune Disord.: Drug Targets 2010, 10, 331. 20925651 10.2174/1871530311006040331

[86] J. A. Eldred , L. J. Dawes , I. M. Wormstone , Philos. Trans. R. Soc., B 2011, 366, 1301. 10.1098/rstb.2010.0341 PMC3061111 21402588

[87] J. Tang , I. J. Salzman , M. D. Sable , J. Cataract Refractive Surg. 2003, 29, 1641. 10.1016/s0886-3350(03)00120-2 12954322

[88] K. Sasaki , M. Kojima , H. Nakaizumi , K. Kitagawa , Y. Yamada , H. Ishizaki , Ophthalmologica 1998, 212, 88. 9486546 10.1159/000027285

[89] J. M. Marcantonio , P. P. Syam , C. S. Liu , G. Duncan , Exp. Eye Res. 2003, 77, 339. 12907166 10.1016/s0014-4835(03)00125-8

[90] T. Hatae , T. Ishibashi , F. Yoshitomi , Y. Shibata , Graefe's Arch. Clin. Exp. Ophthalmol. 1993, 231, 586. 8224934 10.1007/BF00936523

[91] L. Morra , H. Moch , Virchows Arch. 2011, 459, 465. 21997759 10.1007/s00428-011-1151-5 PMC3205268

[92] H. Huang , H. Ruan , M. Y. Aw , A. Hussain , L. Guo , C. Gao , F. Qian , T. Leung , H. Song , D. Kimelman , Z. Wen , J. Peng , Development 2008, 135, 3209. 18776143 10.1242/dev.024406 PMC5574253

[93] Y. Zhong , Q. Ye , C. Chen , M. Wang , H. Wang , Nucleic Acids Res. 2018, 46, 3382. 29447387 10.1093/nar/gky101 PMC5909462

