# Supplementary material for: MYPT1/PP1‐Mediated EZH2 Dephosphorylation at S21 Promotes Epithelial–Mesenchymal Transition in Fibrosis through Control of Multiple Families of Genes
Source: Adv Sci (Weinh). 2022 Mar 16;9(14):2105539. doi: 10.1002/advs.202105539 (PMC9108659; doi:10.1002/advs.202105539)
Supplement: Supplementary file 1 — Supporting Information [file ADVS-9-2105539-s001.pdf]

ADVANCED SCIENCE
Open Access

# Supporting Information

for *Adv. Sci.*, DOI 10.1002/advs.202105539

MYPT1/PP1-Mediated EZH2 Dephosphorylation at S21 Promotes Epithelial–Mesenchymal Transition in Fibrosis through Control of Multiple Families of Genes

*Lan Zhang, Ling Wang, Xue-Bin Hu, Min Hou, Yuan Xiao, Jia-Wen Xiang, Jie Xie, Zhi-Gang Chen, Tian-Heng Yang, Qian Nie, Jia-Ling Fu, Yan Wang, Shu-Yu Zheng, Yun-Fei Liu, Yu-Wen Gan, Qian Gao, Yue-Yue Bai, Jing-Miao Wang, Rui-Li Qi, Ming Zou, Qin Ke, Xing-Fei Zhu, Lili Gong, Yizhi Liu* and David Wan-Cheng Li**

# SUPPLEMENTARY MATERIALS

## 1. Supplementary Figures and Legends

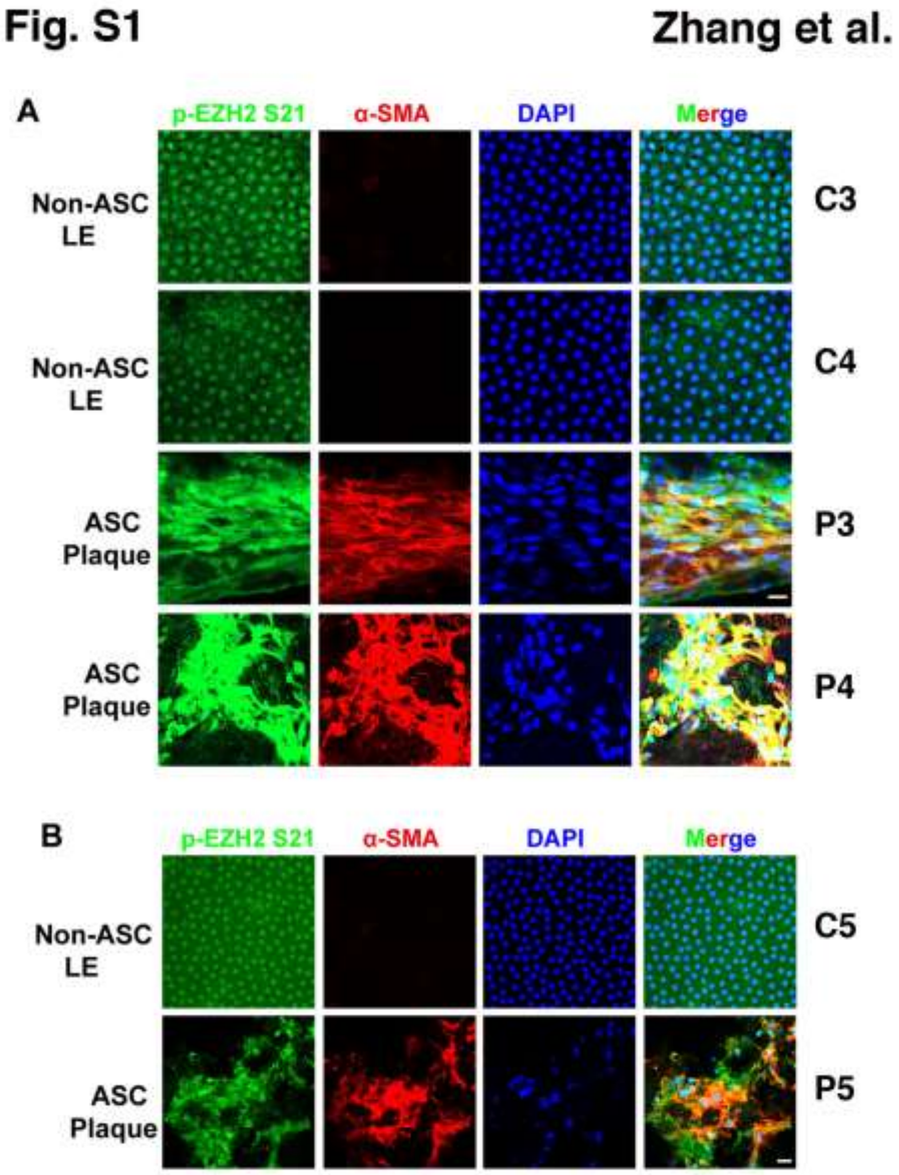


**Fig. S1. A, B.** Immunofluorescence staining for p-EZH2 S21 (green), α-SMA (red) and lens epithelial cell nuclei (blue) of lens capsular epithelium from non-ASC human lenses and ASC patients. C, non-ASC Control patient, P, ASC Patient.

**Fig. S2** **Zhang et al.**

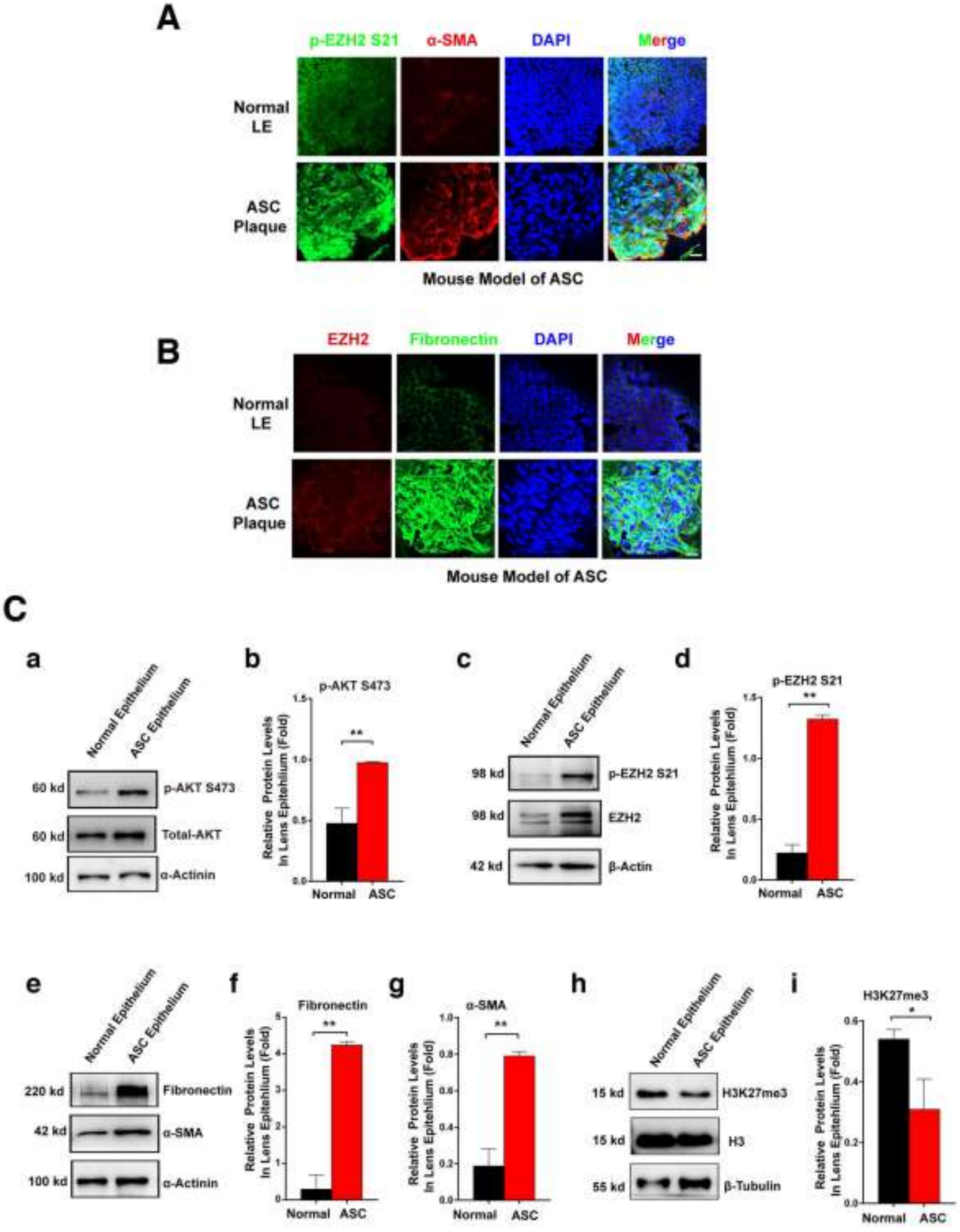

**Fig. S2. Expression of AKT-EZH2-H3K27me3 signal axis components in injury-induced mouse ASC. A.** The anterior capsules of one lens of the mouse was punctured with a needle to induce ASC, and the other lens remains as untreated normal. After 7 days of healing, immunofluorescence (**A & B)** and Western blot analyses(**C**) were used to analyze the protein expression levels of lens capsular epithelium from normal and injured-induced ASC mice. **A.** Immunofluorescence staining for p-EZH2 S21 (green), α-SMA (red) and lens epithelial cell nuclei (blue) (n = 3 lenses per group). **B.** Immunofluorescence staining of EZH2 (red), fibronectin (green) and lens epithelial cell nuclei (blue) (n = 3 lenses per group). **C-a**. Western blot analysis of the protein levels of p-AKTS473. **C-b.** Quantification of the protein expression levels of p-AKTS473 in (**C-a**)**. C-c.** Western blot analysis of the protein levels of p-EZH2 S21. **C-d.** Quantification of the protein expression levels of p-EZH2 S21 in (**C-c**)**. C-e.** Western blot analysis of the protein levels of Fibronectin and α-SMA. **C-f & C-g.** Quantification of the protein expression levels of Fibronectin and α-SMA in (**C-e**)**. C-h.** Western blot analysis of the protein levels of H3K27me3. **C-i**. Quantification of the protein expression levels of H3K27me3 in (**C-h**). Error bars represent the standard deviation of the mean (n=3). Statistical analysis: Two-tailed Student's t test; ** $p < 0.01$, * $p < 0.05$.

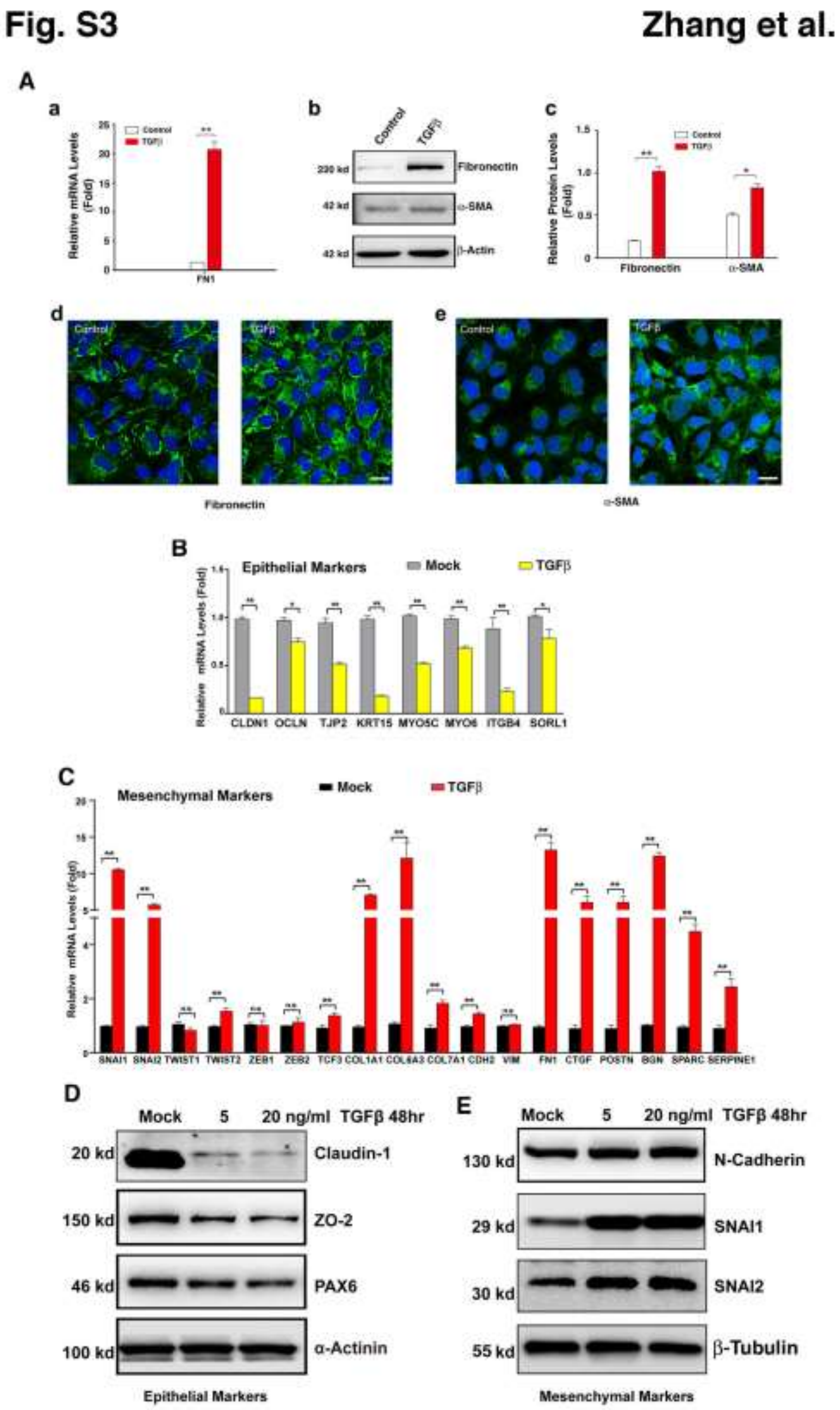
Fig. S3
Zhang et al.
A
a
Relative mRNA Levels (Fold)
Control
TGFβ
FN1
b
Control
TGFβ
230 kd
Fibronectin
42 kd
α-SMA
42 kd
β-Actin
c
Relative Protein Levels (Fold)
Control
TGFβ
Fibronectin
α-SMA
d
Control
TGFβ
Fibronectin
e
Control
TGFβ
α-SMA
B
Epithelial Markers
Mock
TGFβ
Relative mRNA Levels (Fold)
CLDN1 OCLN TJP2 KRT15 MYO5C MYO6 ITGB4 SORL1
C
Mesenchymal Markers
Mock
TGFβ
Relative mRNA Levels (Fold)
SNAI1 SNAI2 TWIST1 TWIST2 ZEB1 ZEB2 TCF3 COL1A1 COL6A3 COL7A1 CDH2 VIM FN1 CTGF POSTN BGN SPARC SERPINE1
D
Mock 5 20 ng/ml TGFβ 48hr
20 kd
Claudin-1
150 kd
ZO-2
46 kd
PAX6
100 kd
α-Actinin
Epithelial Markers
E
Mock 5 20 ng/ml TGFβ 48hr
130 kd
N-Cadherin
29 kd
SNAI1
30 kd
SNAI2
55 kd
β-Tubulin
Mesenchymal Markers

**Fig. S3. TGFβ induced EMT in HLE cells.** HLE cells were either mock-treated or treated with 20 ng/ml TGFβ for 48 h. **A-a.** qRT-PCR was used to examine the mRNA level of the FN1 gene. **A-b.** Western blot analysis of the protein levels of Fibronectin and α-SMA **A-c.** Quantification of the protein expression levels of Fibronectin and α-SMA in (**A-b**). **A-d & A-e.** Immunofluorescence staining for fibronectin and α-SMA proteins. Scale bar, 20 μm. **B.** qRT-PCR was used to verify the mRNA downregulation of the epithelial marker genes in TGFβ-treated cells. **C.** qRT-PCR was used to verify the mRNA upregulation of the mesenchymal marker genes in TGFβ-treated cells. **D.** Western blot analysis was used to verify protein downregulation of the epithelial marker genes during TGFβ induced-EMT in HLE cells. **E.** Western blot analysis was used to verify the protein upregulation of the mesenchymal marker genes during TGFβ induced-EMT in HLE cells. Error bars represent the standard deviation of the mean (n=3). Statistical analysis: Two-tailed Student's t test; ** $p < 0.01$, * $p < 0.05$.

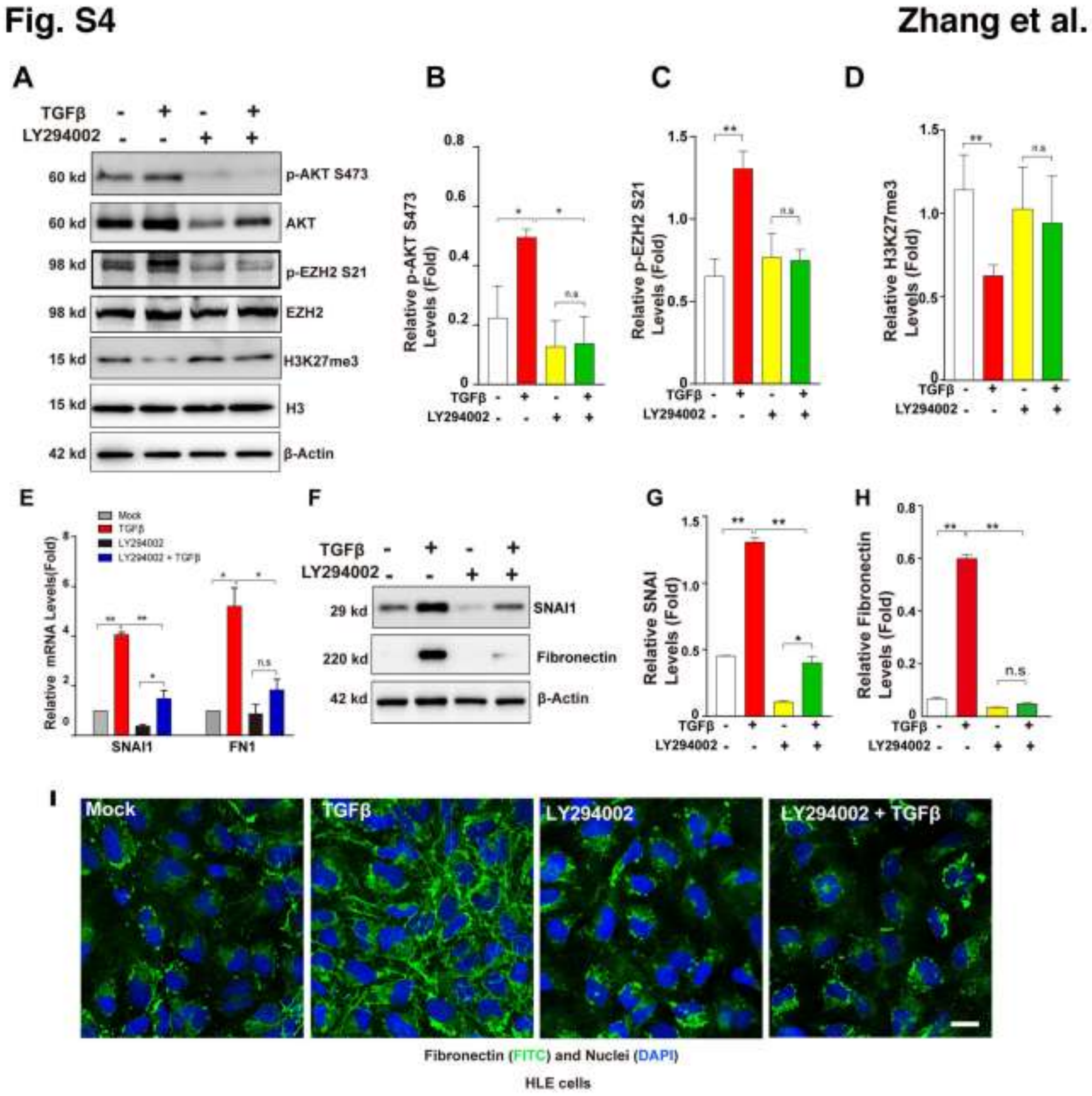
Fig. S4
Zhang et al.
A
TGFβ
LY294002
60 kd
p-AKT S473
AKT
98 kd
p-EZH2 S21
EZH2
15 kd
H3K27me3
H3
42 kd
β-Actin
B
Relative p-AKT S473 Levels (Fold)
C
Relative p-EZH2 S21 Levels (Fold)
D
Relative H3K27me3 Levels (Fold)
E
Mock
TGFβ
LY294002
LY294002 + TGFβ
Relative mRNA Levels(Fold)
SNAI1
FN1
F
29 kd
SNAI1
220 kd
Fibronectin
G
Relative SNAI Levels (Fold)
H
Relative Fibronectin Levels (Fold)
n.s
I
Mock
TGFβ
LY294002
LY294002 + TGFβ
Fibronectin (FITC) and Nuclei (DAPI)
HLE cells

**Fig. S4. Attenuated p-EZH2 S21 phosphorylation under PI3K inhibition led to suppressed TGFβ-induced EMT gene activation in HLE cells.** Mock and TGFβ-treated HLE cells were co-treated with 20 μM LY294002 or DMSO for 48 h. **A**. Western blot analysis of the protein levels of p-AKT S473, p-EZH2 S21 and H3K27me3. **B, C & D,** Quantification of the protein expression levels of p-AKT S473, p-EZH2 S21 and H3K27me3 in (A). **E.** qRT-PCR analysis was used to examine the mRNA levels of SNAI1 and FN1. **F.** Western blot analysis of the protein expression levels of SNAI1 and Fibronectin. **G & H.** Quantification results of SNAI1 and Fibronectin protein levels in (**F**). **I.** Immunofluorescence staining for Fibronectin proteins in the HLE cells with indicated treatment. Scale bar, 20 μm. In (B), (C), (D), (E), (G) and (H), error bars represent the standard deviation of the mean (n=3). Statistical analysis: Two-way ANOVA followed by Tukey's correction. ** $p < 0.01$, * $p < 0.05$. n.s, not significant.

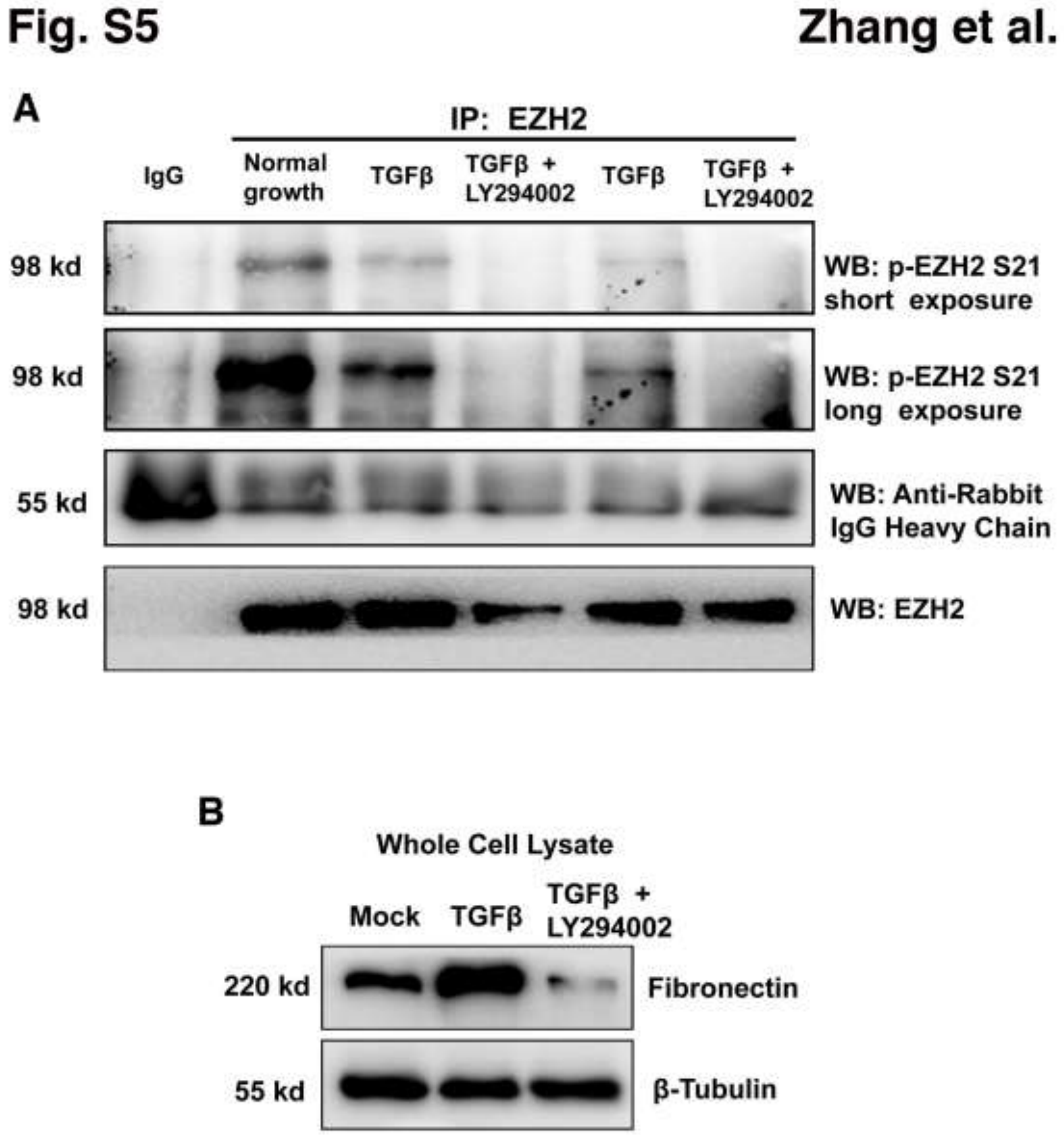


**Fig. S5**. TGFβ-induced EZH2 S21 phosphorylation is depleted under PI3K inhibition. **A.** Normal growth condition( containing 10% FBS) cultured HLE cells, 48 h of 20 ng $mL^{-1}$ TGFβ-treated HLE cells, 48 h of 20 ng $mL^{-1}$ TGFβ and 20 μM LY294002 co-treated HLE cells were subjected to EZH2 immunoprecipitation(IP), p-EZH2 S21 antibody was employed in the IP-linked western blot analysis to detect the S21 phosphorylation levels of EZH2 proteins. **B**. Western blot confirmation of LY294002 diminished TGFβ-induced Fibronectin protein expression.

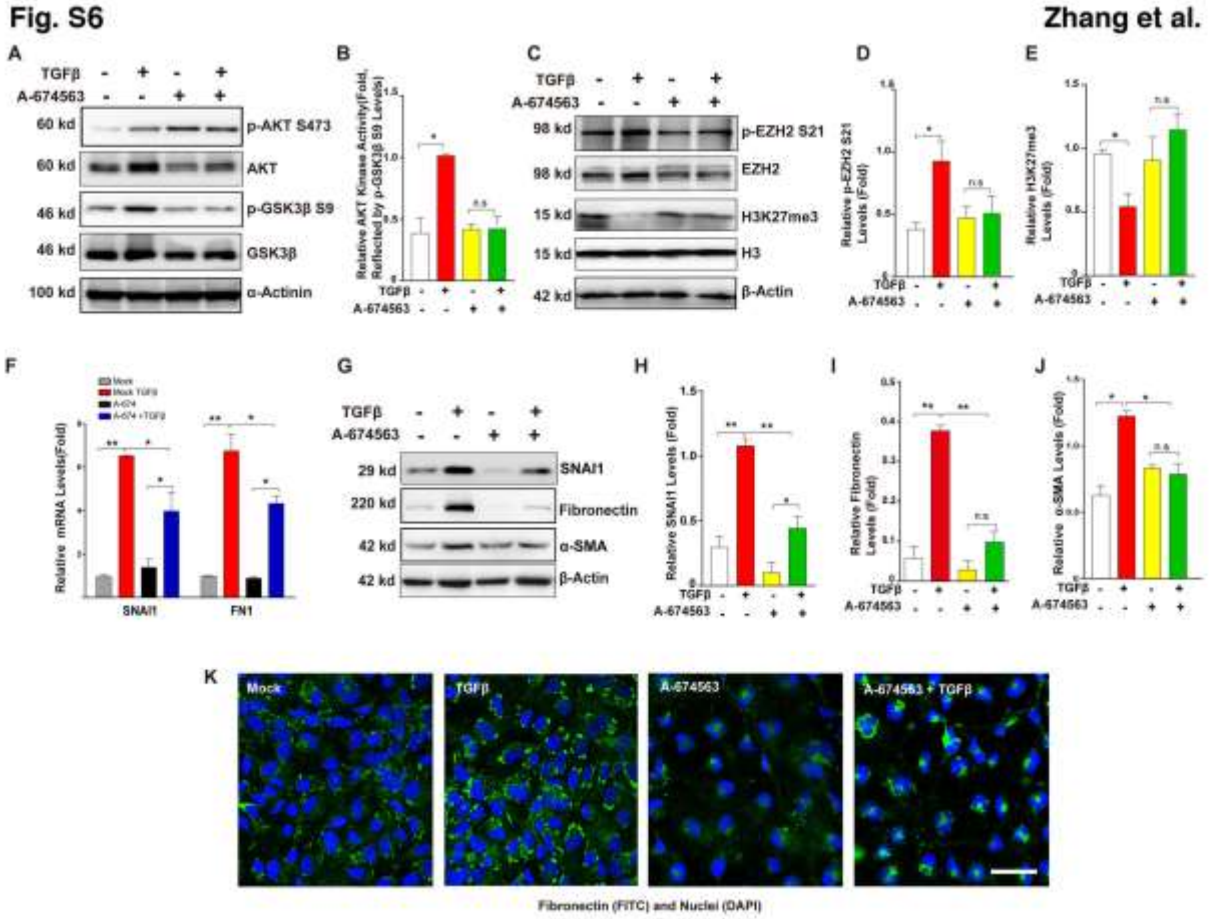


**Fig. S6. Attenuated p-EZH2 S21 phosphorylation under AKT1 inhibition led to suppressed TGFβ-induced EMT gene activation in HLE cells.** Mock and TGFβ treated-HLE cells were co-treated with 0.5μM AKT1 inhibitor A-674563 or DMSO for 48 h. **A**. Western blot analysis of the protein levels of p-AKT S473 and p-GSK3β S9. Note that in the presence of AKT1 inhibitor A-674563, there was concomitant increase of p-AKT S473, which has been previously documented with many AKT inhibitors, and seems a sensitive marker of AKT inhibition [53]. In spite of that, the AKT kinase activity reflected by the S9 phosphorylation of AKT substrate GSK3β was markedly impaired. **B.** Quantification AKT kinase activity reflected by the p-GSK3β S9 levels in (A). **C.** Western blot analysis of the protein levels of p-EZH2 S21 and H3K27me3. **D & E,** Quantification of the protein expression levels of p-EZH2 S21 and H3K27me3 in (C). **F.** qRT-PCR analysis was used to examine the mRNA levels of SNAI1 and FN1. **G.** Western blot analysis of the protein expression levels of SNAI1, Fibronectin and α-SMA. **H, I & J.** Quantification results of SNAI1, Fibronectin and α-SMA protein levels in (**G**). **I.** Immunofluorescence staining for the Fibronectin proteins in the HLE cells with indicated treatment. Scale bar, 20 μm. In (B), (D), (E),(F),(H),(I) and (J), error bars represent the standard deviation of the mean (n=3). Statistical analysis: Two-way ANOVA followed by Tukey's correction. ** $p < 0.01$, * $p < 0.05$. n.s, not significant.

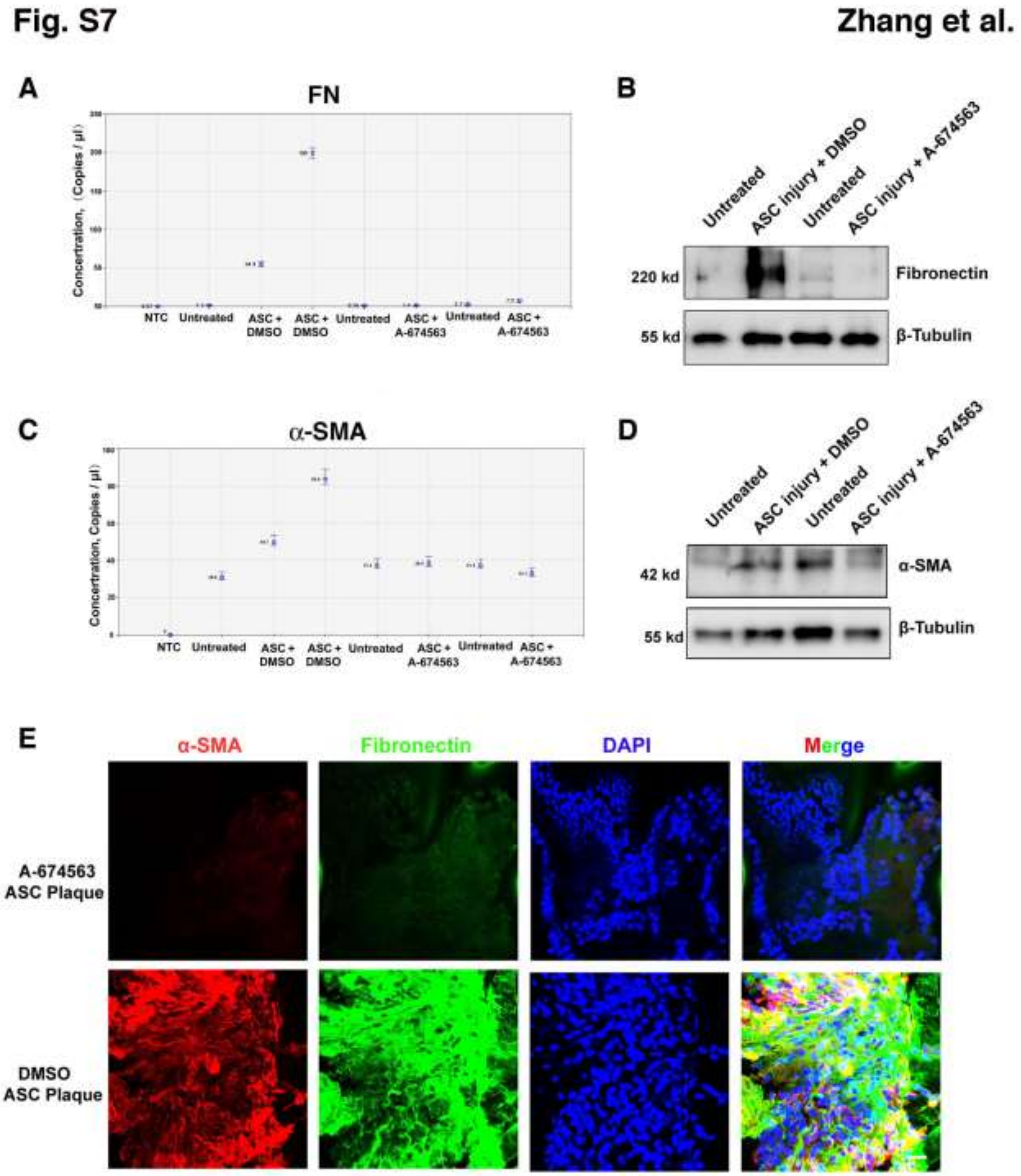
Fig. S7
Zhang et al.
A
FN
Concentration, (Copies / µl)
NTC
Untreated
ASC + DMSO
ASC + DMSO
Untreated
ASC + A-674563
Untreated
ASC + A-674563
B
Untreated
ASC injury + DMSO
Untreated
ASC injury + A-674563
220 kd
Fibronectin
55 kd
β-Tubulin
C
α-SMA
Concentration, Copies / µl)
NTC
Untreated
ASC + DMSO
ASC + DMSO
Untreated
ASC + A-674563
Untreated
ASC + A-674563
D
Untreated
ASC injury + DMSO
Untreated
ASC injury + A-674563
42 kd
α-SMA
55 kd
β-Tubulin
E
α-SMA
Fibronectin
DAPI
Merge
A-674563 ASC Plaque
DMSO ASC Plaque

**Fig. S7. AKT1 inhibitor attenuates mesenchymal gene expression in injury-induced ASC mouse model.** The anterior capsules of mouse lens were injured with a needle, and 1 μL of 20 μM of AKT1 inhibitor A-674563 or equal volume of DMSO were injected into the anterior chamber of the eye immediately after injury with a microsyringe. After 7 days of healing, lens capsules were harvested to analyze the gene expression from untreated lens epithelium, ASC lens epithelium with DMSO injection (ASC + DMSO), or ASC lens epithelium with A-674563 injection (ASC + A-674563). **A.** digital droplet PCR (ddPCR) determined the copy number of Fibronectin coding gene FN1. Error bars represent the standard error of the mean. **B.** Western blot analysis was used to show the protein expression of Fibronectin. **C.** ddPCR determined the copy number of α-SMA coding gene ACTA2. Error bars represent the standard error of the mean. **D.** Western blot analysis was used to show the protein expression of α-SMA. **E**. Immunofluorescence staining of α-SMA (red), Fibronectin (green) and lens epithelial cell nuclei (blue) of the ASC plaques from mouse eyes injected with A-674563 or DMSO control (n = 3 lenses per group)**.** Scale bar, 20 μm.

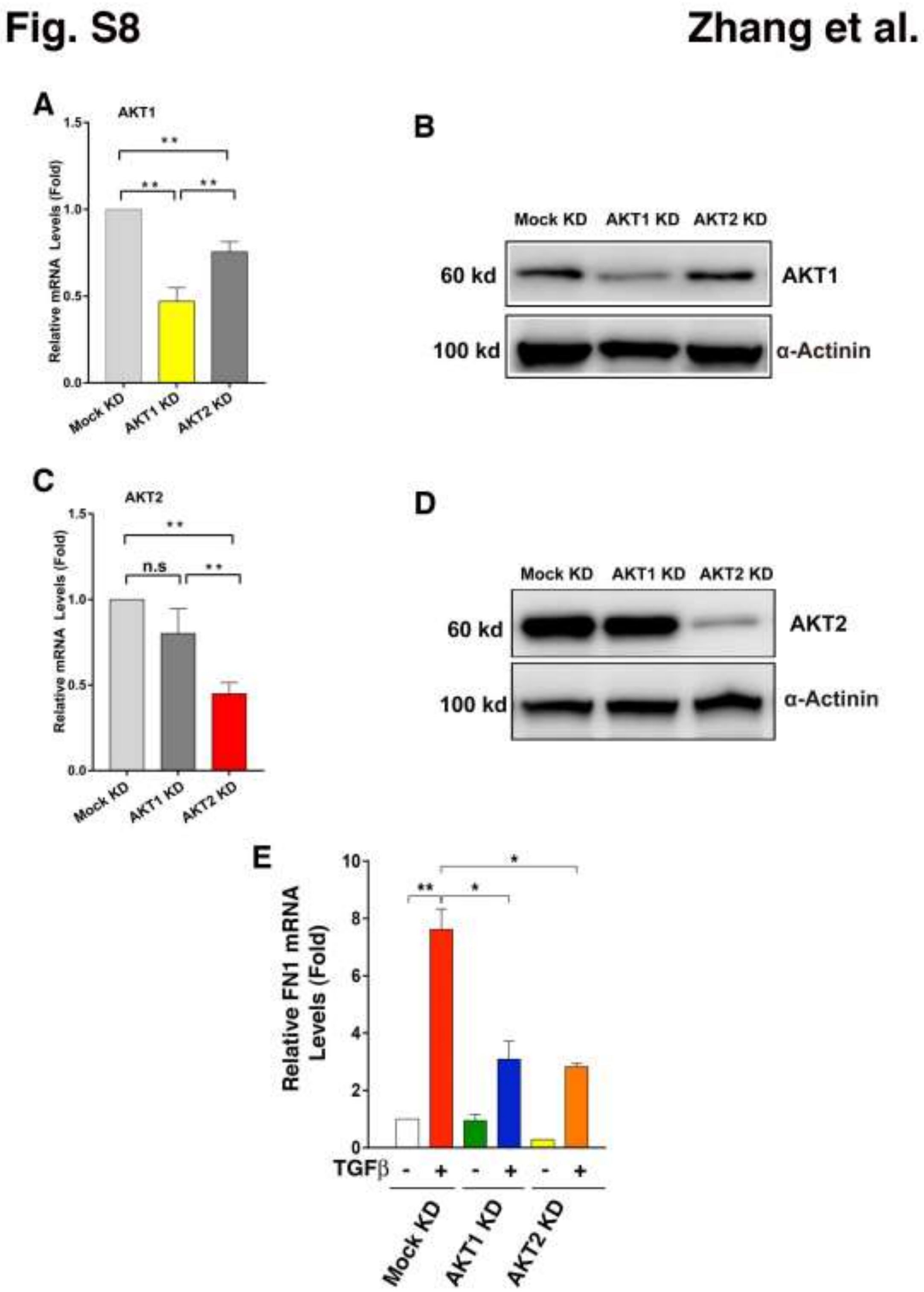

Fig. S8
Zhang et al.
A
AKT1
Relative mRNA Levels (Fold)
Mock KD
AKT1 KD
AKT2 KD
B
Mock KD AKT1 KD AKT2 KD
60 kd
AKT1
100 kd
α-Actinin
C
AKT2
n.s
Relative mRNA Levels (Fold)
Mock KD
AKT1 KD
AKT2 KD
D
Mock KD AKT1 KD AKT2 KD
60 kd
AKT2
100 kd
α-Actinin
E
Relative FN1 mRNA Levels (Fold)
TGFβ
Mock KD
AKT1 KD
AKT2 KD

**Fig. S8. Verification of AKT1 and AKT2 knockdown in HLE cells.** HLE Mock KD cells, HLE-AKT1 KD and HLE-AKT2 KD cells were subjected to mRNA and protein expression analyses. **A.** qRT-PCR was used to examine the mRNA expression levels of AKT1 isoform. **B**. Western blot analysis was used to examine the protein expression levels of AKT1 isoform. **C.** qRT-PCR was used to examine the mRNA expression levels of AKT2 isoform. **D**. Western blot analysis of the protein expression levels of AKT2. Note that, in spite of decreased AKT1 mRNA level in AKT2-KD cells, AKT1 protein expression was specifically silenced in AKT1-KD cells. In (A) and (C), error bars represent the standard deviation of the mean (n=3). Statistical analysis: One-way ANOVA; ** $p < 0.01$, * $p < 0.05$. **E.** qRT-PCR was used to examine the mRNA expression levels of FN1 in mock or TGFβ-treated-HLE Mock KD, AKT1 KD and AKT2 KD-HLE cells. Error bars represent the standard deviation of the mean (n=3). Statistical analysis: Two-way ANOVA followed by Tukey's correction. ** $p < 0.01$, * $p < 0.05$.

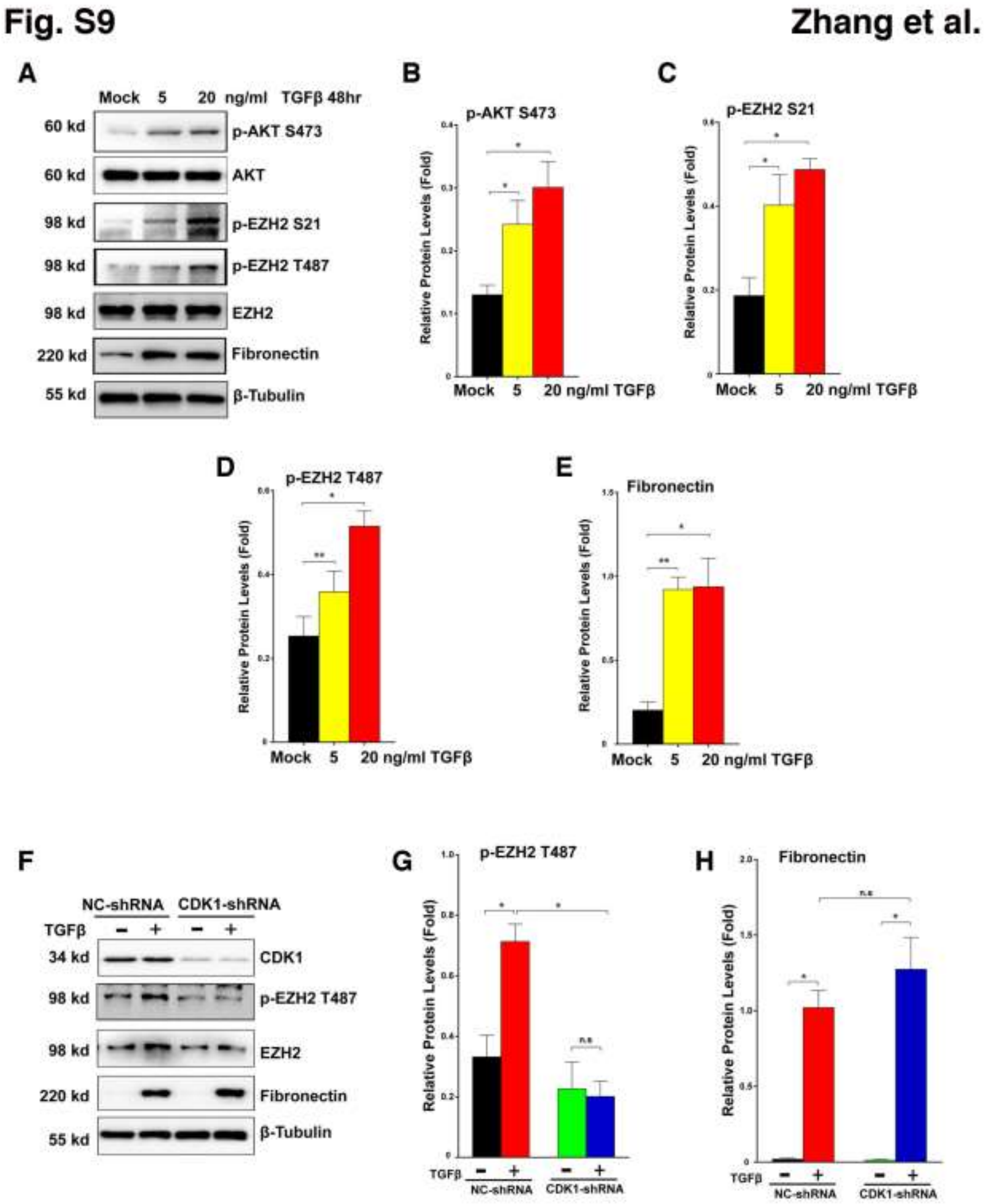
Fig. S9
Zhang et al.
A
Mock 5 20 ng/ml TGFβ 48hr
60 kd p-AKT S473
60 kd AKT
98 kd p-EZH2 S21
98 kd p-EZH2 T487
98 kd EZH2
220 kd Fibronectin
55 kd β-Tubulin
B
p-AKT S473
Relative Protein Levels (Fold)
Mock 5 20 ng/ml TGFβ
C
p-EZH2 S21
Relative Protein Levels (Fold)
Mock 5 20 ng/ml TGFβ
D
p-EZH2 T487
Relative Protein Levels (Fold)
Mock 5 20 ng/ml TGFβ
E
Fibronectin
Relative Protein Levels (Fold)
Mock 5 20 ng/ml TGFβ
F
NC-shRNA CDK1-shRNA
TGFβ − + − +
34 kd CDK1
98 kd p-EZH2 T487
98 kd EZH2
220 kd Fibronectin
55 kd β-Tubulin
G
p-EZH2 T487
Relative Protein Levels (Fold)
TGFβ − + − +
NC-shRNA CDK1-shRNA
H
Fibronectin
Relative Protein Levels (Fold)
TGFβ − + − +
NC-shRNA CDK1-shRNA

**Fig. S9. EZH2 T487 phosphorylation plays much less important role in controlling EMT of lens epithelial cells.** **A.** Western blot assay of the protein expression levels of p-AKT S473, p-EZH2 S21, p-EZH2 T487 and Fibronection under TGFβ treatment. **B, C, D & E.** Quantification of the protein expression levels of p-AKT S473, p-EZH2 S21, p-EZH2 T487 and Fibronectin in (A). Error bars represent the standard deviation of the mean(n=3). Statistical analysis: one-way ANOVA, ** $p < 0.01$, * $p < 0.05$. **F.** qRT-PCR was used to examine the mRNA levels of FN1, COL1A1, SNAI1 and SNAI2 in mock or TGFβ-treated HLE cells overexpressing negative control (NC) shRNA, CDK1-shRNA. **G**. Western blot analysis of the protein levels of CDK1, p-EZH2 T487 and Fibronectin in the mock or TGFβ-treated HLE cells overexpressing control NC shRNA or CDK1-shRNA. **H & I.** Quantification of the protein expression levels of p-EZH2 T487 and Fibronectin in (**G**).

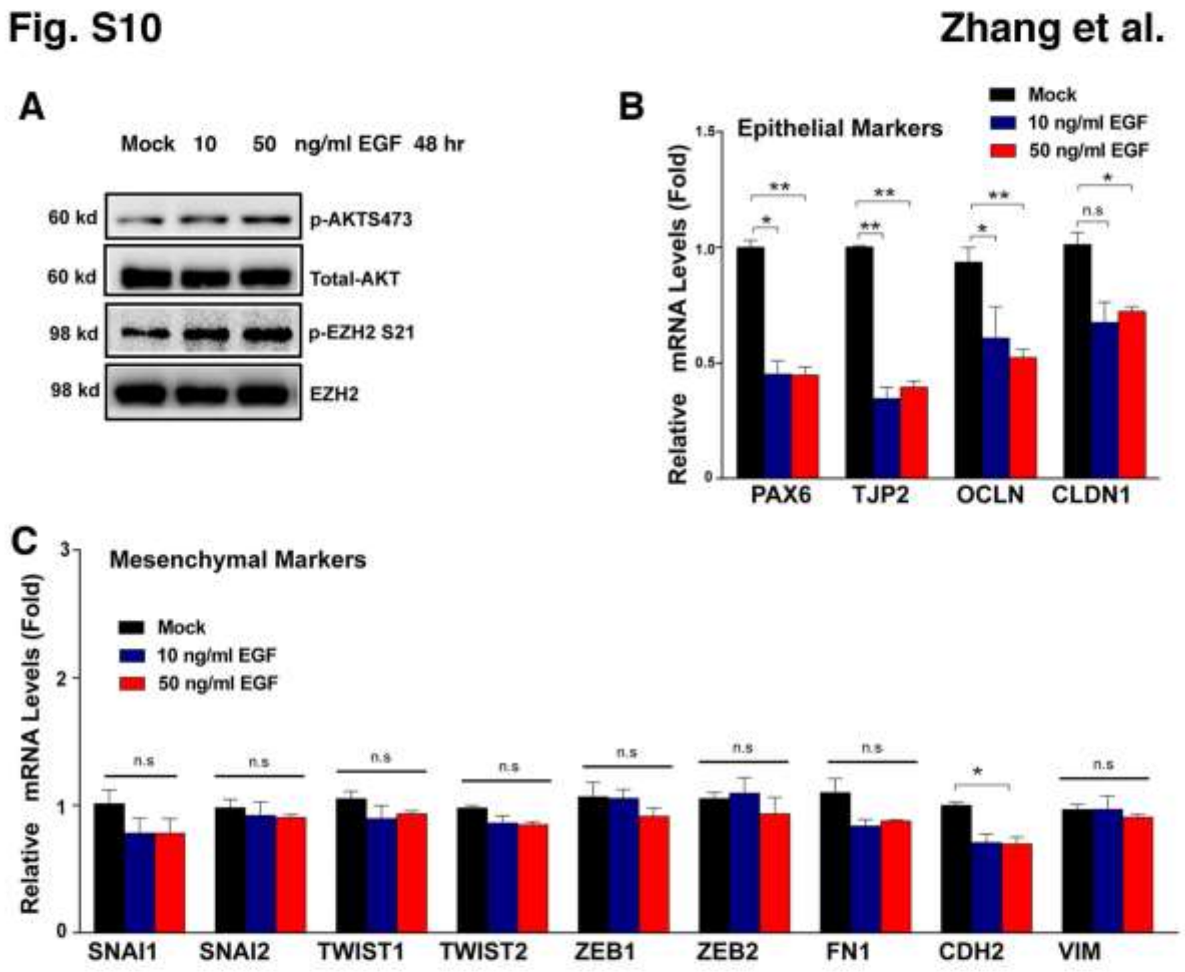


**Fig. S10. EGF has little effect on EMT gene expression in lens epithelial cells.** **A.** Western blot assay of the protein expression levels of p-AKT S473, p-EZH2 S21 under different concentrations of EGF treatment for 48 h. **B.** qRT-PCR was used to examine the mRNA downregulation of the epithelial marker genes. **C.** qRT-PCR was used to examine the mRNA expression levels of the mesenchymal marker genes. Error bars represent the standard deviation of the mean(n=3). Statistical analysis: one-way ANOVA, ** $p < 0.01$, * $p < 0.05$. n.s, not significant.

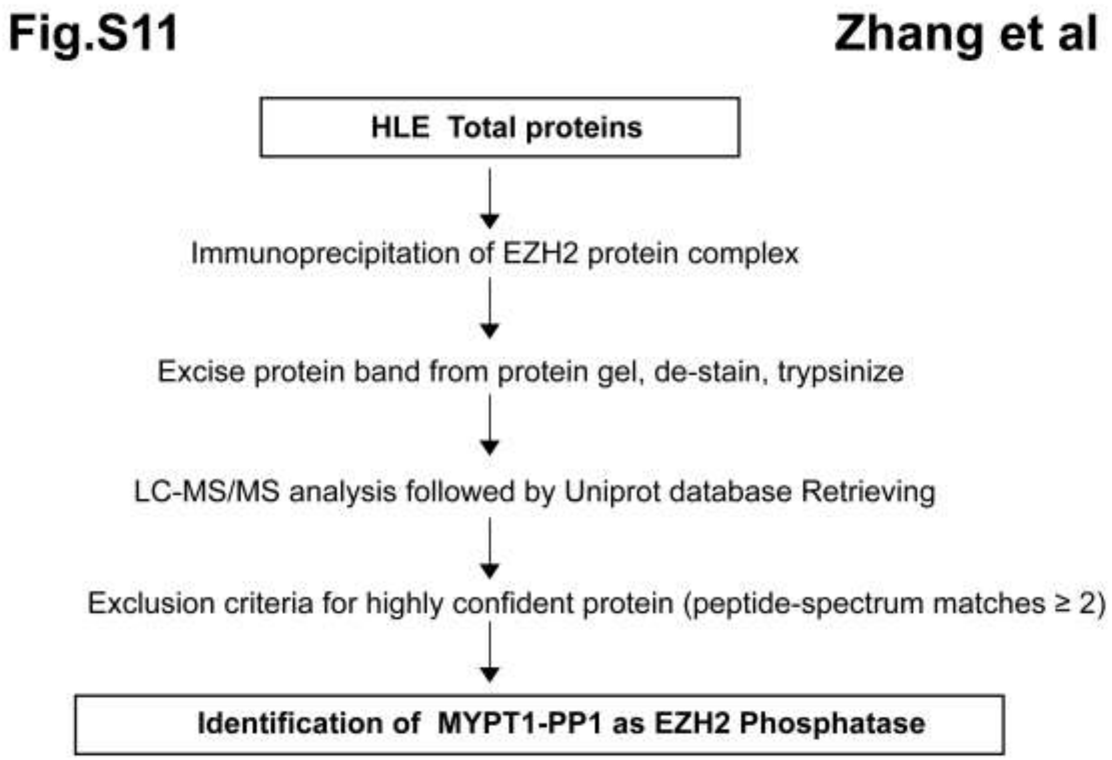


**Fig. S11. The experimental procedures and protein data screening of the co-IP linked LC-MS/MS analysis.**

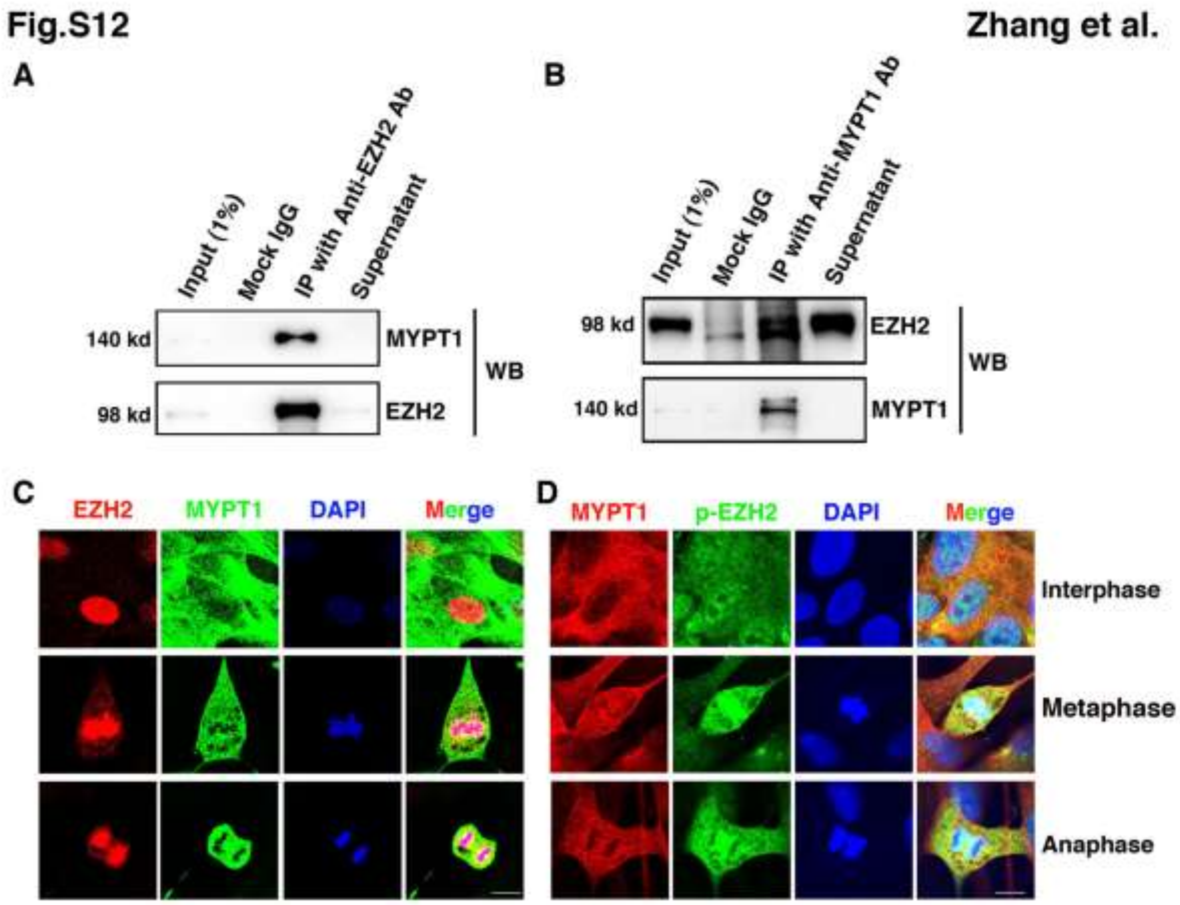


**Fig. S12. MYPT1 interacts with EZH2 in NN1003A cells. A & B.** Coimmunoprecipitation (Co-IP) of EZH2 and MYPT1 in N/N1003A cells. IgG represents a control antibody used for IPs. **C & D**. Immunofluorescence staining for EZH2 or p-EZH2 S21 and MYPT1 in N/N1003A cells. Representative images of cells at different phases of cell cycle are shown. Nuclei were stained with DAPI. Scale bar, 20μm.

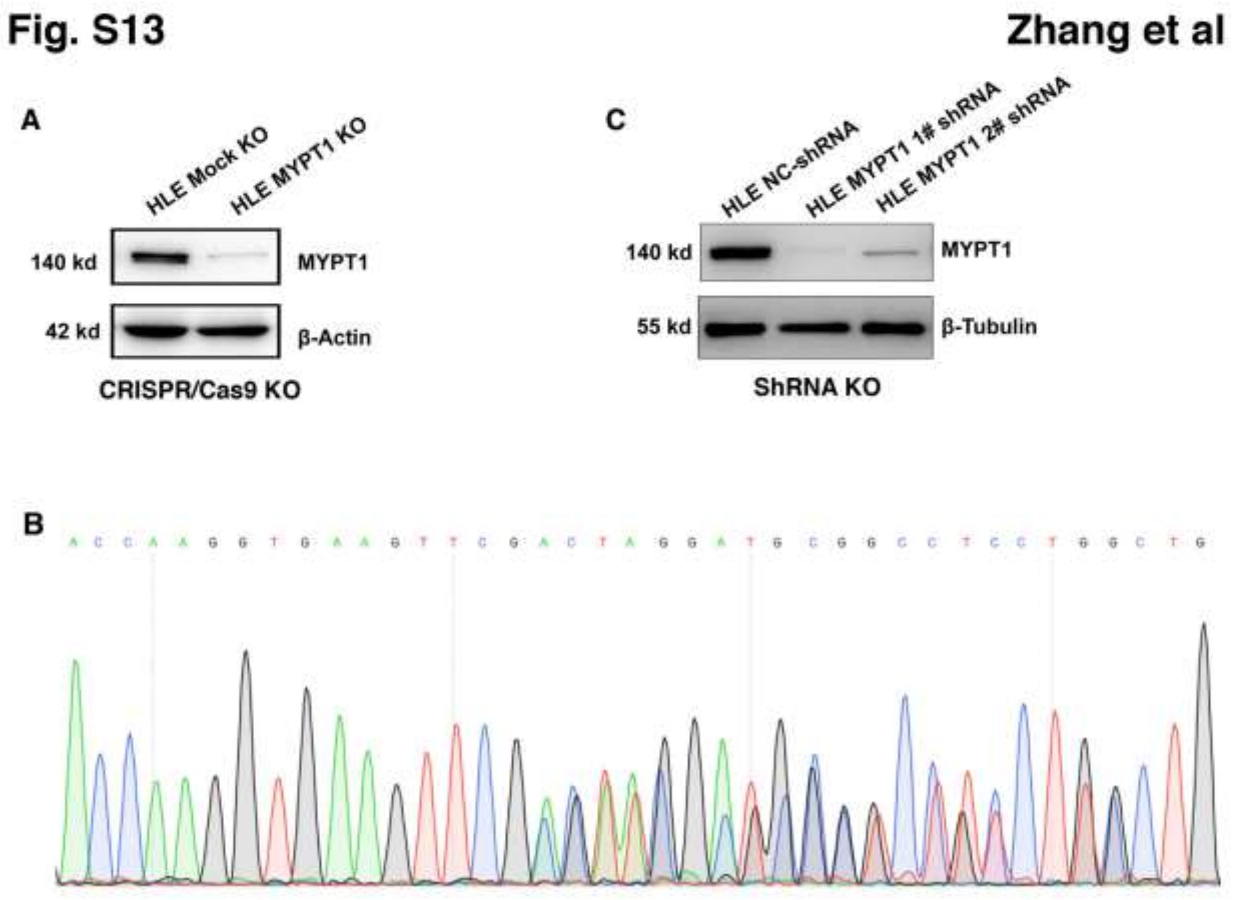


**Fig. S13. Confirmation of MYPT1 gene knockdown in HLE cells.** A. Western blot analysis of MYPT1 protein levels in gRNA vector-transfected (Mock KO) and MYPT1 gRNA-edited (MYPT1-KO) HLE cells. B. The DNA sequencing verification of the MYPT1 gRNA editing using CRISPR/Cas9 technology. Note that the MYPT1-gRNA editing site is on the 3 bp upstream of the PAM sequence TGG. **C**.Western blot analysis of MYPT1 protein levels in negative control (NC)-shRNA lentivirus, MYPT1 #1 shRNA and MYPT1 2#shRNA lentivirus infected HLE cells.

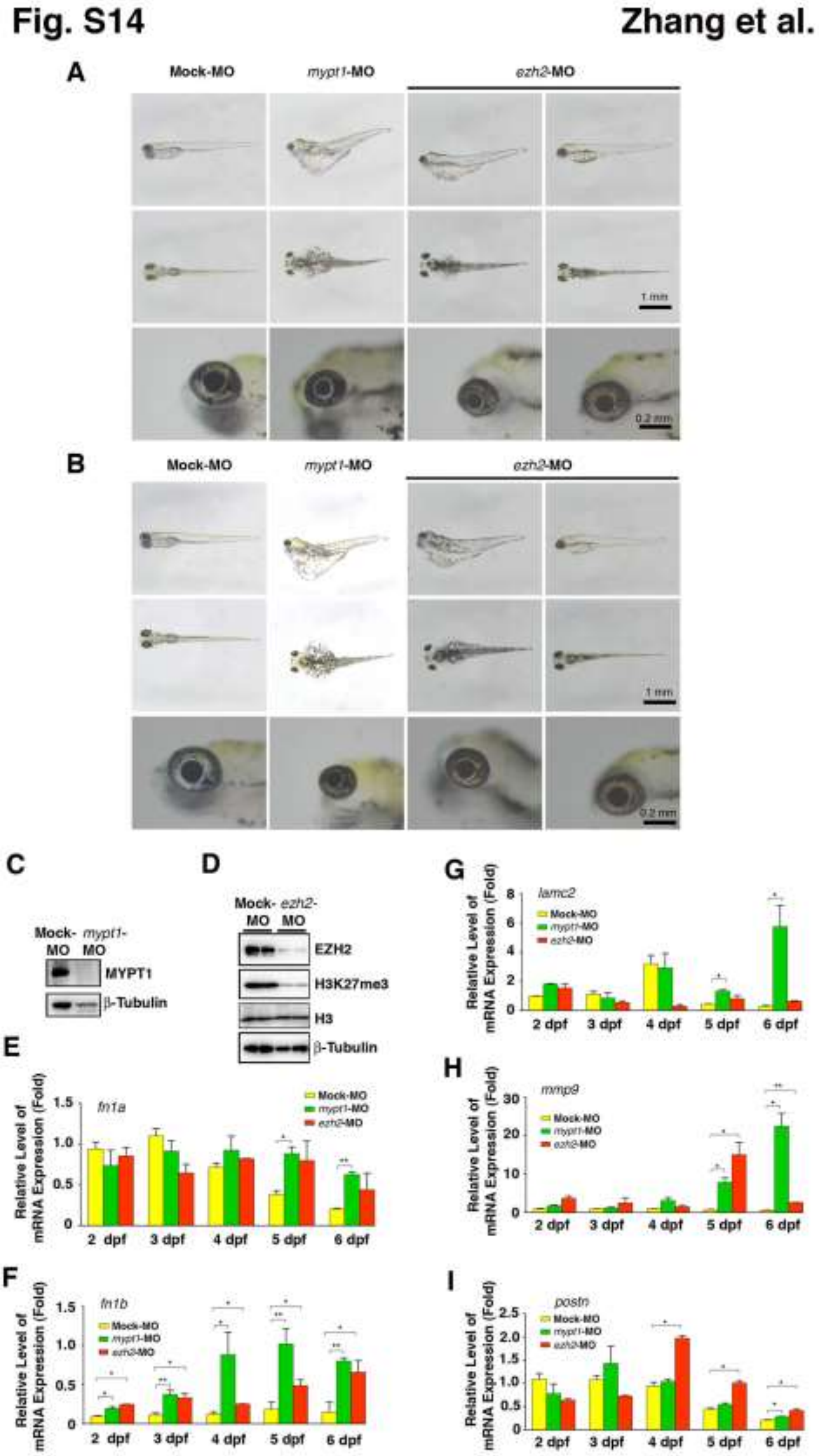
Fig. S14
Zhang et al.
A
Mock-MO
mypt1-MO
ezh2-MO
1 mm
0.2 mm
B
Mock-MO
mypt1-MO
ezh2-MO
1 mm
0.2 mm
C
Mock-MO
mypt1-MO
MYPT1
β-Tubulin
D
Mock-MO
ezh2-MO
EZH2
H3K27me3
H3
β-Tubulin
E
fn1a
Relative Level of mRNA Expression (Fold)
Mock-MO
mypt1-MO
ezh2-MO
2 dpf
3 dpf
4 dpf
5 dpf
6 dpf
F
fn1b
G
lamc2
H
mmp9
I
postn

**Fig. S14. Silencing of *mypt1* and *ezh2* with specific morpholino oligos in zebrafish caused significant upregulation of the mesenchymal genes in the eye.** (**A, B**) Representative images of whole body and eyes of *mypt1* and *ezh2* morphant larvae as well as the wild types injected with the control Morpholino on 5 dpf (**A**) and 6 dpf (**B**). (**C**) Western Immunoblotting showed that expression of *mypt1* is effectively abolished in *mypt1* Morpholino larvae on 3 dpf. (**D**) Western Immunoblotting showed that expression of *ezh2* and that of H3K27 trimethylation are effectively reduced in *ezh2* morphant larvae on 3dpf. Note that the both *mypt1* and *e*zh2 morphants demonstrated severe cardiac edema and small eyes on 5 dpf and 6 dpf. (**E - I**) qRT-PCR analysis to dertemine the temporal mRNA expression patterns of EMT marker genes *fn1a* (**E**), *fn1b* (**F**), *lamc2* (**G**), *mmp9* (**H**) and *postnb* (**I**) in the eyes of *mypt1* and *ezh2* morphant larvae as well as the wild types injected with the control Morpholino on 2 dpf, 3 dpf, 4 dpf, 5 dpf and 6 dpf. Error bars represent the standard deviation of the mean(n=2). Statistical analysis: one-way ANOVA, ** $p < 0.01$, * $p < 0.05$

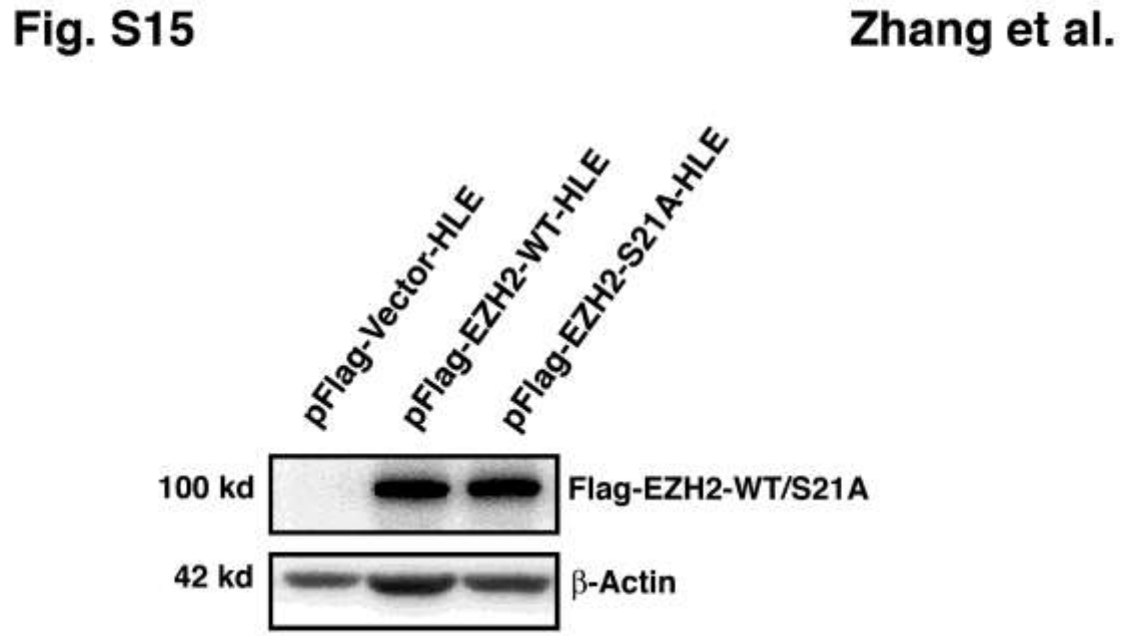


**Fig. S15. Establishment of stable HLE cell lines overexpressing WT-EZH2 and EZH2-S21A** Western blot analysis of FLAG-EZH2 fusion protein expression in stable HLE cell lines overexpressing WT-EZH2 and EZH2-S21A.

**Fig. S16** **Zhang et al.**

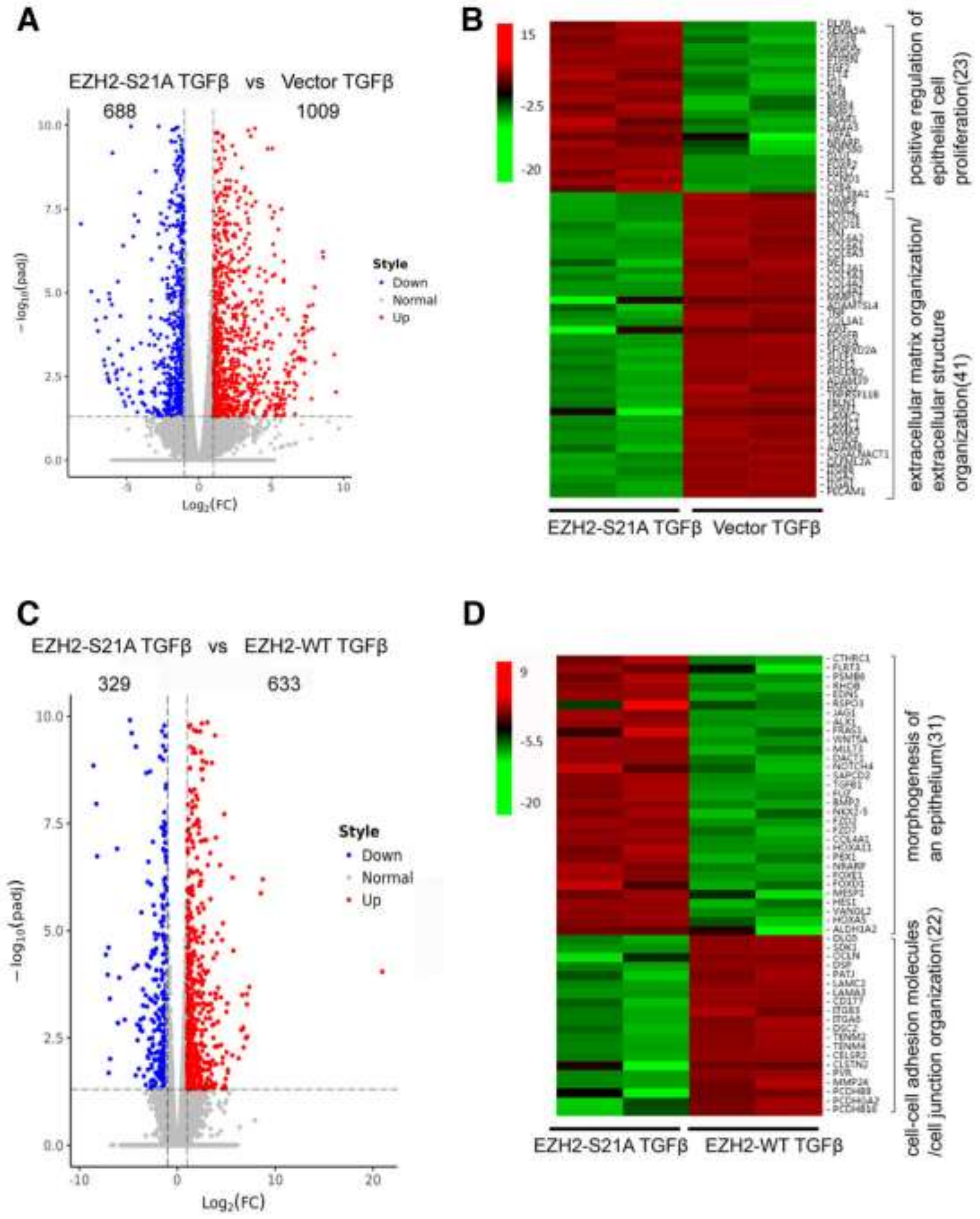

**Fig. S16. Overexpression of EZH2-S21A versus vector and WT-EZH2 versus EZH2-S21A causes differential EMT gene expression in HLE cells. A.** Volcano plot of differentially expressed genes in TGFβ-treated HLE cells overexpressing EZH2-S21A (EZH2-S21A TGFβ) and empty vector (vector TGFβ). **B.** Heatmap of the 23 upregulated genes assigned to the GO term positive regulation of epithelial cell proliferation and the 41 downregulated genes assigned to the GO term extracellular matrix organization. **C.** Volcano plot of differentially expressed genes in TGFβ-treated HLE cells overexpressing EZH2-S21A (EZH2-S21A TGFβ) and WT-EZH2 (WT-EZH2 TGFβ). **D.** Heatmap of the 31 upregulated genes assigned to the GO term morphogenesis of the epithelium and the 22 downregulated genes assigned to the GO term cell adhesion/junction in EZH2-S21A TGFβ cells compared with EZH2 TGFβ cells. In (**A**) and (**C**), two paired samples for each subject were obtained for RNA-seq analysis. The negative log10-corrected *p* values of Student's t-test are plotted against the gene expression fold changes in the 4-sample experiment. Differentially expressed genes were selected by p-value < 0.05 & log2 (fold change)> 1. The horizontal dashed bar denotes the corrected *p* values ≤ 0.05 for Student's t-test. The vertical dashed bars denote ≤0.5-fold downregulation (blue dots) or ≥2.0-fold upregulation (red dots).

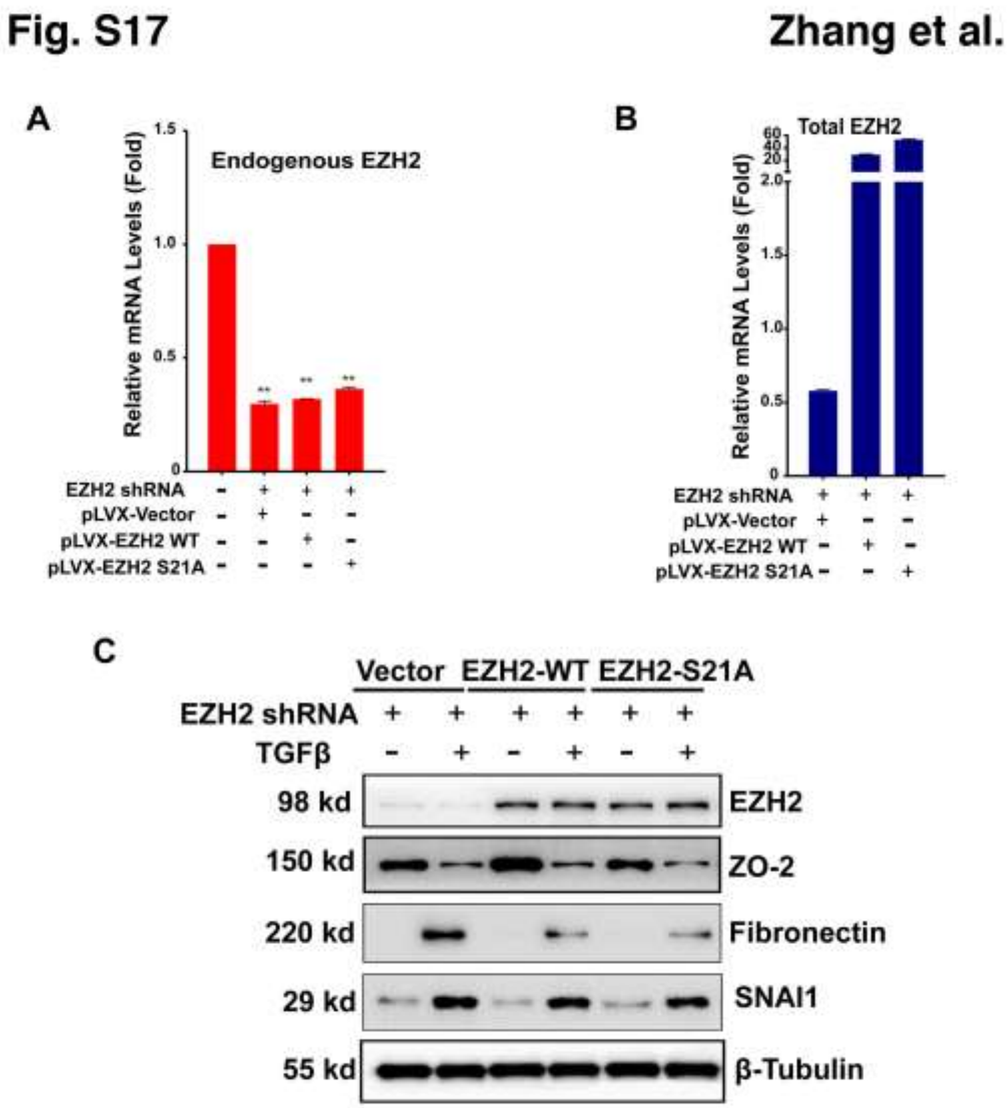


**Fig. S17. Establishment of cell lines expressing pLVX-vector (Vector), pLVX-EZH2 WT (EZH2-WT) or pLVX-EZH2-S21A (EZH2-S21A). A.** The endogenous EZH2 knockdown is conducted using pLKO lentivirus expressing shRNA targeting EZH2 3′ UTR, and the endogenous EZH2 transcripts are exclusively amplified with 3′ UTR primers. qRT-PCR was used to examine the endogenous EZH2 mRNA levels of pLKO-EZH2 shRNA lentivirus infected HLE cells overexpressing pLVX vector, EZH2 WT, or EZH2 S21A. **B.** qRT-PCR was used to examine the total EZH2 mRNA levels of endogenous-EZH2-knockdown HLE cells overexpressing pLVX vector, EZH2 WT, or EZH2 S21A. **C**. Western blot assay of EZH2 proteins, ZO-2, fibronectin, and SNAI1 in endogenous-EZH2-knockdown HLE cells overexpressing pLVX vector, EZH2 WT, or EZH2 S21A, under mock or TGFβ treatment. β-Tubulin was used as loading control.

Fig. S18 Zhang et al.

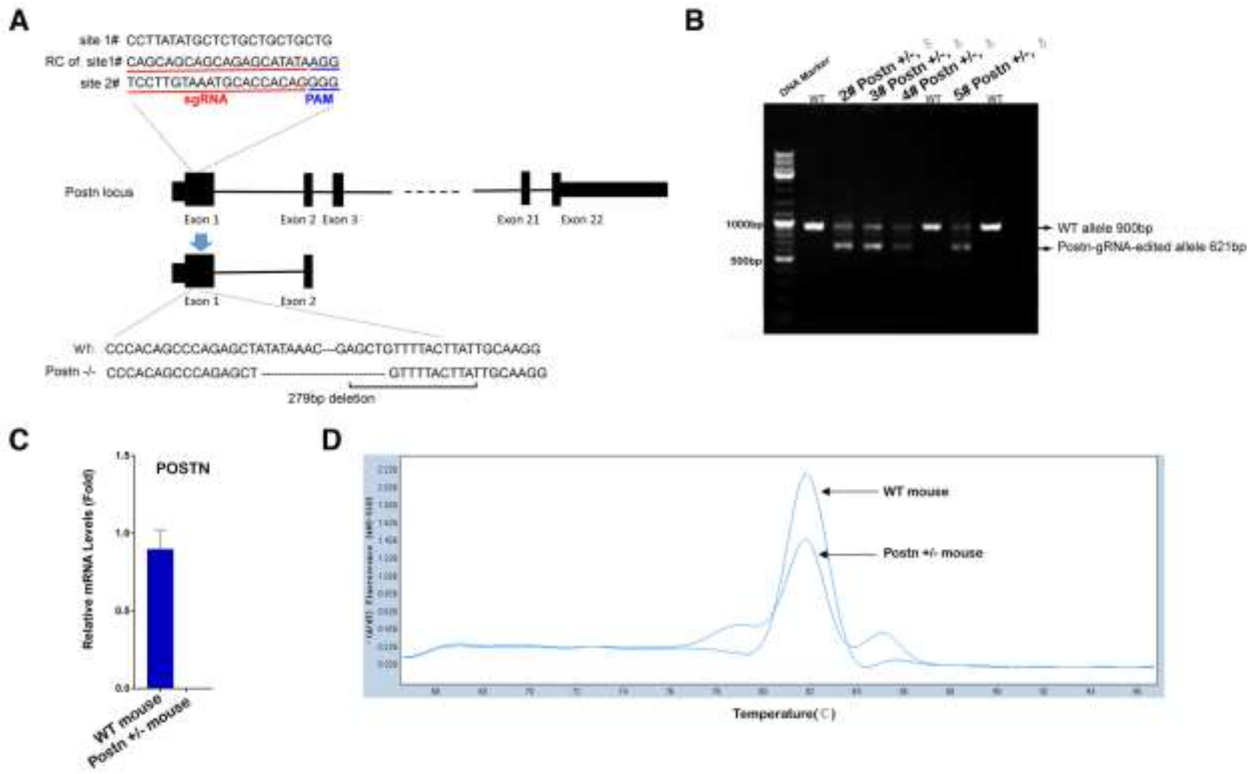


**Fig. S18. Generation and characterization of POSTN heterogeneous knockout mice.** A. Schematic diagram of the strategy for generating the POSTN heterogeneous knockout (Postn +/−)mouse strain by CRISPR/Cas9 genome editing. Two sets of sgRNAs were used to generate the knockout mice. The mapping sequence on mouse genomic DNA is shown. The Postn +/− mice had a DNA fragment deletion of 279 bp in exon 1 on one allele. PAM, protospacer adjacent motif. B. Agarose gel showing PCR products amplified form the genomic DNA derived from F1 offspring mice, which includes 5 male POSTN heterogeneous knockout (Postn +/−) mice. C. qRT-PCR examined the mRNA expression of Postn in the lens epithelium from WT and heterogeneous knockout (Postn +/−) mice. Note that, Postn mRNA level is extremely low in Postn +/− mouse. D. The melting curve of the Postn transcripts shown in C. The abnormal melting curve of the Postn transcripts from Postn +/− mouse further confirmed the results in C.

**Fig. S19** **Zhang et al.**

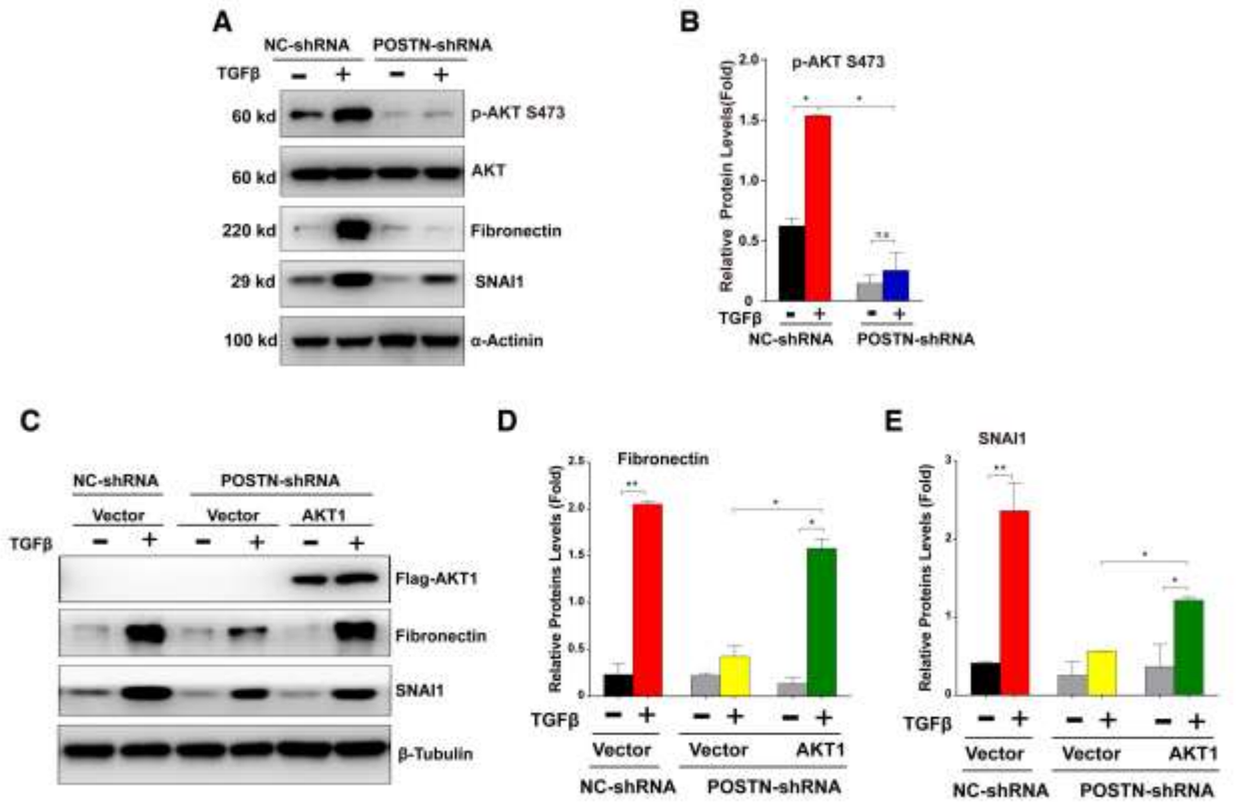


**Fig. S19. AKT1 kinase downstream of POSTN mediated mesenchymal gene activation. A.** FHL124 cells overexpressing control NC shRNA or POSTN-shRNA were treated with 20 ng $mL^{-1}$ TGFβ for 4 hr. Western blot analysis was used to determine protein levels of p-AKT S473 as well as Fibronectin and SNAI1 in the cells with indicated treatment. B. Quantification of the p-AKT S473 protein levels in (**A**). **C.** FHL124 cells overexpressing control NC shRNA or POSTN-shRNA were transiently transfected with pCMV-10 vector(Vector ) or pCMV-10 AKT1(AKT1), then the cells were treated with 20 ng $mL^{-1}$ TGFβ for 48 hr. Western blot analysis was used to determine the protein levels of Fibronectin and SNAI1 in the indicated cells. **D & E**. Quantification of the Fibronectin and SNAI1 protein levels in (**A**), respectively. In (**B**), (**D**), and (**E**), error bars represent the standard deviation of the mean (n=3). Statistical analysis: Two-way ANOVA followed by Tukey's correction. ** p < 0.01, * p < 0.05. n.s, not significant.

## 2. Supplementary Tables

### Table S1. Information of human ASC patients used in this study

| Patient sample used in Figure | Age | Gender | Number |
|---|---|---|---|
| P1, Figure 1C | 61 | Male | 1 |
| P1, Figure 1D | 63 | Male | 2 |
| P3, Figure S1A | 33 | Male | 3 |
| P4, Figure S1A | 57 | Male | 4 |
| P5, Figure S1A | 59 | Female | 5 |
| Figure 1E | 51 | Female | 6 |
| | 47 | Male | 7 |
| | 63 | Male | 8 |
| | 52 | Male | 9 |
| | 44 | Male | 10 |
| | 32 | Male | 11 |
| | 24 | Male | 12 |
| | 68 | Male | 13 |
| | 58 | Female | 14 |
| Figure 7N | 53 | Female | 15 |
| Related to Figure 7N (data not shown) | 52 | Female | 16 |
| Related to Figure 7N (data not shown) | 57 | Female | 17 |

**Tables S2. The Excel table showing the high confidential proteins identified in the complex precipitated down with anti-EZH2 antibody.**

**Table S3. Primers used for real-time quantitative PCR(qRT-PCR), Digital Droplet PCR(ddPCR) and reverse transcription PCR**

| Gene | Forward primer | Reverse primer |
|---|---|---|
| human GAPDH | GAAGGTGAAGGTCGGAGT | GAAGATGGTGATGGGATTTC |
| human FN1 | GAGCTGCACATGTCTTGGGAAC | GGAGCAAATGGCACCGAGATA |
| human CLDN1 | TGGTATGGCAATAGAATCGTTCA | CCTTGGTGTTGGGTAAGAGGTT |
| human OCLN | GGTTCACTTCTCCCAGTCTTTC | TCAACAGGGTTAGGACATTCAC |
| human TJP2 | AAGCAGAGCGAACGAAGAGTATGG | ATGACGGGATGTTGATGAGGGT |
| human KRT15 | AGGTGGTGGTAGCAGCAGCAAT | CTGAAGGCAGGGACTGGAGTTT |
| human MYO5C | CTTCAAATCTTACCTGCCAACA | AGGAATAGCCAGTTCCCATAAA |
| human MYO6 | AACGCTTCTTCCGCATCCCATTC | AGCACCACGCTTCCGAGTCAGG |
| human ITGB4 | CACCTATTTCCCTGTCTCCTCACTG | GATGGATGTTGCCCTTCTGGTC |
| human SORL1 | CACGTTCACCGTCCAAGCAAGA | CACCACCACAGCAGCAACATCC |
| human SNAI1 | GCGCTCTTTCCTCGTCAGG | GGGCTGCTGGAAGGTAAACTCT |
| human SNAI2 | AGACCCTGGTTGCTTCAAGGA | CTCAGATTTGACCTGTCTGCAAA |
| human TWIST1 | GTGGACAGTGATTCCCAGACGG | CAGTGGCTGATTGGCACGACCT |
| human TWIST2 | GTCCATGTCCGCCTCCCACTAG | TGGCAGCATCATTCAGAATCTCCT |
| human ZEB1 | GATGATGAATGCGAGTCAGATGC | ACAGCAGTGTCTTGTTGTTGT |
| human ZEB2 | GATGAGCCTCTGAACTTGACTT | TTCCGCTGGTACTTTCTCCTTT |
| human TCF3 | ACTCCTGAGACTCCCACCGTTAC | GAGGTTTCCCTTGCATCTACCAT |
| human CDH2 | AGGGGACCTTTTCCTCAAGA | CAATGTCAATGGGGTTCTCC |
| human VIM | AACTAATCTGGATTCACTCCCTCT | GAAGTTTCGTTGATAACCTGTCC |
| human CTGF | CTACAGGAAGATGTACGGAGAC | TACACTTCAAATAGCAGGCATA |
| human SERPINE1 | CCTTGCCCTTGAGTGCTTGTTA | TGGCTGGACTTCCTGAGATACG |
| human COL1A1 | CCAGAAGAACTGGTACATCAGCA | CGCCATACTCGAACTGGAATC |

| | | |
|---|---|---|
| human COL1A2 | GCGGTGGTGGTTATGACTTTGG | TGTGCGAGCTGGGTTCTTTCTA |
| human COL4A1 | ACCACGGGTACTCTTTGCTCTACG | AAGGGCATTGTGCTGAACTTGC |
| human COL4A2 | GGGTGGGACAGACGAGACAACA | CGGTATTTGGGAGAACATGGAGC |
| human COL5A1 | GGACTGCCAGATTTGGACACTA | TGACCTTTACGAGGCTTACCTT |
| human COL5A3 | AACCTCACAGATGGCAGGTGGC | GCTTATCAGCAGCTCCTGAATGTCTC |
| human COL6A3 | CGCTGGACTATGCACAGAGGTA | TCAGAGCCATCAAGCAGAAACA |
| human COL7A1 | ACTCCAGTGTCCAGGGCATCCA | CTCCGCACGGTGAGCATTGTCT |
| human SPARC | GGGCTTCTCCTCCTCTGTCTTT | ACAACCGATTCACCAACTCCAC |
| human BGN | GCAGCCAGGAGGCGGTCCATAA | AAGCGAGAAAGGTCTGAGGAAGCACA |
| human LAMC2 | ATTCCTGCCTCAGACCACTACGT | TTTCCCTTGTCAGTTGCTCCAT |
| human MMP9 | TCGTGGTTCCAACTCGGTTT | GCGGCCCTCGAAGATGA |
| human MMP17 | GAGTGGAGTGGCTAAGCAGGTTCGG | CGTCGTTATGGTCGGCCTTGGAGA |
| human POSTN | ACTTTGCTGGCACCTGTGAATA | CCGTTTCTCCCTTGCTTACTCC |
| human PCDHB8 | GGATAAGAAGGCACAAACCAGA | TTTCCTCCACCACAGAATAGCT |
| human PCDHB16 | TCGCCTGAGATAGTAGTTGCTG | CTGTTATGTTGTGCTCCGTTTT |
| human PCDHGA2 | CGCACTCAACCCAAATGACCAC | CATCAGAAGCCACGAGAACGAG |
| human PVR | AAAACTGGATTGCCTGGCTCTA | CTGGGTCTGAGTCTGGATGTGAT |
| human EZH2 (3′ UTR) | TGACATCTGCTACCTCCTCCCC | CAGAATTTCAAACTGCATGTTCTTTT |
| human EZH2 (coding region) | AGGAGTTTGCTGCTGCTCTC | CCGAGAATTTGCTTCAGAGG |
| human AKT1 | AGTGGACAACCGCCATCCAGACT | TGCCCAGCAGCTTCAGGTACTCA |
| human AKT2 | AGGCCGCGACCCAACACCTTTGT | CCTTGCTGACCGCCACTTCCATC |
| human MYPT1 | AGTTAATCGGCAAGGGGTTGA | ATGACCACTATTTAGCCACTGC |
| mouse actin | TAGGCACCAGGGTGTGATGG | CTCCATGTCGTCCCAGTTGGT |
| mouse FN1 (qRT-PCR, ddPCR) | AGACAGGACCAATGAAAGAAATCA | GTCAGTGCCTGGCTGTAAACCT |
| mouse ACTA2(ddPCR) | CATCCACGAAACCACCTATAACA | AGCCACCGATCCAGACAGAGTA |
| mouse COL1A1(ddPCR) | GAGCCTGGCGGTTCAGGTCCAA | AGCCTCGGTGTCCCTTCATTCC |
| mouse COL1A1 | AACCTAACCATCTGGCATCTCC | GTTTCCAGTCTGCTGTGACCCT |
| mouse MMP9 | AGGGTACAGCCTGTTCCTGGTG | GGATGCCGTCTATGTCGTCTTTATT |
| mouse POSTN | TATTGAGATAGGGTGCGAAGGG | GTCGGTGAAAGTGGTTTGCTGT |

| | | |
|---|---|---|
| mouse BGN | CTCCCTGAGACCCTGAACGAAC | CCTCTTGACTCCGAAGCCCATA |
| mouse PCDHB16 | TGAGATGTAAGATGTGGTGCCT | CTTGGGTATTGAAAGTGGTAGA |
| mouse COL7A1 | AAGGGTGACCAAGGCGAGAAAG | CCTGACGCACAAAGTCCCGAAT |
| zebra fish ACTB2 | CGAGCTGTCTTCCCATCCA | TCACCAACGTAGCTGTCTTTCTG |
| zebra fish fn1a | GACGCCAGTTTGACAGGTTATCG | TTTGACACGAGGGCTGGTTAGAG |
| zebra fish fn1b | GTGGTGGGCGTGCTAAAGATGAG | GGAGATGGTAATGCTGGTGGGTG |
| zebra fish lamc2 | CTGGCACCTTGTCTGATCTGGTT | CACTTTATTTACATCCGCTTCATACTC |
| zebra fish mmp9 | AACCACCGCAGACTATGACAAGG | GTTTCCACCAATCACAGCCGTAT |
| zebra fish postnb | ATGAGACCCCAGGCTGAGT | TCCATGGACATCACCTCATC |

**Table S4. Primers used for human EZH2 gene cloning and mutagenesis, and for verification of gRNA editing**

| Gene | Forward primer | Reverse primer |
|---|---|---|
| human EZH2-WT | GGACTAGTCCGCCACCATGGATTACAAGGATGACGATGACAAGCGAAGAATAATCATGGGCCAGACTGGGAAGA | CGGGATCCGCATCTAGGAGGTAGCAGATGTCAAGGGA |
| human EZH2-S21A | TGGCGGAAGCGTGTAAAAGCAGAGTACATGCGAC | GTCGCATGTACTCTGCTTTTACACGCTTCCGCCA |
| human EZH2-S21D | TTTGTTGGCGGAAGCGTGTAAAAGATGAGTACATGCGACTGAGACA | TGTCTCAGTCGCATGTACTCATCTTTTACACGCTTCCGCCAACAAA |
| human AKT1 | GCGAATTCAATGAGCGACGTGGCTATTGTGAA | GCGGATCCCCCGGAGAACAAACTGGATGAAA |
| human MYPT1 | TGGCCCGCACTCATAGAAACATT | CCAGCCTCAAGAGGTGGAGAACTG |
| mouse Postn | TGCTCAGAACCCAGGAGAT | TTAGGGAGGAATGCCAAGA |
